# A Connected Network of Interacting Proteins Is Involved in Human-Tau Toxicity in *Drosophila*

**DOI:** 10.3389/fnins.2020.00068

**Published:** 2020-02-11

**Authors:** Sébastien Feuillette, Camille Charbonnier, Thierry Frebourg, Dominique Campion, Magalie Lecourtois

**Affiliations:** ^1^UNIROUEN, Inserm U1245, CNR-MAJ, F 76000, Department of Genetics, Normandy Center for Genomic and Personalized Medicine, Rouen University Hospital, Normandie Université, Rouen, France; ^2^Centre Hospitalier du Rouvray, Sotteville-lès-Rouen, France

**Keywords:** Tau, tauopathies, *Drosophila*, protein network analysis, presynaptic

## Abstract

Tauopathies are neurodegenerative diseases characterized by the presence of aggregates of abnormally phosphorylated Tau. Deciphering the pathophysiological mechanisms that lead from the alteration of Tau biology to neuronal death depends on the identification of Tau cellular partners. Combining genetic and transcriptomic analyses in *Drosophila*, we identified 77 new modulators of human Tau-induced toxicity, bringing to 301 the number of Tau genetic interactors identified so far in flies. Network analysis showed that 229 of these genetic modulators constitute a connected network. The addition of 77 new genes strengthened the network structure, increased the intergenic connectivity and brought up key hubs with high connectivities, namely *Src64B/FYN, Src42A/FRK, kuz/ADAM10, heph/PTBP1, scrib/SCRIB*, and *Cam/CALM3.* Interestingly, we established for the first time a genetic link between Tau-induced toxicity and *ADAM10*, a recognized Alzheimer Disease protective factor. In addition, our data support the importance of the presynaptic compartment in mediating Tau toxicity.

## Introduction

The microtubule-associated protein Tau, encoded by the *MAPT* gene, has been associated with multiple neurodegenerative disorders, including Alzheimer’s disease (AD), fronto-temporal dementia with parkinsonism linked to chromosome 17 (FTDP-17), Pick’s disease (PiD), corticobasal degeneration (CBD), and progressive supranuclear palsy (PSP). These disorders, collectively known as tauopathies, are characterized by the accumulation of intracellular filamentous inclusions composed of aberrantly post-translationally modified Tau proteins. The identification of mutations in the *MAPT* gene in autosomal dominant FTDP-17 demonstrated that the dysregulation or dysfunction of Tau are sufficient to cause neurodegeneration (Strang et al., 2019).

Tau is a multifunctional protein, originally identified as a cytoplasmic protein associated with microtubules. In addition to its microtubule-stabilizing properties, recent studies have highlighted new roles of Tau in different neuronal compartments, such as DNA/RNA protection, maintenance of the integrity of genomic DNA, stability of pericentromeric heterochromatin, regulation of neuronal activity, and synaptic plasticity (Sotiropoulos et al., 2017). Its biological activity is highly regulated by its phosphorylation state. In addition to phosphorylation, several other post-translational modifications of Tau and protease-mediated cleavage have been reported and may contribute differentially to physiological functions of Tau and disease (Tapia-Rojas et al., 2019). However, our knowledge of the exact molecular pathways in which Tau exerts its cellular functions, and their potential involvement in neuropathology, remain limited.

Various *Drosophila* models have been successfully developed to investigate the molecular basis of Tau pathogenesis (Sivanantharajah et al., 2019). Pan-neuronal over-expression of wild-type or mutated human Tau isoforms in *Drosophila* recapitulates some key pathological features of human tauopathies, including neuronal loss, progressive motor deficits and neurodegeneration, premature death and accumulation of abnormally phosphorylated forms of Tau. Manipulating Tau expression in mushroom bodies, the brain center for learning and memory in insects, results in detrimental effects on associative olfactory learning and memory (Mershin et al., 2004). When targeted in retinal cells, human Tau proteins cause alterations of the external eye structure, inducing a rough eye phenotype (REP) that correlates with photoreceptor axons degeneration and loss of retinal cells (Prüßing et al., 2013).

Given its facility of tracking and thanks to a wide variety of available genetic tools, the REP has been widely used by several groups – including ours – since 2003 to perform large-scale misexpression screens in *Drosophila* to identify genes involved in Tau toxicity (Supplementary Table S1). Briefly, using either an unbiased design or focusing on specific sets of genes with particular molecular functions, *Drosophila* overexpressing human Tau protein in retina were crossed with mutant strains, and modulation of the REP in the progeny was used as read out. Up to now, this strategy has led to the identification of 224 genetic modifiers of Tau-mediated cellular toxicity (Supplementary Table S1) and pointed-out that the key cellular processes involved in this toxicity are mainly related to phosphorylation, proteostasis, cytoskeleton organization, gene expression, cell cycle, chromatin regulation, and apoptosis (Hannan et al., 2016).

In the present report, combining genetic and transcriptomic analyses in *Drosophila*, we identified 77 new genetic modifiers of human Tau toxicity, bringing to 301 the number of Tau-genetic interactors identified so far in flies. Network analysis revealed that 229 of them constitute a connected network. Interestingly, the addition of these 77 new modulators strengthened the network structure, increased the intergenic connectivity and brought up key hubs with very high connectivities.

## Materials and Methods

### *Drosophila* Genetics

Unless otherwise stated, the Gal4 driver lines and the mutant strains were obtained from the Bloomington *Drosophila* stock center (BDSC) (Indiana University, Bloomington, IN, United States). *UAS-Tau0N4RWT* and *UAS-dTau-1D4* have already been described (Wittmann et al., 2001; Feuillette et al., 2010). The *UAS-Cam* line was kindly provided by Dr. M. L. Parmentier (IGF, Montpellier, France). The *GMR-Gal4* > *hTau*^*WT*^ fly model of tauopathy expresses the wild-type form of human 0N4R Tau protein in the entire retina. The *elav-Gal4^*GS*^* > *hTau*^*WT*^ fly model allows the inducible expression of the wild-type form of human 0N4R Tau in all post-mitotic neurons. *Drosophila* strains were raised on a 12:12 light/dark cycle on standard cornmeal-yeast agar medium. Fly cultures and crosses were carried out at 25°C.

### REP Modification Assessment

Screening was performed using a screening stock with eye-specific Tau expression: *GMR-Gal4* > *hTau*^*WT*^. The *GMR-Gal4* line drives expression in all cells of the eyes, including the photoreceptor neurons. Note that human Tau proteins are therefore expressed only in the presynaptic compartment of photoreceptors. *GMR-Gal4* > *hTau*^*WT*^ or *GMR-Gal4* > + control female flies (not expressing Tau) were crossed with males carrying mutant alleles relevant to candidate modifier genes, and the F1 generation was screened for robust changes in the Tau-dependent REP. Our screen was carried out in blinded phenotypic scoring. Mutant lines were initially known only by their stock number. Screeners did not have access to molecular identity of relevant loci during the screening procedure. Informations on the affected gene were obtained only after the F1 phenotypes were scored for modifying effect on the Tau eye phenotype. To overcome inter-individual variability, 2 independent batches of flies (>20 flies each) were used to determine REP severity. A gene was called a suppressor if the eye was larger, less rough or displayed a significant amelioration of the ommatidial irregularity compared to control eye phenotypes. Enhancers were identified if the eye was smaller, showed strong changes in morplogical eye volume, or had increased ommatidial fusion and bristle loss. A gene was also called an enhancer if necrotic patches were present even if the eye was not smaller when compared with controls, as necrotic patches were never observed in controls. Only the mutant alleles that induced a modulation of the REP in the *GMR-Gal4* > *hTau*^*WT*^ genetic context, but not in *GMR-Gal4* > + control flies were considered as suitable hits (see Supplementary Table S2). Only the most robust and reliable modifiers of Tau toxicity were included. For REP modification imaging, adult flies were frozen then thawn at room temperature prior to light microscopy using a Leica APO Z6 macroscope (Leica, Wetzlar, Germany) equipped with a Leica DFC320 digital camera controlled with the Leica LAS V4.8 software. Z stacks were generated for each sample to record images at different focal planes. Focus stacking was performed using Zerene Stacker (Zerene Systems, Richland, WA, United States) with the PMax stacking method, and images were then converted in gray scale, cropped and orientated using Fiji environment (Schindelin et al., 2012)^1^ (RRID:SCR_002285).

### *Drosophila* Primary Neuronal Culture

*Drosophila* primary neuronal cultures were derived from third instar larval brains as previously described (Feuillette et al., 2017). Briefly, third instar larvae were collected, and then sequentially washed twice with absolute ethanol and twice with sterile water. The brains were dissected in Rinaldini’s buffer (RB) and washed four times with RB. After 1 h at room temperature with collagenase (200 μg/mL in RB) (Sigma-Aldrich, Saint-Louis, MO, United States), brains were washed three times (5 min each) with Schneider’s *Drosophila* cell culture medium (Thermo Fisher Scientific, Waltham, MA, United States) supplemented with 20% fetal calf serum (FCS) (Eurobio, Courtaboeuf, France)/insulin (2 μg/mL) (Sigma-Aldrich). By pipetting up and down, the brains were then dissociated in culture medium and cellular suspensions equivalent to 3.5 brains were plated on precoated 15-mm diameter coverslips (mix of concanavalin A and laminin, Sigma-Aldrich) and incubated for 2 h at 25°C in a humidified 5% CO2 atmosphere for cell adhesion. Cell cultures were then left to grow at 25°C in culture medium supplemented with amphotericin B (2.5 μg/mL) (Sigma-Aldrich) and penicillin (100 units/ml)/streptomycin (100 μg/ml) (Sigma-Aldrich), and 15 μM RU486 (Mifepristone, Betapharma-Shanghai Co., Ltd, China) to induce the expression of the *UAS* reporter construct. RU486 was diluted beforehand to a final stock concentration of 1.5 mg/mL in a 25% 2-hydroxypropyl-β-cyclodextrin solubilizing solution (Sigma-Aldrich) (Lahiani-Skiba et al., 2006) to improve its solubility and bioavailability in aqueous buffer. *Drosophila* primary neuronal cultures were derived from *elav-Gal4^*GS*^* > + and *elav-Gal4^*GS*^* > *hTau*^*WT*^ larvae.

### Immunofluorescence Microscopy

As previously described (Feuillette et al., 2017), *Drosophila* primary neuronal cultures *elav-Gal4^*GS*^* > + and *elav-Gal4^*GS*^* > *hTau*^*WT*^ were first washed two times with phosphate-buffered saline (PBS) and then fixed in PBS/4% paraformaldehyde for 15 min at room temperature. After three PBS washes, neurons were permeabilized with PBS/0.1% Triton X-100 for 5min, and then blocked for 30 min in antibody buffer (PBS/2% BSA). Next, neurons were incubated with primary antibodies in antibody buffer for 1 h at room temperature. After three PBS rinses, neurons were labeled with fluorescent-conjugated antibodies diluted to 1:600 in antibody buffer for 1 h at room temperature. After three PBS rinses, neurons were counterstained with DAPI (NucBlue^TM^ Fixed Cell ReadyProbes reagent, Thermo Fisher Scientific) and coverslips were finally mounted in ProLong^TM^ Diamond (Thermo Fisher Scientific). We used the following antibodies: goat anti-horseradish peroxydase (HRP) (1:500; Sigma-Aldrich; RRID:AB_1840055), rabbit anti-Tau (1:1000; Agilent Dako, Santa Clara, CA, United States; RRID:AB_10013724), donkey anti-rabbit (ThermoFischer SCIENTIFIC; RRID:AB_2535792), and donkey anti-goat (ThermoFischer SCIENTIFIC; RRID:AB_2534105). Images were acquired using a Axioplan2 fluorescence microscope (Carl-Zeiss, Oberkochen, Germany) equipped with a digital AxioCam MRm camera (Carl-Zeiss) controlled by AxioVision software (Carl-Zeiss).

### Total RNA Isolation

For each *Drosophila* primary culture type, mRNA isolation was achieved at DIV3 from two 15-mm diameter dishes after two washes with PBS 1X using NucleoSpin RNA XS kit (Macherey-Nagel, Düren, Germany) according to manufacturer. Before being further processed, the concentration of RNA samples was measured by spectrophotometry and their quality was checked using an Agilent 2100 Bioanalyzer (Agilent Technologies, Santa Clara, CA, United States). Total RNA isolation was performed on the following culture types: *elav-Gal4^*GS*^* > + and *elav-Gal4^*GS*^* > *hTau*^*WT*^.

### *Drosophila* Gene Expression Microarrays

Comparative gene expression profilings of *elav-Gal4^*GS*^ Drosophila* primary neuronal cultures expressing or not the human proteins Tau^*WT*^ were performed using *Drosophila* Gene Expression 4 × 44K Microarrays (G2519F-021791, Agilent Technologies), according to the Agilent Two-Color Gene Expression workflow. Briefly, starting from 50 ng of total RNA, cRNA were synthesized and labeled using the low-input Quick Amp Labeling Kit (Agilent Technologies), with Cy3 (for *elav-Gal4^*GS*^* > + control culture) and Cy5 (for *elav-Gal4^*GS*^* > *hTau*^*WT*^ cultures) and purified using the RNeasy Protect mini kit (Qiagen, Hilden, Germany) following manufacturer’s instructions. Following co-hybridization of 825 ng of cRNA Cy3-labeled and 825 ng of cRNA Cy5-labeled on microarrays, fluorescence signals were detected using an Agilent’s DNA microarray scanner G2565CA (Agilent Technologies) with a resolution of 5 μm. Comparative gene expression profiling was performed in 2 replicates. The datasets generated for this study can be found in the ArrayExpress database (RRID:SCR_002964)^2^ under the accession number E-MTAB-8712.

### Comparative Gene-Expression Profiling

GeneSpring GX 14.9.1 software (Agilent Technologies) was used to select on replicates the differentially expressed genes considering only probes with *p*-value (logRatio) ≤ 0.01 and absolute fold-change greater than 1.5.

### Statistical Analysis

Statistical tests and chart representations were performed using GraphPad Prism (RRID:SCR_002798) and R (RRID:SCR_001905). Statistic details of experiments, including test used, were mentioned in the figures legends.

### Neuronal Processes Length Quantification

Measures of average length of extensions *per* neuron in control *elav-Gal4^*GS*^* > + primary cultures or in *elav-Gal4^*GS*^* > *hTau*^*WT*^ cultures with neurons expressing human Tau were performed post acquisition on HRP immunostaining (in gray scale) using the NeuriteTracer plugin (Pool et al., 2008) (RRID:SCR_014146) on Fiji environment (RRID:SCR_002285). For each culture type, measurements were performed on 5 replicates totaling more than 900 neurons.

### Genetic and Physical Interactors Retrieval

Easy networks (Bean et al., 2014)^3^ database was used to automatically retrieve genetic and physical interactors of *Drosophila* gene lists gathering comprehensive interactions evidences from FlyBase and BioGRID databases. For this particular task and in our hand, the choice of EsyN was dictated by its better ability to retrieve the highest number of interactors compared to the number collected with STRING described below.

### Network Analysis

Search tool for the retrieval of interacting genes/proteins (STRING) (Szklarczyk et al., 2019)^4^ (RRID:SCR_005223) was used to construct physical and functional interaction networks among the genetic modifiers of Tau toxicity. Active interaction sources were restricted to “Textmining,” “Experiments,” and “Databases.” Note that, in our hand, STRING, based on specific features not included in EsyN (computational prediction, knowledge transfer between organisms and interactions aggregated from other primary databases), was more adapted in this task, leading to the construction of a denser network. Only interactions with confidence score over 0.5 were mapped to the network which was imported into the Cytoscape V3.7.1 software platform (Shannon et al., 2003)^5^ (RRID:SCR_003032). Network statistics were performed using the NetworkAnalyzer plugin (Assenov et al., 2008) and network clustering was realized with the ClusterMaker2 plugin (Morris et al., 2011) using the Community clustering algorithm (GLay) (Su et al., 2010) for partitioning nodes into similar groups.

### Functional Enrichment Analysis

The functional annotation enrichments of gene lists were calculated using Database for annotation, visualization, and integrated discovery (DAVID) (Huang et al., 2009a, b)^6^ (RRID:SCR_001881) and querying for the biological processes-related gene ontology (GO) Direct terms (GO mappings excluding parent terms). An EASE score of 0.05 (a modified Fisher Exact Test) was used for hypergeometric testing, followed by the Benjamini correction for multiple hypothesis test adjustment. The threshold of significance was set to *p*-value ≤ 0.05.

### Human Orthologs Identification

*Drosophila* RNAi Screening Center Integrative Ortholog Prediction Tool (DIOPT) (Hu et al., 2011)^7^ was used to identify human orthologs of *Drosophila* genes selecting all the prediction tools and returning only best matches when there was more than one match per input gene. DIOPT-Diseases and Traits (DIOPT-DIST) (Hu et al., 2011)^8^ allowed the identification of human disease genes restricting to those described in the OMIM database.

### Human Genes Constraint Metrics Retrieval

To depict the intolerance to haploinsufficiency/inactivation of the human orthologs of the genetic modifiers of Tau toxicity identified in *Drosophila*, the observed/expected ratios of predicted loss-of-function (pLoF) variations (o/e), continuous constraint metrics for the human genome, were downloaded from the Genome Aggregation Database (GnomAD) (Lek et al., 2016; Karczewski et al., 2019)^9^ (RRID:SCR_014964). The LoF o/e ratio is a stringent metric correlating with biological relevance (protein-protein interactions, gene expression, and disease associated) and robustly distinguishing genes based on their sensitivity to genetic disruption.

## Results

### A Genetic Screen Identifies 59 Novel Modifiers of Tau Toxicity in *Drosophila*

In 2007, we performed a misexpression screen in *Drosophila* to identify genetic modifiers of human Tau toxicity using REP as a read-out. The screening of a collection of 1250 mutant *Drosophila* lines containing P{Mae-UAS.6.11}-transposable elements permitted the identification of 30 genetic interactors, among which were several components of the cytoskeleton (Blard et al., 2007). Beside this list, we identified numerous additional mutant *Drosophila* lines for which the genomic mapping was inaccurate. Recently, by using the latest releases of the *Drosophila* genome, we were able to refine the insertion point of the transposon in these additional mutant lines, allowing the identification of new candidate genes. Using independent mutant alleles (Supplementary Table S2), we undertook the reevaluation of these new candidate loci in a *GMR-Gal4* > *hTau*^*WT*^ fly model of tauopathy expressing the wild-type form of the human Tau protein in the entire retina. We found that genetic manipulations of 59 novel genes robustly modified Tau-induced neurodegeneration in *Drosophila* (Table 1, Supplementary Figure S1, and Supplementary Table S2). We confirmed that these mutant alleles did not produce offspring with REP after being crossed to the *GMR-Gal4* driver line alone (data not shown). These 59 novel genes, when added to the 224 genetic interactors already identified in previous studies (Supplementary Table S1), brought at this stage the total number of Tau genetic modifiers to 283. Interestingly, most of these new genes fitted into the key cellular processes previously described for modifiers of Tau toxicity (Hannan et al., 2016): phosphorylation, proteostasis, cytoskeleton organization, gene expression, cell cycle, chromatin regulation, and apoptosis (see Table 1 for annotations of molecular functions).

**TABLE 1:** Identification of 59 novel genetic modifiers of Tau toxicity in *Drosophila*.

**Gene symbol**	**Gene name**	**Molecular function**	**Human orthologs**
*asp*	Abnormal spindle	Microtubule binding; myosin light chain binding; calmodulin binding	*ASPM*
*bbg*	Big bang	–	*IL16*
*bnl*	Branchless	Growth factor activity; fibroblast growth factor receptor binding; chemoattractant activity	*FGF16*
			*FGF20*
*Cam*	Calmodulin	Protein binding; myosin V binding; calcium ion binding; myosin heavy chain binding; myosin VI head/neck binding	*CALM3*
*CG12935*	–	–	*TMEM223*
*CG1806*	–	–	*SSPN*
*CG30015*	–	–	*–*
*CG31886*	–	–	*–*
*chb*	Chromosome bows	Kinetochore binding; microtubule plus-end binding; microtubule binding; GTP binding	*CLASP1*
*cpx*	Complexin	Syntaxin binding; neurotransmitter transporter activity; SNARE binding	*CPLX1*
*Dmtn*	Dementin	–	*TMCC1*
			*TMCC2*
*dpr1*	Defective proboscis extension response 1	–	*–*
*dpr18*	Defective proboscis extension response 18	–	*–*
*eIF4EHP*	Eukaryotic translation initiation factor 4E homologous protein	Protein binding; RNA 7-methylguanosine cap binding; translation initiation factor activity; translation repressor activity; eukaryotic initiation factor 4G binding	*EIF4E2*
*ena*	Enabled	Protein binding; SH3 domain binding; actin binding	*ENAH*
*ens*	Ensconsin	Microtubule binding	*MAP7*
			*MAP7D1*
			*MAP7D2*
			*MAP7D3*
*Fer1HCH*	Ferritin 1 heavy chain homolog	Ferrous iron binding; identical protein binding; iron ion binding; ferroxidase activity	*FTH1*
			*FTHL17*
			*FTMT*
*fs(1)h*	Female sterile (1) homeotic	–	*BRD2*
			*BRD3*
			*BRD4*
			*BRDT*
*gpp*	Grappa	Histone methyltransferase activity (H3-K79 specific)	*DOT1L*
*Gr47b*	Gustatory receptor 47b	Taste receptor activity	*-*
*h*	Hairy	Protein dimerization activity; E-box binding; protein binding; DNA-binding transcription repressor activity, RNA polymerase II-specific; sequence-specific double-stranded DNA binding; transcription factor binding; RNA polymerase II proximal promoter sequence-specific DNA binding; transcription corepressor activity	*HES4*
*haf*	Hattifattener	–	*TRIL*
*hdc*	Headcase	–	*HECA*
*heph*	Hephaestus	Translation repressor activity, mRNA regulatory element binding; mRNA 3’-UTR binding	*PTBP1*
*His2A:CG31618*	His2A:CG31618	Protein heterodimerization activity; DNA binding	*HIST1H2AA*
			*HIST1H2AC*
			*HIST2H2AA4*
			*HIST2H2AC*
			*HIST3H2A*
*IP3K1*	Inositol 1,4,5-triphosphate kinase 1	Kinase activity; calmodulin binding	*ITPKA*
*jumu*	Jumeau	DNA-binding transcription factor activity; transcription regulatory region sequence-specific DNA binding	*FOXN1*
			*FOXN4*
*kay*	Kayak	RNA polymerase II proximal promoter sequence-specific DNA binding; protein binding; repressing transcription factor binding; DNA-binding transcription factor activity, RNA polymerase II-specific; sequence-specific DNA binding; protein heterodimerization activity	*FOS*
			*FOSL1*
			*FOSL2*
*kuz*	Kuzbanian	Metalloendopeptidase activity; Notch binding	*ADAM10*
*l(3)L1231*	Lethal (3) L1231	–	*INO80D*
*lncRNA:CR31044*	Long non-coding RNA:CR31044	–	*-*
*mam*	Mastermind	Transcription coactivator activity	*MAML1*
*Mbs*	Myosin binding subunit	Protein kinase binding; enzyme inhibitor activity; phosphatase regulator activity	*PPP1R12B*
*Meltrin*	Meltrin	Identical protein binding; metalloendopeptidase activity	*ADAM12*
*mura*	Murashka	Zinc ion binding; ubiquitin-protein transferase activity	*RNF38*
*Not1*	Not1	Protein-containing complex scaffold activity; protein binding; poly(A)-specific ribonuclease activity	*CNOT1*
*NSD*	Nuclear receptor binding SET domain protein	Chromatin DNA binding; histone methyltransferase activity (H3-K36 specific)	*NSD3*
*numb*	Numb	Protein binding; Notch binding	*NUMBL*
*Oct-TyrR*	Octopamine-Tyramine receptor	Octopamine receptor activity; adrenergic receptor activity; G protein-coupled amine receptor activity	*ADRA2A*
			*ADRA2B*
			*ADRA2C*
			*HTR1D*
			*HTR1A*
*Pdk1*	Phosphoinositide-dependent kinase 1	ATP binding; 3-phosphoinositide-dependent protein kinase activity; protein serine/threonine kinase activity; protein kinase activator activity	*PDPK1*
*Piezo*	Piezo	Mechanosensitive ion channel activity; cation channel activity	*PIEZO2*
*pyd*	Polychaetoid	Cell adhesion molecule binding	*TJP1*
			*TJP2*
*Rab14*	Rab14	GTP binding; GTPase activity	*RAB14*
*raw*	Raw	–	*RNASEL*
*RyR*	Ryanodine receptor	Calcium-induced calcium release activity; ryanodine-sensitive calcium-release channel activity; calcium ion binding	*RYR1*
			*RYR2*
*scrib*	Scribble	Ionotropic glutamate receptor binding; protein binding	*SCRIB*
*sdt*	Stardust	Protein binding; guanylate kinase activity	*MPP5*
*SelD*	Selenide,water dikinase	ATP binding; selenide, water dikinase activity	*SEPHS1*
*shn*	Schnurri	Zinc ion binding; transcription coactivator activity; DNA-binding transcription factor activity, RNA polymerase II-specific; RNA polymerase II activating transcription factor binding	*HIVEP2*
*SppL*	Signal peptide peptidase-like	Aspartic endopeptidase activity, intramembrane cleaving	*SPPL3*
*Src42A*	Src oncogene at 42A	ATP binding; non-membrane spanning protein tyrosine kinase activity; signaling receptor binding; protein tyrosine kinase activity	*FRK*
*Src64B*	Src oncogene at 64B	ATP binding; non-membrane spanning protein tyrosine kinase activity; signaling receptor binding	*SRC FYN*
*Syn1*	Syntrophin-like 1	Structural constituent of muscle; cytoskeletal protein binding	*SNTB1*
*Ten-m*	Tenascin major	Identical protein binding; protein binding; filamin binding; protein homodimerization activity; protein heterodimerization activity	*TENM1*
			*TENM2*
*Thor*	Thor	Eukaryotic initiation factor 4E binding	*EIF4EBP1*
			*EIF4EBP2*
*Tl*	Toll	Protein binding; cytokine binding; transmembrane signaling receptor activity; cytokine receptor activity; TIR domain binding	*TLR1*
			*TLR2*
			*TLR3*
			*TLR4*
			*TLR6*
			*TLR7*
			*TLR9*
			*TLR10*
*Tsp96F*	Tetraspanin 96F	–	*CD81*
*Usp47*	Ubiquitin specific protease 47	Cysteine-type endopeptidase activity; thiol-dependent ubiquitin-specific protease activity	*USP47*
*wde*	Windei	–	*ATF7IP*

### Transcriptomic Analysis Identifies 908 Differentially Expressed Genes Upon Human Tau Overexpression in *Drosophila* Primary Neuronal Cultures

Independently from these genetic results, we developed *Drosophila* primary neuronal cultures overexpressing or not the wild-type human Tau protein. After 3 days of culture, the cultures displayed well-developed, ramified, and polarized nerve cells (Supplementary Figure S2A). The human Tau protein reached its steady-state level (data not shown) and was located in the soma and in all the neural processes in our cellular model (Supplementary Figure S2A). As expected (Li et al., 2016; Bolós et al., 2017), Tau overexpression led to a significant reduction in the average length of the neural extensions compared to control primary cultures without Tau (Supplementary Figures S2B,C) [*p*-value = 0.003, “control” mean = 59.160 ± 5.142 μm, “Tau” mean = 30.884 ± 2.416 μm, 95% CI (14.185; 42.368)]. In order to determine gene expression dysregulations induced by Tau overexpression in these primary neuronal cultures, we carried out a transcriptional analysis using 2-color microarrays. A total of 908 genes were found differentially expressed (*p*-value logRatio < 0.01, absolute fold changes > 1.5) upon Tau overexpression, 568 being upregulated and 340 being down-regulated (Supplementary Tables S3, S4). It should be noted that expression analyses reveal only a limited number of “causative” genes and that a large set of the differentially expressed genes might be downstream of these effectors or due to secondary effects.

### Combined Genetic and Transcriptomic Analyses Allow the Identification of 18 New Genetic Modifiers of Tau Toxicity

**FIGURE 1:**
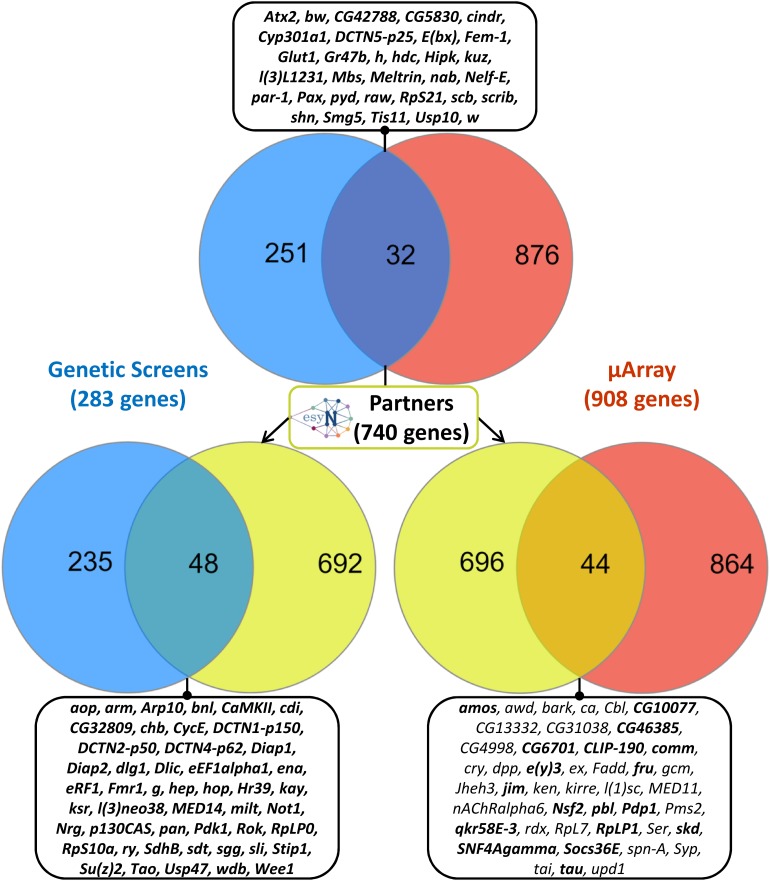
Unbiased combinatory approach crossing genetic and transcriptomic datasets. On the top, Venn diagram showing logical relations between the set of genetic interactors of human Tau identified in *Drosophila* (“Genetic screens,” in blue) and the set of genes whose expression was dysregulated (fold change > 1.5) upon human Tau expression in *Drosophila* primary nerve cells (“μArray,” in red). The intersection of these two datasets encompassed 32 genes, which are listed in the corresponding stamp. On the middle, identification of 740 physical and genetic partners of these 32 genes using the easy networks database (esyN). On bottom left, Venn diagram showing logical relations between the set composed of these 740 protein partners (in yellow) and the set of genetic interactors of human Tau (in blue). The intersection of these two datasets is composed of 48 genes, which are listed in the corresponding stamp. On bottom right, Venn diagram showing logical relations between the set composed of the 740 protein partners (in yellow) and the set of genes dysregulated upon human Tau overexpression in primary nerve cells (in red). The intersection of these two datasets encompassed 44 genes, which are listed in the corresponding stamp. In stamps, genetic interactors validated experimentally are indicated in bold.

Interestingly, the intersection of the list of the 283 genetic modifiers of Tau toxicity with the list of the 908 differentially expressed genes upon Tau expression revealed 32 genes dysregulated downstream of Tau expression *in vitro* and whose misexpression *in vivo* modulates Tau-induced toxicity (Figure 1). These 32 genes represented 3.5% of the 908 differentially expressed genes, which was only slightly above the background level of 2% (283/∼15,500 total fly genes) if both categories (genetic modifiers and differentially expressed genes) were random. However, a proportion comparaison test (reference: 2%) revealed that the difference was statistically significant [*p*-value = 0.001, 95% CI (0.025–0.049)] and that the Tau toxicity modifiers were over-represented among the 908 differentially expressed genes. Therefore, we reasoned that the dysregulation of the expression of each of these 32 genes could have functional consequences on the biology of their physical and genetic partners, and therefore that some of these partners could act as genetic modifiers of Tau toxicity. Using the easy networks database (esyN), we identified 740 physical and/or genetic interaction partners of these 32 genes. Interestingly, 48 of these 740 partners (6.5%) were part of the list of the 283 genetic modifiers of Tau toxicity identified using the REP, which validates our approach (Figure 1). Again, a proportion comparaison test (reference: 2%) showed a significant enrichment of Tau genetic interactors among these 740 partners [*p*-value was < 2.2e-16, 95% CI (0.049–0.085)]. On the other hand, 44 of these 740 partners were found dysregulated upon Tau expression *in vitro* (Figure 1), but not identified so far as genetic modulators of Tau toxicity. In order to determine whether these 44 factors can modulate Tau-induced neurodegeneration in *Drosophila*, *GMR-Gal4* > *hTau*^*WT*^ flies were crossbred with mutant lines at these loci, and the F1 generation was screened for changes in the Tau-dependent REP. We found that genetic manipulations of 18 of them robustly modified Tau-induced neurodegeneration in *Drosophila* (Table 2, Supplementary Figure S1, and Supplementary Table S2). We verified that none of the mutant alleles used produced offspring with REP, after being crossed to the *GMR-Gal4* driver line alone (data not shown). Among these 18 additional genes, we identified three structural components of the cytoskeleton organization, including the *Drosophila* ortholog of the human *Tau* gene, and numerous factors involved in gene expression and chromatin regulation.

**TABLE 2:** Identification of 18 supplementary genetic modifiers of Tau toxicity in *Drosophila.*

**Gene symbol**	**Gene name**	**Molecular function**	**Human orthologs**
*amos*	Absent MD neurons and olfactory sensilla	Protein heterodimerization activity; RNA polymerase II regulatory region DNA binding	*ATOH1*
*CG6701*	–	RNA binding; ATP-dependent 5′-3′ RNA helicase activity	*MOV10*
*CG10077*	–	ATP binding; RNA helicase activity; nucleic acid binding	*DDX5*
*CG46385*	–	RNA adenylyltransferase activity	*–*
*CLIP-190*	Cytoplasmic linker protein 190	Actin binding; microtubule binding; myosin VI heavy chain binding; protein binding; microtubule plus-end binding	*CLIP1*
			*CLIP2*
*comm*	Commissureless	WW domain binding; protein binding; Roundabout binding	*PRRG4*
*e(y)3*	Enhancer of yellow 3	Histone acetyltransferase activity; chromatin binding; histone binding	*PHF10*
*fru*	Fruitless	Nucleic acid binding; DNA-binding transcription factor activity	*ZBTB1*
			*ZBTB24*
			*ZBTB39*
			*ZBTB45*
*jim*	Jim	Nucleic acid binding	*ZNF133*
			*ZNF343*
			*ZNF460*
			*ZNF708*
*Nsf2*	N-ethylmaleimide-sensitive factor 2	ATP binding; ATPase activity	*NSF*
*pbl*	Pebble	GTPase activator activity; Rho GTPase binding; Rho guanyl-nucleotide exchange factor activity; phosphatidylinositol phosphate binding; Rac guanyl-nucleotide exchange factor activity; semaphorin receptor binding	*ECT2*
*Pdp1*	PAR-domain protein 1	Sequence-specific DNA binding; RNA polymerase II regulatory region sequence-specific DNA binding; DNA-binding transcription activator activity, RNA polymerase II-specific; DNA-binding transcription factor activity	*HLF*
*qkr58E-3*	Quaking related 58E-3	RNA binding	*KHDRBS1*
			*KHDRBS2*
			*KHDRBS3*
*RpLP1*	Ribosomal Protein LP1	Protein kinase activator activity; structural constituent of ribosome; ribonucleoprotein complex binding	*RPLP1*
*skd*	Skuld	Transcription coregulator activity; protein binding	*MED13*
*SNF4Agamma*	SNF4/AMP-activated protein kinase gamma subunit	Adenyl ribonucleotide binding; protein kinase binding	*PRKAG1*
			*PRKAG2*
*Socs36E*	Suppressor of cytokine signaling at 36E	Cytokine receptor binding; sevenless binding; 1-phosphatidylinositol-3-kinase regulator activity	*SOCS5*
*tau*	Tau	Microtubule binding	*MAPT*

To summarize all these data, the reevaluation of our genetic screen allowed us to identify 59 novel Tau genetic modulators (section “A genetic Screen Identifies 59 Novel Modifiers of Tau Toxicity in *Drosophila*”). Then, combining genetic analyses and transcriptomic analyses, we identified 18 additional Tau genetic interactors (section “Combined Genetic and Transcriptomic Analyses Allow the Identification of 18 New Genetic Modifiers of Tau Toxicity”). Added to the 224 genetic interactors identified in previous studies, these new data bring to 301 the number of genetic modifiers of human Tau-induced toxicity identified so far in flies using REP as read-out (Supplementary Table S5). This combined strategy also allowed us to show that 50 (32 and 18) of these genetic modulators were dysregulated upon Tau expression *in vitro* and that their misexpression *in vivo* modulates Tau induced toxicity, suggesting that they may participate in Tau-driven toxicity *in vivo* in *Drosophila* (Figure 1).

### Network Analysis Highlights Non-random Interconnectivity Between the Genetic Modifiers of Tau Toxicity

**FIGURE 2:**
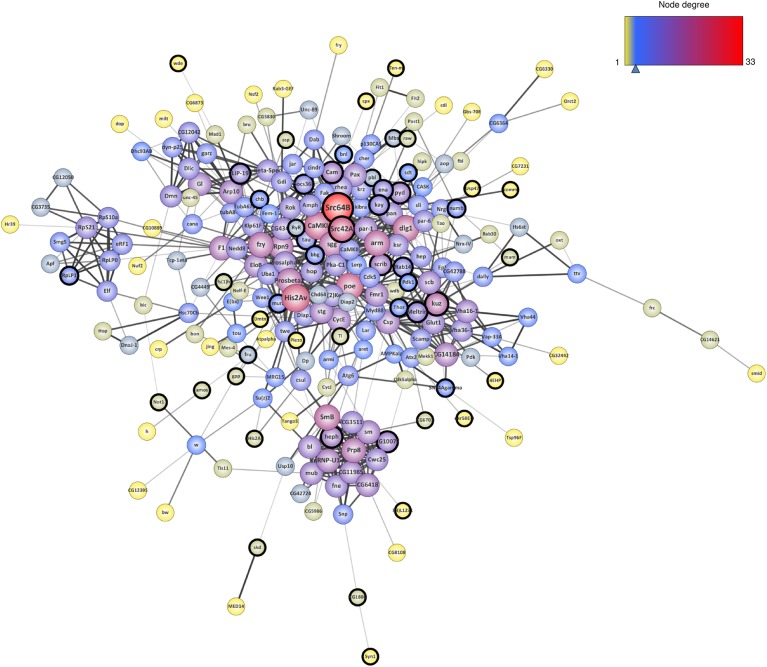
Interaction network resulting from the genetic modifiers of Tau toxicity so far identified in flies. The nodes corresponding to the newly identified genes are surrounded with a black halo. The size of the nodes is positively correlated with the degree of the genes (number of connections) and the color of the nodes follows a degree-defined continuous color mapping. The tick under the color mapping corresponds to the median degree. The thickness of the edges indicates the level of confidence prediction of the interaction. The confidence cut-on for showing interaction links has been set to 0.5 and the disconnected nodes in the network have been hidden.

To analyze the biological connections between these 301 genetic Tau-modifiers, we constructed an interaction network using the STRING (Search Tool for the Retrieval of Interacting Genes/Proteins) database. We restricted the analysis to the following categories of interactions: protein–protein interactions (PPI) documented by co-immunoprecipitation or yeast two-hybrid, functional interactions documented by data gathered from curated databases and text-mining that detects co-occurrence of gene names in literature. Furthermore, we considered only interactions with confidence scores over 0.5. This led to a connected network of 229 genes (Figure 2). Note that 72 of the 301 genetic Tau-modifiers identified so far were not found in this network. This could be due to the fact that they are less studied genes, or they could interact with Tau independently of the other genetic interactors identified. Using the whole *Drosophila* genome as background, we found that the network enrichment *p*-value was <1.0e-16, meaning that this connected network has significantly more interactions than expected at random, and that the genetic modifiers have more interactions among themselves than what would be expected for a random set of proteins of similar size. Such enrichment also indicates that these genetic modifiers are, at least partially, biologically connected.

**FIGURE 3:**
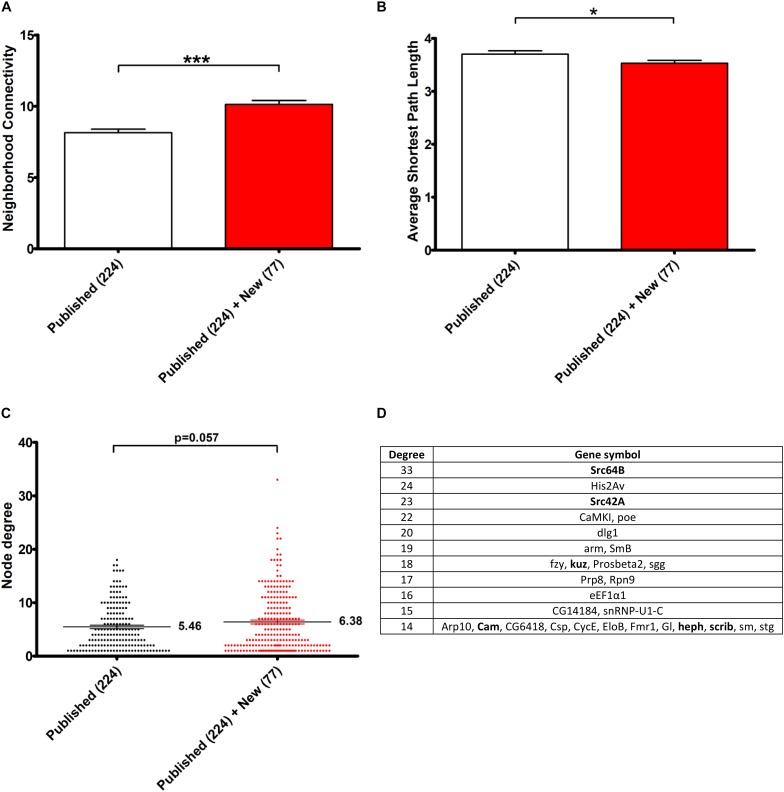
Contribution of the 77 genetic Tau-modulators in the construction of a connected Network. **(A,B)** Histograms representing, respectively, the neighborhood connectivity (average connectivity of all neighbors of each node) **(A)** and the average shortest path length (average number of steps along the shortest paths for all possible pairs of network nodes) **(B)** of the network presented in Figure 2. The bars and error bars correspond to the means ± SEM. “Published”: genetic interactors previously identified before this study, “New,” genetic interactors identified in this study. *p < 0.05; ***p < 0.001; unpaired Welch *t*-test. **(C)** The scatter plot representing the node degree values of the genes. The lines and error bars correspond to the means ± SEM. The means are indicated next to each dataset and *p*-values were calculated using the unpaired Welch *t*-test. **(D)** The table lists the top list of genes with highest node degrees (Degree, 1st column) corresponding to the dataset “Published” + “New.” The genes in bold correspond to genetic interactors of Tau identified in this study.

In order to estimate the contribution of the 77 novel genetic modifiers identified in this study in the construction of this connected network, we performed a similar network analysis considering only the 224 interactors identified in previous studies. Interestingly, the addition of these 77 novel genetic modifiers resulted in a greater neighborhood connectivity (average connectivity of all neighbors of each node) [*p*-value = 1.944E-7, “published” mean = 8.147 ± 0,243, “published + new” mean = 10.122 ± 0.283, 95% CI (−2.708; −1.241)] and a weaker average shortest path length (distance between two connected genes) [*p*-value = 0.040, “published” mean = 3.701 ± 0.062, “published + new” mean = 3.531 ± 0.054, 95% CI (0.008; 0.333)] (Figures 3A,B), indicating that the addition of the newly identified genes filled missing links. On the other hand, though the addition of these 77 genes did not significantly impact the average node degree (average number of connections per gene) [*p*-value = 0.057, “published” mean = 5.461 ± 0.322, “published + new” mean = 6.385 ± 0.362, 95% CI (−1.877; 0.028)], it interestingly brought up some key hubs with very high connectivities, namely *Src64B, Src42A, kuz, heph, scrib*, and *Cam* (Figures 3C,D).

### Network Clustering Highlights Biological Processes Related to Tau Toxicity

**FIGURE 4:**
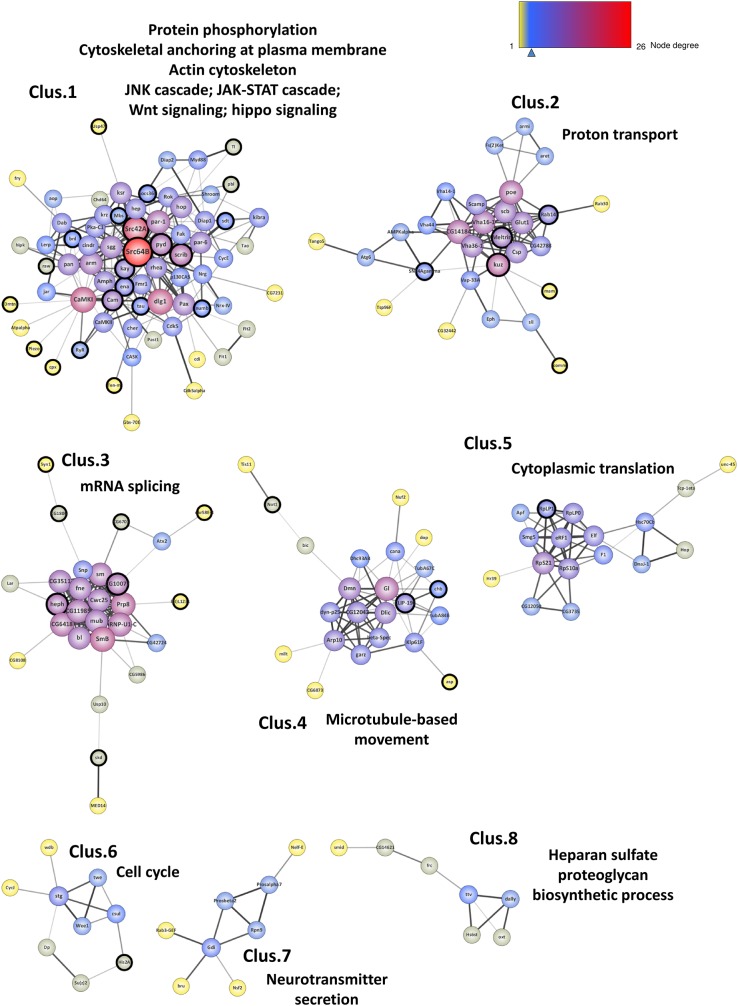
Network clustering. Representation of the 8 subgroups (Clus.1 to Clus.8) resulting from the network clustering analysis. The nodes corresponding to the new genes identified in this study are surrounded with a black halo. The size of the nodes is positively correlated with the degree of the genes (number of connections) and the color of the nodes follows a degree-defined continuous color mapping. The tick under the color mapping corresponds to the median degree. The thickness of the edges indicates the level of confidence prediction of the interaction. The confidence cut-on for showing interaction links has been set to 0.5 and the disconnected nodes in the network have been hidden.

Using network-clustering algorithms to detect densely connected subgroups in the network, we identified 8 main modules (Figure 4). Downstream analysis of these sub-networks using DAVID bioinformatics resources revealed highly significant functional enrichments (Supplementary Table S6). The sub-network 1 is highly enriched in genes involved in protein phosphorylation, actin organization and signal transduction. Sub-network 2 genes are robustly associated with proton transport. The module 4 is enriched in genes involved in microtubule-based movement, and the cluster 7 with hits associated with neurotransmitter secretion and vesicle dynamics. Tagging modules with GO terms also outlined cell cycle activities (module 6), RNA metabolism (clusters 3 and 5) and Heparan sulfate proteoglycan (HSPGs) biosynthesis (module 8). Interestingly, many of these processes are in agreement with Tau physiological and pathological functions (Hannan et al., 2016; Sotiropoulos et al., 2017; Zhou et al., 2017; Maïza et al., 2018).

### Human Orthologs of the Genetic Modifiers of Tau Toxicity Are Less Tolerant Than Average to Haploinsufficiency/Inactivation

**FIGURE 5:**
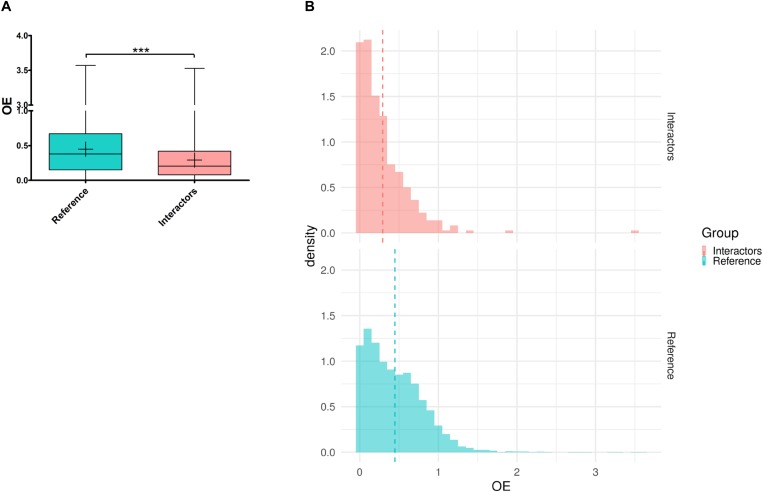
Tolerance to haploinsufficiency/inactivation analysis of the human orthologs of the genetic modifiers of Tau toxicity. **(A)** Box plot representing the observed/expected ratios of predicted loss-of-function (pLoF) variation (OE) for two datasets: “Reference” corresponding to all the human genes with *Drosophila* orthologs (in blue), and “Interactors” corresponding to the human orthologs of the genetic interactors of Tau identified in *Drosophila* (in red). Whiskers represent min and max values. “+” represent the means (***p < 0.001; unpaired Welch *t*-test). **(B)** Density histogram representing the OE distribution for the “Reference” (in blue) and “Interactors” (in red) datasets (bins of 0.1). The area of each “Density” bar equals the relative frequency of the corresponding class, and the area of the entire histogram equals 1. The dash lines represent the means.

Based on the analysis of 125,748 exomes and 15,708 human genomes, the Genome Aggregation Database (gnomAD) provides for all human genes the ratio of observed over expected loss-of-function variations (LoF o/e). This ratio constitutes a continuous constraint metric describing the spectrum of tolerance to loss-of-function for each protein-coding gene. It has been observed that essential genes for human cell viability are far more intolerant to haploinsufficiency/inactivation than non-essential genes that are more likely to be unconstrained (Lek et al., 2016). Moreover, genes responsible for mendelian diseases are significantly more intolerant to functional genetic variation than genes that do not cause any known disease (Petrovski et al., 2013). Interestingly, the intolerance of a gene correlates with its degree of connection in protein interaction network (Karczewski et al., 2019). We have shown above that the genetic modifiers of Tau toxicity identified in *Drosophila* constituted a non-random interaction network and were likely connected as a functional biological group. To study the tolerance to inactivation/loss of function of the 301 Tau genetic modifiers identified so far, we first looked for their human orthologs using the DRSC Integrative Ortholog Prediction Tool (DIOPT). Only the best matches were considered when there was more than one match per input *Drosophila* gene. No human orthologs were reported for 15 of them. For the remaining 286 genetic modifiers, we identified 364 human orthologs, consistently with the fly-human one-to-many relationships due to whole genome duplications during evolution (Supplementary Table S5). Then, we computed the mean LoF o/e for the human orthologs of the genetic interactors and for a reference set of genes corresponding to all human genes with fly orthologs, considering that conserved genes from fly-to-human are naturally more constraint than unconserved ones. The mean LoF o/e for the orthologs of the genetic modifiers of Tau toxicity was significantly lower than the mean LoF o/e of the rest of the genome [*p*-value = 2.2E-16, “reference” mean = 0.448 ± 0.004, “Tau interactors” mean = 0.292 ± 0.017, 95% CI (0.121; 0.190)] (Figure 5A). Looking at the LoF o/e distribution for the two datasets, we found a higher density of weak LoF o/e values for the human orthologs of the genetic modifiers of Tau toxicity compared to reference genes (Figure 5B). These data indicated that the human orthologs corresponding to the Tau genetic modifiers identified in flies were more intolerant to inactivation/loss-of-function than average. Thus, they are likely critical for cellular functions, and possibly disease-causing.

### Human Orthologs of Genetic Modifiers of Tau Toxicity Involved in Neurological Disorders

Next, we used DIOPT-Diseases and Traits (DIOPT-DIST) to determine if these interactors were associated with human diseases. Among the 364 human orthologs of genes coding for Tau interactors, 87 are involved in the genetic determinism of human diseases according to the OMIM database, which is consistent with their intolerance to inactivation. Among them, 38 are involved in the determinism of neurodevelopmental or neurodegenerative diseases (Table 3). In most of these diseases, no Tau pathology has been described to date. However, 2 of these genes are risk factors for AD, a condition in which Tau pathology is a well-recognized feature of the disease. First, the *BIN1* locus is a firmly established susceptibility AD locus, with a frequent variant located outside the coding sequence conferring AD risk (Lambert et al., 2013). *BIN1* codes for Amphiphysin2, a protein involved in synaptic vesicles endocytosis, which physically interacts with Tau (Chapuis et al., 2013; Lasorsa et al., 2018). In AD, Tau pathology propagates within synaptically connected neurons (de Calignon et al., 2012; Liu et al., 2012) and *in vitro* experiments have shown that *BIN 1* silencing increases Tau propagation by promoting aggregate internalization (Calafate et al., 2016). Second, a frequent variant of *ADAM10* is now a recognized AD protective factor (Kunkle et al., 2019). *ADAM10* codes for a secretase involved in the non-amyloidogenic processing of the Amyloid precursor protein. In addition to this putative pathophysiological mechanism, we now report for the first time a link between *ADAM10* down-regulation and Tau-induced toxicity.

**TABLE 3:** Orthologs of genetic interactors are associated with human diseases.

**Fly gene symbol**	**Human gene symbol**	**Disease/Trait**	**Inheritance**
*alphaTub67C*	*TUBA4A*	Amyotrophic lateral sclerosis 22 with or without frontotemoral dementia, 616208	AD
*Amph*	*BIN1*	Alzheimer disease, susceptibility to	Mu
*arm*	*CTNNB1*	Mental retardation, autosomal dominant 19, 615075	AD
*Arv1*	*ARV1*	Epileptic encephalopathy, early infantile, 38, 617020	AR
*asp*	*ASPM*	Microcephaly 5, primary, autosomal recessive, 608716	AR
*Atpalpha*	*ATP1A3*	Alternating hemiplegia of childhood 2, 614820	AD
		CAPOS syndrome, 601338	AD
		Dystonia-12, 128235	AD
*Atx2*	*ATXN2*	Amyotrophic lateral sclerosis, susceptibility to, 13, 183090	AD
		Spinocerebellar ataxia 2, 183090	AD
		Parkinson disease, susceptibility to, 168600	AD, Mu
*beta-Spec*	*SPTBN2*	Spinocerebellar ataxia 5, 600224	AD
		Spinocerebellar ataxia, autosomal recessive 14, 615386	AR
*brun*	*TRAPPC9*	Mental retardation, autosomal recessive 13, 613192	AR
*cana*	*CENPE*	Microcephaly 13, primary, autosomal recessive, 616051	AR
*CASK*	*CASK*	Mental retardation and microcephaly with pontine and cerebellar hypoplasia, 300749	XLD
*Cdk5*	*CDK5*	Lissencephaly 7 with cerebellar hypoplasia, 616342	AR
*CG10927*	*ADAT3*	Mental retardation, autosomal recessive 36, 615286	AR
*CG17327*	*PTRH2*	Infantile-onset multisystem neurologic, endocrine, and pancreatic disease, 616263	AR
*CG42788*	*FRMPD4*	Mental retardation, X-linked 104, 300983	XLD
*Csp*	*DNAJC5*	Ceroid lipofuscinosis, neuronal, 4, Parry type, 162350	AD
*Cyp301a1*	*CYP27A1*	Cerebrotendinous xanthomatosis, 213700	AR
*DCTN1-p150*	*DCTN1*	Neuropathy, distal hereditary motor, type VIIB, 607641	AD
		Perry syndrome, 168605	AD
		Amyotrophic lateral sclerosis, susceptibility to, 105400	AR, AD
*Ef1alpha48D*	*EEF1A2*	Epileptic encephalopathy, early infantile, 33, 616409	AD
		Mental retardation, autosomal dominant 38, 616393	AD
*Fmr1*	*FMR1*	Fragile X syndrome, 300624	XLD
		Fragile X tremor/ataxia syndrome, 300623	XLD
*fru*	*ZBTB24*	Immunodeficiency-centromeric instability-facial anomalies syndrome-2, 614069	AR
*g*	*AP3D1*	Hermansky-Pudlak syndrome 10, 617050	AR
*Gabat*	*ABAT*	GABA-transaminase deficiency, 613163	AR
*Gdi*	*GDI1*	Mental retardation, X-linked 41, 300849	XLD
*HisRS*	*HARS*	Charcot-Marie-Tooth disease, axonal, type 2W, 616625	AD
		Usher syndrome type 3B, 614504	AR
*HnRNP-K*	*HNRNPK*	Au-Kline syndrome, 616580	AD
*Klp61F*	*KIF11*	Microcephaly with or without chorioretinopathy, lymphedema, or mental retardation, 152950	AD
*kuz*	*ADAM10*	{Alzheimer disease 18, susceptibility to}, 615590	Mu
*Nrx-IV*	*CNTNAP2*	Cortical dysplasia-focal epilepsy syndrome, 610042	AR
		Pitt-Hopkins like syndrome 1, 610042	AR
*Oct-TyrR*	*ADRA2B*	Epilepsy, myoclonic, familial adult, 2, 607876	AD
*oxt*	*XYLT1*	Desbuquois dysplasia 2, 615777	AR
*Pdk*	*PDK3*	Charcot-Marie-Tooth disease, X-linked dominant, 6, 300905	XLD
*Piezo*	*PIEZO2*	Marden-Walker syndrome, 248700	AD
		Arthrogryposis, distal, type 3, 114300	AD
		Arthrogryposis, distal, type 5, 108145	AD
		Arthrogryposis, distal, with impaired proprioception and touch, 617146	AR
*shn*	*HIVEP2*	Mental retardation, autosomal dominant 43, 616977	AD
*SmB*	*SNRPB*	Cerebrocostomandibular syndrome, 117650	AD
*Uba1*	*UBA1*	Spinal muscular atrophy, X-linked 2, infantile, 301830	XLR
*Vap33*	*VAPB*	Amyotrophic lateral sclerosis 8, 608627	AD
		Spinal muscular atrophy, late-onset, Finkel type, 182980	AD
*vnc*	*NAA10*	Ogden syndrome, 300855	XLR, XLD
		Microphthalmia, syndromic 1, 309800	XL

*AD, autosomal dominant; AR, autosomal recessive; Mu, multifactorial; XL, X-linked; XLD, X-linked dominant; XLR, X-linked recessive.*

## Discussion

Deciphering the pathophysiological mechanisms that lead from the alteration of Tau biology to neuronal death in tauopathies depends on the identification of Tau cellular partners. Since 2003, *Drosophila* models of tauopathies have been widely used to identify genetic modifiers of Tau toxicity *in vivo* (Hannan et al., 2016). In this study, we took advantage of a fly model overexpressing the wild-type form of human Tau protein to identify new modulators of human Tau-induced toxicity. This *Drosophila* model recapitulates some key pathological features of human tauopathies, including neuronal loss, neurodegeneration, premature death, and accumulation of abnormally phosphorylated forms of Tau (Wittmann et al., 2001). We showed that, when expressed in *Drosophila*, human Tau protein binds very weakly to microtubules and is mostly recovered as soluble cytosolic hyperphosphorylated species (Feuillette et al., 2010). The accumulation of these species correlates with human Tau-mediated neurodegeneration in flies (Feuillette et al., 2010).

In this report, combined genetic and transcriptomic analyses allowed the identification of 77 new genetic interactors, bringing to 301 the number of genetic modifiers of human Tau-induced toxicity identified so far in *Drosophila.* The study of the biological connections between these 301 genetic Tau-modifiers led to a connected network of 229 genes with high biological relevance. Interestingly, the addition of these new 77 genetic Tau-modifiers resulted in a greater neighborhood connectivity, a weaker average shortest path length, and brought up key hubs with high connectivities.

We identified many factors involved in cytoskeleton organization, neurotransmitter secretion and vesicle dynamics, gene expression, chromatin remodeling, RNA metabolism, as well as kinases, metalloproteases ion channels and scaffolding proteins. These interactors have already been related to Tau physiological and pathological functions. So, most likely the REP phenotype observed in flies results from several mechanisms of Tau toxicity, linked to the various Tau functions.

Interestingly, two key hubs identified in this study, *Src64B* and *Src42A*, are the *Drosophila* orthologs of *FYN* and *SRC*, two members of the Src family non-receptor tyrosine kinase. A link between Tau and Src tyrosine kinases has already been highlighted in many studies. Both are expressed in neurons of mammalian brains, and are particularly abundant at synaptic sites. Tau can physically interact with Fyn and Src, *via* its proline-rich region (Lee et al., 1998), and Fyn can phosphorylate Tau at tyrosine-18 (Bhaskar et al., 2005; Miyamoto et al., 2017). Furthermore, dendritic Tau was found to function as an intracellular shuttle for transporting Fyn to the post-synaptic density (PSD) domain, where it regulates NMDA receptor function through phosphorylation (Ittner et al., 2010). A recent study showed that Tau controls the nanoscale organization of Fyn in dendrites (Padmanabhan et al., 2019). In this study, we found that loss-of-function of *Src64B* and *Src42A* in Tau-expressing cells enhanced Tau toxicity. Importantly, in our study, as in all those included in the joined-analysis, human Tau proteins were overexpressed in retinal cells using the *GMR-Gal4* driver line, thus targeting only presynaptic terminals of photoreceptors. Therefore, our data strongly suggest that Fyn and Src proteins could also modulate Tau toxicity in presynaptic compartment. Under pathological conditions, Tau localizes with both pre- and post-synaptic terminals (Tai et al., 2012, 2014; Zhou et al., 2017; McInnes et al., 2018), suggesting that Tau function at the presynapse may also contribute to disease pathogenesis. In fly and rat neurons, it has been shown that mislocalized Tau in presynaptic terminals binds to synaptic vesicles via its N-terminal domain and simultaneously promotes presynaptic actin polymerization to crosslink vesicles, restricting their mobilization, their release rate, and thus lowering neurotransmission (Zhou et al., 2017). Several reports highlight that Src tyrosine kinases might also influence the mobility of synaptic vesicles and neurotransmitter release (Meijer et al., 2018). Thus, Src tyrosine kinases might act on Tau toxicity properties either directly by modulating its phosphorylation status or indirectly by regulating signaling pathways involved in Tau toxicity. It might also be linked to Src tyrosine kinases involvement in cytoskeleton-dependent process that maintains synaptic function. Further studies will be necessary to elucidate the underlying mechanisms of Tau/Src tyrosine kinases genetic interactions.

Consistent with presynaptic Tau-induced toxicity, we also identified several genes known to be involved in neurotransmitter release from synaptic vesicles, including *complexin* (*cpx*), *Rab14*, *Ryanodine receptor* (*RyR*), and *scribble* (*scrib*). Neurotransmitters are released by calcium-triggered exocytosis of membrane-docked synaptic vesicles and recycled by compensatory endocytosis. The refilling of newly formed synaptic vesicles with neurotransmitters is driven by a proton-electrochemical gradient generated by a vacuolar H + -ATPase. The *cpx* gene encodes a presynaptic cytosolic protein that regulates SNARE complex assembly and function. It has both positive and negative roles in synaptic transmission, serving as the synaptic vesicle fusion clamp and as an activator of evoked release. Cpx cooperates with Bruchpilot (human ortholog: ERC2) to promote synaptic vesicles recruitment to the active zone cytomatrix (Scholz et al., 2019). *RyR* encodes an intracellular calcium-release channel localized on presynaptic endoplasmic reticulum membranes. It regulates the release of intracellular calcium stores, and therefore has a key role in vesicular mobilization and release of transmitters and neuropeptides (Levitan, 2008). Lastly, Rab14 is an endocytotic Rab GTPase enriched on the surface of purified synaptic vesicles membranes (Pavlos and Jahn, 2011). Rab14 has been implicated in clathrin-coated trafficking and recycling pathways. Interestingly, several vacuolar proton pumps (Vha14-1/ATP6V1F, Vha16-1/ATP6V0C, Vha36-1/ATP6V1D, and Vha44/ATP6V1C1) were also previously identified as Tau-genetic modifiers. Polymerization of presynaptic actin is another key element for synaptic vesicles clustering and release from the active zones. We also identified *scrib*, a master scaffolding protein that acts in apico-basal polarity, adhesion, proliferation, presynaptic architecture, and synaptogenesis (Bonello and Peifer, 2019). It is localized in the nervous system both in invertebrate and vertebrate animals, and particularly enriched at synapses (Moreau et al., 2010). Fly *scrib* loss-of-function mutant show abnormally high synaptic vesicles density in the reserve vesicle pool and a decrease of the number of actives zones (Roche et al., 2002). Likewise, *scrib* knockdown alters synaptic vesicle clustering in mice (Sun et al., 2009). Several studies led to a model in which Scrib protein interacts with adhesion complexes (Nrx/Nrg and N-cad/β-cat), facilitating localized Rac activity and F-actin polymerization. Thus, numerous factors involved at different steps in the synaptic vesicle cycle act as genetic modifiers of Tau toxicity in flies. Additional studies will be needed to clarify how these factors interact with Tau at the presynaptic nerve terminal.

Cytoskeleton regulator elements are another category that is very well represented among our new genetic interactors. Among these, we identified *enabled (ena)/ENAH* that acts as processive actin polymerase, stimulating actin addition at the barbed end of actin filaments. Regarding the microtubule cytoskeleton, we detected genetic interactions with *chromosome bows (chb), CLIP-190*, and *Tau*. The *Drosophila chb and CLIP-190* genes, and their human orthologs *CLASP1/CLASP2* and *CLIP1/CLIP2*, respectively, encode microtubule plus-end tracking proteins (+TIP) that preferentially associate with the growing plus-ends of microtubules, and control microtubule end dynamics and anchorage to other structures, including actin filaments (van de Willige et al., 2016). Regarding *Drosophila Tau (dTau)*, as its human counterpart, it binds the lattice of microtubules (Feuillette et al., 2010). Recent reports have demonstrated that Tau controls end-binding proteins (EBs) tracking at microtubules ends (Ramirez-Rios et al., 2017). The EBs are members of the protein family of + TIP proteins (Sayas et al., 2015). Beyond its microtubule-stabilizing properties, Tau is also a regulator of actin both *in vitro* and *in vivo* (Frandemiche et al., 2014; Mohan and John, 2015). Tau acts as a direct linker of dynamic microtubules and actin filaments, enabling the co-organization of the two networks in purified cell-free systems (Elie et al., 2015). Therefore, these three factors connect the actin and microtubule cytoskeleton. It is becoming increasingly clear that the two cytoskeletal systems often work together in core cellular processes, including axon organization, neurites formation, synaptic function, cell migration, cell polarity and cell division (Dogterom and Koenderink, 2019). If the presence and the function of microtubules at presynaptic terminals, and their interaction with actin filaments are only partially resolved (Bodaleo and Gonzalez-Billault, 2016), the identification of these three genetic modulators of Tau toxicity in our experimental system suggest that alteration of the actin-microtubule crosstalk in presynaptic terminal (due to mislocalization of pathological Tau) might contribute to Tau toxicity. Interestingly, we found that both down-regulation and over-expression of dTau enhanced human Tau-induced neurodegeneration. It has been shown that expression of highly phosphorylated human Tau functionally compromised the microtubule-binding ability of the endogenous dTau (Cowan et al., 2010). Thus, both excessive binding of Tau on microtubules and/or actin filaments, as well as a default binding of the protein due to a decrease of its amount might interfere with cytoskeleton elements stability, organization, dynamics, and cross-talk.

## Conclusion

To conclude, as emphasized in a recent review (Ittner and Ittner, 2018), providing a comprehensive map of Tau interactors in synaptic compartments is a key step in order to understand the pathological mechanisms involved in tauopathies. This current work is in line with this objective. Combining genetic and transcriptomic analyses in *Drosophila*, we identified 77 new genes, bringing to 301 the number of human Tau-genetic modifiers identified so far in flies, 229 of which constituting a connected network. Network analysis showed that the addition of these 77 new modulators strengthened the network structure, increased the intergenic connectivity and brought up key hubs with high connectivities. Our new data also supports the importance of the presynaptic compartment in mediating Tau toxicity. These cellular partners of Tau should allow a better understanding of the underlying mechanisms of Tau toxicity, notably in presynaptic terminals, and could constitute new therapeutic targets. Further studies will be necessary to elucidate how these factors interact with Tau. In addition, it will be also essential to validate the relevance of the genetic interactions identified in *Drosophila* toward the human pathologies using mammalian cellular and animal models.

## Data Availability Statement

The datasets generated for this study can be found in the ArrayExpress database (https://www.ebi.ac.uk/arrayexpress/) under the accession number E-MTAB-8712.

## Author Contributions

SF and ML: conceptualization. SF: methodology, investigation, and resources. SF and CC: formal analysis and visualization. SF, DC, and ML: writing – original draft. DC and ML: supervision. ML, DC, and TF: funding acquisition.

## Conflict of Interest

The authors declare that the research was conducted in the absence of any commercial or financial relationships that could be construed as a potential conflict of interest.

## Footnotes

1 https://fiji.sc

2 https://www.ebi.ac.uk/arrayexpress/

3 http://www.esyn.org

4 https://string-db.org

5 https://cytoscape.org

6 https://david.ncifcrf.gov

7 https://www.flyrnai.org/diopt

8 https://www.flyrnai.org/diopt-dist

9 https://gnomad.broadinstitute.org

## Abbreviations

AD: Alzheimer’s disease
LoF o/e: observed/expected ratio of loss-of-function variations.
