# Supplementary material for: A Connected Network of Interacting Proteins Is Involved in Human-Tau Toxicity in Drosophila
Source: Front Neurosci. 2020 Feb 11;14:68. doi: 10.3389/fnins.2020.00068 (PMC7026268; doi:10.3389/fnins.2020.00068)
Supplement: Supplementary file 1 [file Table_1.DOCX]

***Supplementary Material***

1. **Supplementary Figures Legends**

**Figure S1. Pictures depicting genetic interactions between human Tau and mutants alleles in *Drosophila* using REP as read-out.**

(A-D) Male fly eyes (for mutant alleles and transgenic constructs located on chromosome II and III). (E) Female fly eyes (for mutant alleles and transgenic constructs located on chromosome X). The exponent corresponds to the Bloomington *Drosophila* Stock Center (BDSC) stock number.

**Figure S2. *Drosophila* primary neurons expressing human Tau protein**

Primary neuronal monocultures derived from *elav-Gal4^GS^ > w^1118^* (control) or *elav-Gal4^GS^ > hTau^WT^* larval brains. Resulting primary neuronal cultures at day-in-vitro (DIV) 3 were analyzed by immunofluorescence microscopy. (A) Samples were visualized for human Tau protein (upper right corner; green on ‘merge’), for HRP, a neuron-specific membrane marker (bottom left corner; red on ‘Merge’), and counterstained with DAPI (upper left corner; blue on ‘Merge’). Scale bar: 25µm. (B) Example of extensions tracing (in green) and measures performed post-imaging on HRP immunostaining (in grey scale) on 3-days old control cultures or on cultures with neurons expressing human Tau. Scale bar: 25µm. (C) Histogram representing the average length of extensions *per* neuron in control primary cultures or in cultures with neurons expressing human Tau. Data are represented as means ± SEM. (n = 5; more than 900 neurons per culture type). (**: p < 0.01; unpaired Welch t-test).

1. **Supplementary Tables**

**Table S1.** Summary of Tau toxicity-related functional screenings *in Drosophila.* Table lists the genetic screenings and the functional validation of AD-related GWAs signals performed using the REP as read-out.

| **References** | **Screen type** | **Screen library** | **Number of Tau modifiers** | **Genes symbol** |
| --- | --- | --- | --- | --- |
| Jackson *et al.* (2002) | Candidate | Lines related to Wnt signaling | 6 | *arm, Diap1, Diap2, p35, pan, Sgg* |
| Shulman and feany (2003) | unbiased | 2276 EP strains | 27 | *Aop, Atx2, cdi, Cdk5, Cdk5alpha, CG10927, CG3735, CG7231, chb, cher, lncRNA:CR43650, dally, Diap1, Fem-1, Fmr1, fry, garz, Gbs-70E, hep, Orct2, par-1, Pka-C1, stg, Tao, twe, wdb, wun* |
| Karsten *et al*.(2006) | Candidate | Lines related to genes identified from mice microarray screen | 3 | *eEF1alpha1, Psa, Vap33* |
| Blard *et al*. (2007) | unbiased | 1250 EP strains | 30 | *Atpalpha, Bacc, CG10889, CG12395, CG42724, CG5830, CG7970, Chd64, cher, cic, Csp, DnaJ-1, Dp, E(bx), Hsc70Cb, jar, jing, melt, mub, Myd88, nab, Pax, poe, Ptp4E, Rad60, Shroom, Sodh-1, tou, Uba1, Vha44* |
| Shulman *et al*. (2011) | Candidate | Strains related to GWAs signals | 6 | *beta-Spec, dlg1, fne, Glut1, Hs6st, sli* |
| Ambegaokar and Jackson (2011) | unbiased | 920 P-lethal strains; 895 EY strains | 43 | *armi, Atg6, bw, CaMKI, cana, crp, csul, CycE, Dlic, eRF1, eRF3, frc, Fs(2)Ket, His2Av, HnRNP-K, Hr39, Klp61F, ksr, l(3)j11B2, l(3)j6A6, l(3)L0499, l(3)L6332, mei-9, Mekk1, Mi-2, milt, NC2alpha, Nrg, par-1, Past1, RpLP0, RpS21, ry, SdhB, sgg, sm, smid, Stip1, Tango5, Tis11, unc-45, Vha14-1, w* |
| Chapuis *et al*. (2013) | Candidate | Strains related to GWAs signals | 1 | *Amph* |
| Shulman *et al*. (2014) | Candidate | Strains related to GWAs signals | 9 | *bru1, cindr, dop, Fit1, Fit2, Lar, oxt, scb, SmB* |
| Butzlaff *et al*. (2015) | unbiased | 7746 RNAi lines | 89 | *alphaTub67C, alphaTub84B, Apf, Arp10, Arv1, bic, brun, CCT7, CG10979, CG14184, CG14621, CG15629, CG17327, CG18508, CG31259, CG32809, CG3500, CG3511, CG3808, CG42788, CG43427, CG5500, CG5986, CG6330, CG6418, CG6873, CG7896, CG8086, CG8108, CG8664, CG8785, Cwc25, CycJ, Cyp301a1, Dab, DCTN1-p150, DCTN2-p50, DCTN4-p62, DCTN5-p25, Dhc93AB, Drak, EloB, Fer1, Flo2, fzy, g, Gabat, Gdi, GstS1, hep, Hipk, HisRS, krz, l(2)05287, l(3)neo38, Lerp, Mad1, MED14, MRG15, Nedd8, Nelf-E, nord, Nrx-IV, Nuf2, par-6, Prosalpha7, Prosbeta2, Prp8, Rab30, Rpn9, RpS10a, Scamp, Sf3b5, Shroom, S-Lap2, Smg5, Snp, snRNP-U1-C, Spp, Stip1, Su(z)2, T3dh, Tet, ttv, Uck, Usp10, Vha16-1, Vha36-1, vnc* |
| Dourlen *et al*. (2016) | Candidate | Strains related to GWAs signals | 5 | *Amph, Eph, Fak, p130CAS, Rab3-GEF* |
| Lasagna-Reeves *et al*. (2016) | Candidate | 704 RNAi lines targeting *Drosophila* kinome (337 genes) | 13 | *AMPKalpha, bon, CaMKII, CASK, CG43143, Ddr, Eph, fbl, hop, Pdk, Rok, Unc-89, Wee1* |
| Dourlen (2017) | Candidate | Strains related to gene targets | 2 | *Diap1, p35* |
| Gusareva *et al*. (2018) | Candidate | Strains related to GWAs signals | 2 | *kibra, rhea* |

**Table S2. List of the alleles giving a genetic interaction with Tau.** Note that none of these alleles induces an eye phenotype in flies not expressing Tau.

| **Gene Symbol** | **Alleles** | **BDSC stock number** | **REP modification** |
| --- | --- | --- | --- |
| *amos* | amos[Tft]/CyO | 424 | ENH |
| *asp* | w[*]; P{w[+mC]=UAS-asp.GFP}attP2 | 65859 | ENH |
| *bbg* | y[1] w[67c23]; P{w[+mC] y[+mDint2]=EPgy2}bbg[EY21339] | 24094 | ENH |
|  | y[1] w[67c23]; P{w[+mC] y[+mDint2]=EPgy2}bbg[EY08296] | 17448 | ENH |
| *bnl* | ry[506] P{ry[+t7.2]=PZ}bnl[00857]/TM3, Sb[1] | 6384 | ENH |
|  | ry[506] P{ry[+t7.2]=PZ}bnl[06916]/TM3, ry[RK] Sb[1] Ser[1] | 11704 | ENH |
| *Cam* | UAS-Cam | / | ENH |
| *CG10077* | y[1] w[*]; Mi{y[+mDint2]=MIC}CG10077[MI10174] | 53827 | ENH |
| *CG12935* | w[1118]; P{w[+mC]=EP}CG12935[G18156] | 63257 | ENH |
| *CG1806* | y[1] w[67c23] P{w[+mC] y[+mDint2]=EPgy2}EY09439 | 19954 | ENH |
| *CG30015* | y[1] w[67c23]; P{w[+mC] y[+mDint2]=EPgy2}EY02749 | 15593 | ENH |
| *CG31886* | y[1] w[67c23]; P{y[+mDint2] w[BR.E.BR]=SUPor-P}CG31886[KG03608]/CyO | 13021 | ENH |
| *CG46385* | y[1] w[67c23]; P{w[+mC] y[+mDint2]=EPgy2}EY04353 | 16600 | ENH |
|  | w[1118]; P{w[+mC]=EP}CG46385[EP2618]/CyO | 17256 | ENH |
| *CG6701* | y[1] v[1]; P{y[+t7.7] v[+t1.8]=TRiP.JF02714}attP2 | 27560 | ENH |
|  | y[1] w[*]; Mi{y[+mDint2]=MIC}CG6701[MI12493]/SM6a | 57951 | ENH |
| *chb* | y[1] w[67c23]; P{w[+mC] y[+mDint2]=EPgy2}chb[EY22501] | 22566 | ENH |
| *CLIP-190* | y[1] v[1]; P{y[+t7.7] v[+t1.8]=TRiP.JF01206}attP2 | 31265 | ENH |
|  | w[1118]; Mi{ET1}CLIP-190[MB04222] | 24197 | ENH |
| *comm* | y[1] w[67c23]; P{w[+mC] y[+mDint2]=EPgy2}comm[EY10154]/TM3, Sb[1] Ser[1] | 17644 | ENH |
|  | P{w[+mC]=UAS-comm.MYC}1, w[*] | 66891 | ENH |
|  | y[1] v[1]; P{y[+t7.7] v[+t1.8]=TRiP.JF03018}attP2/TM3, Sb[1] | 28381 | ENH |
| *cpx* | w[*]; P{w[+mC]=UAS-cpx.H}3B | 39743 | ENH |
| *lncRNA:CR31044* | y[1] w[67c23]; P{w[+mC] y[+mDint2]=EPgy2}CR31044[EY03350] | 15435 | ENH |
| *Dmtn* | y[1] w[67c23]; P{w[+mC] y[+mDint2]=EPgy2}Dmtn[EY08071] | 22314 | ENH |
|  | y[1] w[*]; Mi{y[+mDint2]=MIC}Dmtn[MI08519] | 51083 | ENH |
| *dpr1* | w[*]; P{w[+mC]=UAS-dpr1.N}2/CyO | 25081 | ENH |
| *dpr18* | y[1] w[67c23] P{w[+mC] y[+mDint2]=EPgy2}EY02257 | 15410 | ENH |
|  | y[1] P{y[+mDint2] w[BR.E.BR]=SUPor-P}dpr18[KG02781] | 13231 | ENH |
| *e(y)3* | y[1] sc[*] v[1]; P{y[+t7.7] v[+t1.8]=TRiP.HMS00337}attP2 | 32346 | ENH |
|  | y[1] w[*] Mi{y[+mDint2]=MIC}e(y)3[MI11912] | 56847 | ENH |
| *eIF4EHP* | y[1] sc[*] v[1]; P{y[+t7.7] v[+t1.8]=TRiP.GL01035}attP2 | 36876 | ENH |
|  | y[1] sc[*] v[1]; P{y[+t7.7] v[+t1.8]=TRiP.HMS02703}attP40 | 43990 | ENH |
| *ena* | w[*]; P{w[+mW.hs]=FRT(w[hs])}G13 ena[210]/CyO | 25404 | ENH |
| *ens* | w[1118]; P{w[+mC]=EPg}ens[HP36480] | 21996 | ENH |
|  | w*; TI{TI}ensΔC P{neoFRT}80B/TM6B, Tb1 | 51318 | ENH |
| *Fer1HCH* | y[1] w[*]; Mi{y[+mDint2]=MIC}Fer1HCH[MI14026] | 59249 | ENH |
| *fru* | y[1] w[*]; Mi{y[+mDint2]=MIC}fru[MI15628]/TM3, Sb[1] Ser[1] | 61111 | ENH |
|  | w[*]; P{w[+mC]=UAS-fru[C].IR}C1; P{UAS-fru[C].IR}A9/TM6B, Tb[+] | 66694 | ENH |
| *fs(1)h* | C(1;YL)1, y[1] fs(1)h[rnc]/FM7c | 5278 | ENH |
| *gpp* | y[1] w[67c23]; P{w[+mC] y[+mDint2]=EPgy2}Dmtn[EY08071] | 22314 | ENH |
| *Gr47b* | y[1] w[67c23]; P{w[+mC] y[+mDint2]=EPgy2}EY02955 | 15890 | ENH |
| *h* | h[1] | 513 | ENH |
| *haf* | y[1] w[67c23]; P{w[+mC] y[+mDint2]=EPgy2}haf[EY11244] | 20278 | ENH |
| *hdc* | w[*]; P{w[+mC]=UAS-hdc.s.FLAG}3/TM3, Sb[1] | 64066 | ENH |
| *heph* | ry506 P{PZ}heph2/TM3, ryRK Sb1 Ser1 | 635 | ENH |
|  | y[1] w[67c23]; P{w[+mC] y[+mDint2]=EPgy2}heph[EY13208] | 20864 |  |
| *His2A:CG31618* | y[1] sc[*] v[1]; P{y[+t7.7] v[+t1.8]=TRiP.GL00226}attP2 | 35320 | ENH |
| *IP3K1* | y[1] w[*]; P{y[+t7.7]=Mae-UAS.6.11}IP3K1[UY530] | 6769 | ENH |
| *jim* | y[1] w[67c23]; P{w[+mC] y[+mDint2]=EPgy2}EY14392 | 20927 | ENH |
| *jumu* | w[*]; P{w[+mC]=EP}jumu[GE27806] | 26897 | ENH |
|  | P{ry[+t7.2]=PZ}jumu[06439] ry[506]/TM3, ry[RK] Sb[1] Ser[1] | 11683 | ENH |
| *kay* | y[1] w[67c23]; P{w[+mC] y[+mDint2]=EPgy2}kay[EY00283] | 15018 | ENH |
|  | y[1] w[67c23]; P{w[+mC] y[+mDint2]=EPgy2}kay[EY12710] | 20843 | ENH |
| *kuz* | w[*]; P{w[+mC]=UAS-kuz.DN}2 | 6578 | ENH |
|  | kuz[e29-4]/CyO | 5804 | ENH |
| *l(3)L1231* | y[1] w[67c23]; P{w[+mC] y[+mDint2]=EPgy2}EY04028 | 15708 | ENH |
| *mam* | w[*]; P{w[+mC]=UAS-mam.A}2 | 27743 | ENH |
| *Mbs* | P{PZ}Mbs03802 ry506/TM3, ryRK Sb1 Ser1 | 11607 | ENH |
| *Meltrin* | y[1] w[67c23]; P{w[+mC] y[+mDint2]=EPgy2}Meltrin[EY03366] | 15643 | ENH |
| *mura* | y[1] sc[*] v[1]; P{y[+t7.7] v[+t1.8]=TRiP.GL00121}attP2 | 35236 | ENH |
| *Not1* | y[1] v[1]; P{y[+t7.7] v[+t1.8]=TRiP.JF03096}attP2 | 28681 | ENH |
|  | y[1] v[1]; P{y[+t7.7] v[+t1.8]=TRiP.JF01135}attP2 | 31696 | ENH |
|  | y[1] sc[*] v[1]; P{y[+t7.7] v[+t1.8]=TRiP.HMS00526}attP2 | 32836 | ENH |
| *NSD* | y[1] w[67c23]; P{w[+mC] y[+mDint2]=EPgy2}NSD[EY01950] | 15384 | ENH |
| *Nsf2* | Nsf2[urd-2]/TM3, Sb[1] | 5049 | ENH |
|  | y[1] sc[*] v[1]; P{y[+t7.7] v[+t1.8]=TRiP.HMS01262}attP2 | 34914 | ENH |
| *numb* | w[*]; P{w[+mC]=UAS-numb.PTB.GFP}2/CyO | 51662 | ENH |
| *Oct-TyrR* | y[1] v[1]; P{y[+t7.7] v[+t1.8]=TRiP.JF02967}attP2 | 28332 | ENH |
| *pbl* | w[*]; P{w[+mC]=UAS-HA-pbl}1m/TM2 | 66160 | ENH |
|  | w[*]; pbl[3]/TM3, Sb[1] | 9358 | SUPP |
|  | y[1] w[*]; Mi{y[+mDint2]=MIC}pbl[MI07745]/TM3, Sb[1] Ser[1] | 44883 | ENH |
| *Pdk1* | w[1118]; P{w[+mC]=EP}Pdk1[EP3091] | 17260 | ENH |
|  | y[1] w[67c23]; P{y[+mDint2] w[BR.E.BR]=SUPor-P}Pdk1[KG01447] ry[506] | 13441 | ENH |
|  | y[1] v[1]; P{y[+t7.7] v[+t1.8]=TRiP.JF02807}attP2 | 27725 | ENH |
|  | y[1] sc[*] v[1]; P{y[+t7.7] v[+t1.8]=TRiP.GL00489}attP2 | 36071 | ENH |
| *Pdp1* | w[1118]; P{w[+mC]=UAS-Pdp1.WIZ}2 | 78088 | ENH |
| *Piezo* | w[*]; P{w[+mC]=UAS-Piezo.GFP}IIA-2/CyO; MKRS/TM6B, Tb[1] | 58772 | ENH |
|  | w[*]; sna[Sco]/CyO; P{w[+mC]=UAS-Piezo.GFP}IIIA/TM6B, Tb[1] | 58773 | ENH |
|  | w[*]; PBac{w[+mC]=RB5.WH5}Piezo[KO] | 58770 | ENH |
| *pyd* | y[1] w[67c23]; P{w[+mC] y[+mDint2]=EPgy2}pyd[EY01326] | 15342 | ENH |
| *qkr58E-3* | w[*]; qkr58E-3[minus]/CyO | 40966 | ENH |
| *Rab14* | P{w[+mC]=UASp-YFP.Rab14.S49N}5, y[1] w[*] | 50780 | ENH |
|  | y[1] w[*]; P{w[+mC]=UASp-YFP.Rab14.S49N}PH4alphaEFB[02] | 23264 | ENH |
|  | y[1] w[*]; P{w[+mC]=UASp-YFP.Rab14.S49N}Cse1[01] mdy[01]/CyO | 23263 | ENH |
|  | y[1] sc[*] v[1]; P{y[+t7.7] v[+t1.8]=TRiP.HMS01130}attP2 | 34654 | ENH |
| *raw* | y[1] w[67c23]; P{w[+mC]=lacW}raw[k01021]/CyO | 10363 | ENH |
|  | y1 w67c23; rawk03514 P{lacW}Fppsk03514/CyO | 10532 | ENH |
| *RpLP1* | w[1118]; RpLP1[beo]/CyO | 6399 | ENH |
| *RyR* | y1 w67c23; P{lacW}RyRk04913/CyO | 10559 | ENH |
| *scrib* | w[*]; P{w+mC]=UASp-scrib.R}B10 | 59078 | ENH |
| *sdt* | w[1118] P{w[+mC]=EP}sdt[EP1143] | 11266 | ENH |
|  | y[1] sdt[XN]/FM7c, sn[+] | 2181 | ENH |
|  | w[*] P{w[+mC]=EP}sdt[G1472] | 33545 | ENH |
| *SelD* | y[1]; P{y[+mDint2] w[BR.E.BR]=SUPor-P}SelD[KG08804]/CyO; ry[506] | 15136 | ENH |
|  | y[1] v[1]; P{y[+t7.7] v[+t1.8]=TRiP.JF03230}attP2 | 29553 | ENH |
|  | y[1] sc[*] v[1]; P{y[+t7.7] v[+t1.8]=TRiP.GL00002}attP2 | 35135 | ENH |
| *shn* | w*; P{neoFRT}42D mago3 shn3/CyO | 51289 | ENH |
| *skd* | w[*]; P{w[+mC]=UAS-skd.J}1/TM6B, Tb[1] | 63800 | SUPP |
| *SNF4Agamma* | w[*]; P{w[+mC]=UAS-SNF4Agamma.GFP}2 | 59030 | ENH |
|  | w[*]; P{w[+mC]=UAS-SNF4Agamma.GFP}3 | 59031 | ENH |
|  | y[1] w[*]; Mi{y[+mDint2]=MIC}SNF4Agamma[MI09417]/TM3, Sb[1] Ser[1] | 53104 | ENH |
| *Socs36E* | y[1] w[*]; P{w[+mC]=EP}Socs36E[G2762] | 27006 | ENH |
| *SppL* | y[1] w[67c23]; P{w[+mC] y[+mDint2]=EPgy2}SppL[EY22542] | 23113 | ENH |
| *Src42A* | y[1] sc[*] v[1]; P{y[+t7.7] v[+t1.8]=TRiP.HMS02755}attP40 | 44039 | ENH |
|  | y[1] sc[*] v[1]; P{y[+t7.7] v[+t1.8]=TRiP.HMC04138}attP2/TM3, Sb[1] | 55868 | ENH |
| *Src64B* | y[1] w[*]; P{w[+mC]=EP}Src64B[G7151] CG32246[G7151] | 27223 | ENH |
|  | y[1] v[1]; P{y[+t7.7] v[+t1.8]=TRiP.JF03234}attP2 | 30517 | ENH |
|  | y[1] sc[*] v[1]; P{y[+t7.7] v[+t1.8]=TRiP.GL00297}attP2 | 36062 | ENH |
| *Syn1* | y[1] w[67c23]; Mi{ET1}Syn1[MB01557] | 23336 | ENH |
|  | y[1] v[1]; P{y[+t7.7] v[+t1.8]=TRiP.JF02654}attP2 | 27504 | ENH |
| *tau* | y[1]w[1118]; P{w[+mc]= pUAST- dTau-1D4 [14636-1-3M] | / | ENH |
|  | y[1]w[1118]; P{w[+mc]= pUAST- dTau-1D4 [14636-2-7M] | / | ENH |
|  | w[1118]; P{w[+mC]=EP}tau[G4714] | 63271 | ENH |
|  | y[1] w[*]; Mi{y[+mDint2]=MIC}tau[MI11095] | 56122 | ENH |
|  | y[1] w[*]; Mi{y[+mDint2]=MIC}tau[MI08130] | 44755 | ENH |
|  | y[1] w[*]; Mi{y[+mDint2]=MIC}tau[MI03440] | 37602 | ENH |
|  | y[1] v[1]; P{y[+t7.7] v[+t1.8]=TRiP.HM05101}attP2 | 28891 | ENH |
| *Ten-m* | y[1] w[*]; M{w[+mC]=UAS-Ten-m.H}ZH-86Fb | 41569 | ENH |
|  | w[*]; P{y[+t7.7] w[+mC]=UAS-Ten-m.H}attP24 | 41570 | ENH |
|  | y[1] w[67c23]; P{w[+mC] y[+mDint2]=EPgy2}Ten-m[EY03921] | 19688 | ENH |
| *Thor* | w[*]; P{w[+mC]=UAS-Thor.wt}2 | 9147 | ENH |
|  | y[1] w[*]; Thor[2] | 9559 | ENH |
|  | w[*]; P{w[+mC]=UAS-Thor.LL}s/TM6C, cu[1] Sb[1] | 24854 | LETHAL |
| *Tl* | In(3LR)Tl[rv18], h[1] Diap1[1] st[1] cu[1] Tl[rv18] ca[1]/TM3, Ser[1] | 30913 | ENH |
| *Tsp96F* | y[1] w[*]; Mi{y[+mDint2]=MIC}Tsp96F[MI13401] | 59127 | ENH |
| *Usp47* | P{EPgy2}Usp47EY11769 | 20331 | ENH |
| *wde* | y[1] sc[*] v[1]; P{y[+t7.7] v[+t1.8]=TRiP.HMS00205}attP2 | 33339 | ENH |
|  | y[1] w[67c23]; P{w[+mC] y[+mDint2]=EPgy2}wde[EY00884]/CyO | 15045 | ENH |
|  | y[1] w[*]; Mi{y[+mDint2]=MIC}wde[MI02099]/SM6a | 34274 | ENH |

REP: rough eye phenotype; ENH: enhancer; SUPP: suppressor

**Table S3. List of genes up-regulated upon hTau^WT^ expression in *Drosophila* Primary Neurons**

|  | **Experiment N°1** | | **Experiment N°2** | |  | | | | |
| --- | --- | --- | --- | --- | --- | --- | --- | --- | --- |
| **Probe ID** | **Cy5 processed signal** | **Cy3 processed signal** | **Cy5 processed signal** | **Cy3 processed signal** | **FoldChge 1** | **Foldchge 2** | **Mean FoldChge** | **Gene Symbol** | **Gene Name** |
| A_09_P051886 | 455943,120 | 246340,050 | 663178,100 | 426462,340 | 1,851 | 1,555 | 1,703 | 18SrRNA:CR41548 | 18S ribosomal RNA:CR41548 |
| A_09_P116990 | 1312,425 | 705,126 | 2485,925 | 1299,011 | 1,861 | 1,914 | 1,887 | Acsl | Acyl-CoA synthetase long-chain |
| A_09_P041186 | 119,395 | 79,484 | 167,167 | 99,430 | 1,502 | 1,681 | 1,592 | Adh | Alcohol dehydrogenase |
| A_09_P072961 | 355,172 | 167,994 | 523,886 | 260,650 | 2,114 | 2,010 | 2,062 | Aduk | Another Drosophila Unc-51-like kinase |
| A_09_P135450 | 356,092 | 230,189 | 410,481 | 245,351 | 1,547 | 1,673 | 1,610 | Aduk | Another Drosophila Unc-51-like kinase |
| A_09_P146305 | 1802,730 | 1035,277 | 3216,657 | 1950,803 | 1,741 | 1,649 | 1,695 | Akh | Adipokinetic hormone |
| A_09_P187965 | 44,810 | 6,922 | 41,893 | 7,647 | 6,473 | 5,478 | 5,976 | Alk | Anaplastic lymphoma kinase |
| A_09_P209670 | 1571,209 | 451,712 | 2272,674 | 686,115 | 3,478 | 3,312 | 3,395 | alpha-Est10 | alpha-Esterase-10 |
| A_09_P121450 | 175,09 | 110,329 | 480,555 | 213,61 | 1,587 | 2,250 | 1,918 | amos | absent MD neurons and olfactory sensilla |
| A_09_P216350 | 1204,295 | 354,961 | 2432,396 | 902,785 | 3,393 | 2,694 | 3,044 | antr | antares |
| A_09_P058631 | 640,809 | 448,836 | 992,125 | 505,673 | 1,428 | 1,962 | 1,695 | antr | antares |
| A_09_P202545 | 230,803 | 136,943 | 357,149 | 235,312 | 1,685 | 1,518 | 1,602 | app | approximated |
| A_09_P169835 | 576,371 | 315,132 | 454,186 | 272,005 | 1,829 | 1,670 | 1,749 | aqz | aaquetzalli |
| A_09_P090320 | 8124,702 | 5288,252 | 15539,954 | 9690,621 | 1,536 | 1,604 | 1,570 | aqz | aaquetzalli |
| A_09_P106975 | 42,770 | 8,229 | 49,662 | 14,910 | 5,197 | 3,331 | 4,264 | Arp53D | Actin-related protein 53D |
| A_09_P013506 | 66,277 | 22,225 | 90,651 | 38,690 | 2,982 | 2,343 | 2,663 | Arp53D | Actin-related protein 53D |
| A_09_P019151 | 49,830 | 17,205 | 64,458 | 36,401 | 2,896 | 1,771 | 2,334 | Art2 | Arginine methyltransferase 2 |
| A_09_P091050 | 300,438 | 72,076 | 195,164 | 62,237 | 4,168 | 3,136 | 3,652 | asRNA:CR44063 | antisense RNA:CR44063 |
| A_09_P175795 | 200,122 | 107,128 | 349,033 | 170,727 | 1,868 | 2,044 | 1,956 | asRNA:CR45140 | antisense RNA:CR45140 |
| A_09_P214695 | 46,508 | 15,960 | 54,826 | 31,959 | 2,914 | 1,716 | 2,315 | Atg4a | Autophagy-related 4a |
| A_09_P177575 | 79,477 | 18,830 | 111,455 | 43,560 | 4,221 | 2,559 | 3,390 | AttA | Attacin-A |
| A_09_P102850 | 2051,372 | 1355,007 | 3661,695 | 2185,335 | 1,514 | 1,676 | 1,595 | bark | bark beetle |
| A_09_P167715 | 1142,951 | 640,190 | 1260,367 | 838,113 | 1,785 | 1,504 | 1,645 | beat-IIIb | beaten path IIIb |
| A_09_P072051 | 333,298 | 203,416 | 530,062 | 327,354 | 1,639 | 1,619 | 1,629 | beat-VII | beaten path VII |
| A_09_P009896 | 425613,500 | 197934,250 | 503998,500 | 250207,900 | 2,150 | 2,014 | 2,082 | betaTub56D | beta-Tubulin at 56D |
| A_09_P214885 | 150,143 | 90,696 | 303,413 | 172,906 | 1,655 | 1,755 | 1,705 | BHD | Birt-Hogg-Dube |
| A_09_P196595 | 20973,006 | 10109,760 | 24195,217 | 12499,931 | 2,075 | 1,936 | 2,005 | BI-1 | Bax Inhibitor-1 |
| A_09_P197380 | 10028,118 | 5887,344 | 14448,424 | 9527,375 | 1,703 | 1,517 | 1,610 | BI-1 | Bax Inhibitor-1 |
| A_09_P195810 | 90961,540 | 12961,605 | 149561,190 | 38498,240 | 7,018 | 3,885 | 5,451 | Bom778 | Bomanin 778 |
| A_09_P191650 | 4106,516 | 1335,666 | 5942,152 | 2422,417 | 3,075 | 2,453 | 2,764 | br | broad |
| A_09_P028946 | 894,082 | 227,935 | 1835,886 | 360,732 | 3,923 | 5,089 | 4,506 | BtbVII | BTB-protein-VII |
| A_09_P134890 | 2612,250 | 917,757 | 4652,583 | 1639,133 | 2,846 | 2,838 | 2,842 | BtbVII | BTB-protein-VII |
| A_09_P029511 | 726,625 | 256,793 | 1391,398 | 498,563 | 2,830 | 2,791 | 2,810 | BtbVII | BTB-protein-VII |
| A_09_P009516 | 3194,819 | 2131,162 | 5623,076 | 3455,523 | 1,499 | 1,627 | 1,563 | Btk29A | Btk family kinase at 29A |
| A_09_P226515 | 4793,643 | 1212,974 | 955,030 | 324,475 | 3,952 | 2,943 | 3,448 | bves | bves |
| A_09_P063291 | 2397,078 | 671,391 | 3128,414 | 1128,242 | 3,570 | 2,773 | 3,172 | bves | bves |
| A_09_P049346 | 193,935 | 83,373 | 486,642 | 212,398 | 2,326 | 2,291 | 2,309 | bw | brown |
| A_09_P173200 | 64,099 | 31,419 | 97,131 | 37,306 | 2,040 | 2,604 | 2,322 | ca | claret |
| A_09_P166095 | 294,853 | 39,311 | 572,351 | 109,945 | 7,501 | 5,206 | 6,353 | Cad88C | Cadherin 88C |
| A_09_P075111 | 763,250 | 174,212 | 1346,024 | 275,690 | 4,381 | 4,882 | 4,632 | Cad88C | Cadherin 88C |
| A_09_P068296 | 89,128 | 52,201 | 206,314 | 155,976 | 1,707 | 1,323 | 1,515 | Caf1-180 | Chromatin assembly factor 1, p180 subunit |
| A_09_P063256 | 710,174 | 201,330 | 1199,103 | 443,015 | 3,527 | 2,707 | 3,117 | CAH16 | Carbonic anhydrase 16 |
| A_09_P051221 | 129,716 | 72,253 | 239,196 | 147,260 | 1,795 | 1,624 | 1,710 | Calx | Na/Ca-exchange protein |
| A_09_P013191 | 452,183 | 176,738 | 801,388 | 386,237 | 2,558 | 2,075 | 2,317 | can | cannonball |
| A_09_P049331 | 2240,852 | 1316,518 | 3481,528 | 1823,426 | 1,702 | 1,909 | 1,806 | Cbp53E | Calbindin 53E |
| A_09_P173720 | 1231,757 | 318,507 | 1387,783 | 469,876 | 3,867 | 2,954 | 3,410 | CenG1A | Centaurin gamma 1A |
| A_09_P065251 | 1247,940 | 819,235 | 2181,549 | 1266,634 | 1,523 | 1,722 | 1,623 | CenG1A | Centaurin gamma 1A |
| A_09_P074291 | 41,485 | 21,131 | 73,111 | 44,212 | 1,963 | 1,654 | 1,808 | CG10035 | - |
| A_09_P023146 | 35,511 | 6,538 | 83,884 | 34,608 | 5,432 | 2,424 | 3,928 | CG10211 | - |
| A_09_P026041 | 136,502 | 50,889 | 298,149 | 144,063 | 2,682 | 2,070 | 2,376 | CG10505 | - |
| A_09_P068176 | 28,103 | 13,462 | 73,743 | 17,853 | 2,088 | 4,130 | 3,109 | CG10761 | - |
| A_09_P153165 | 8882,234 | 2102,972 | 1858,773 | 558,002 | 4,224 | 3,331 | 3,777 | CG10934 | - |
| A_09_P008796 | 1792,770 | 75,491 | 1829,075 | 63,493 | 23,748 | 28,807 | 26,278 | CG10936 | - |
| A_09_P101245 | 38,976 | 8,721 | 48,507 | 24,492 | 4,469 | 1,981 | 3,225 | CG11269 | - |
| A_09_P207515 | 67,702 | 7,612 | 38,250 | 13,129 | 8,894 | 2,913 | 5,904 | CG11286 | - |
| A_09_P026391 | 28,304 | 10,600 | 53,666 | 25,836 | 2,670 | 2,077 | 2,374 | CG11291 | - |
| A_09_P052751 | 35,229 | 6,299 | 45,916 | 4,014 | 5,593 | 11,439 | 8,516 | CG11353 | - |
| A_09_P199415 | 65,505 | 25,718 | 94,553 | 48,007 | 2,547 | 1,970 | 2,258 | CG11380 | - |
| A_09_P115380 | 2265,239 | 583,741 | 4061,800 | 1498,208 | 3,881 | 2,711 | 3,296 | CG11413 | - |
| A_09_P129240 | 358,115 | 201,997 | 458,617 | 242,146 | 1,773 | 1,894 | 1,833 | CG1143 | - |
| A_09_P067026 | 114,438 | 48,356 | 377,966 | 97,630 | 2,367 | 3,871 | 3,119 | CG11436 | - |
| A_09_P149140 | 52,871 | 18,991 | 70,673 | 36,126 | 2,784 | 1,956 | 2,370 | CG11808 | - |
| A_09_P223595 | 1460,436 | 970,521 | 1906,762 | 1178,403 | 1,505 | 1,618 | 1,561 | CG11873 | - |
| A_09_P212260 | 263,998 | 42,7 | 428,061 | 84,749 | 6,183 | 5,051 | 5,617 | CG11885 | - |
| A_09_P028351 | 1143,668 | 764,644 | 1774,026 | 1171,956 | 1,496 | 1,514 | 1,505 | CG12004 | - |
| A_09_P040501 | 150,827 | 96,792 | 320,744 | 150,606 | 1,558 | 2,130 | 1,844 | CG12231 | - |
| A_09_P025261 | 19,733 | 6,750 | 16,838 | 4,292 | 2,924 | 3,923 | 3,423 | CG12376 | - |
| A_09_P028796 | 95,424 | 15,115 | 176,657 | 51,986 | 6,313 | 3,398 | 4,856 | CG1246 | - |
| A_09_P112935 | 309,953 | 111,764 | 365,711 | 154,354 | 2,773 | 2,369 | 2,571 | CG12502 | - |
| A_09_P028146 | 257,454 | 115,077 | 240,157 | 129,513 | 2,237 | 1,854 | 2,046 | CG12502 | - |
| A_09_P005406 | 903,580 | 140,545 | 175,738 | 62,249 | 6,429 | 2,823 | 4,626 | CG12717 | - |
| A_09_P007786 | 31,280 | 7,589 | 46,428 | 27,429 | 4,122 | 1,693 | 2,907 | CG12857 | - |
| A_09_P006046 | 593,979 | 353,313 | 1089,496 | 626,863 | 1,681 | 1,738 | 1,710 | CG12910 | - |
| A_09_P072951 | 129,541 | 80,046 | 210,068 | 78,344 | 1,618 | 2,681 | 2,150 | CG12951 | - |
| A_09_P063726 | 1754,043 | 976,961 | 2511,453 | 1388,410 | 1,795 | 1,809 | 1,802 | CG12994 | - |
| A_09_P033811 | 92,699 | 31,006 | 97,306 | 56,804 | 2,990 | 1,713 | 2,351 | CG13023 | - |
| A_09_P102055 | 259,229 | 115,929 | 380,148 | 212,530 | 2,236 | 1,789 | 2,012 | CG13023 | - |
| A_09_P006341 | 1706,758 | 279,554 | 476,471 | 292,274 | 6,105 | 1,630 | 3,868 | CG13215 | - |
| A_09_P113775 | 3782,060 | 716,543 | 1115,648 | 637,873 | 5,278 | 1,749 | 3,514 | CG13215 | - |
| A_09_P101000 | 242,735 | 129,097 | 419,959 | 265,237 | 1,880 | 1,583 | 1,732 | CG13229 | - |
| A_09_P006291 | 302,865 | 189,670 | 597,905 | 378,445 | 1,597 | 1,580 | 1,588 | CG13229 | - |
| A_09_P054066 | 157,102 | 100,211 | 321,434 | 157,130 | 1,568 | 2,046 | 1,807 | CG13305 | - |
| A_09_P216540 | 48,948 | 17,157 | 84,068 | 23,874 | 2,853 | 3,521 | 3,187 | CG13488 | - |
| A_09_P026251 | 148,321 | 62,072 | 228,097 | 58,426 | 2,389 | 3,904 | 3,147 | CG13488 | - |
| A_09_P129180 | 1361,504 | 137,837 | 7018,523 | 1596,544 | 9,878 | 4,396 | 7,137 | CG13501 | - |
| A_09_P194865 | 1710,207 | 1003,663 | 4199,318 | 2210,144 | 1,704 | 1,900 | 1,802 | CG13506 | - |
| A_09_P189600 | 59,105 | 30,114 | 118,741 | 39,535 | 1,963 | 3,003 | 2,483 | CG13667 | - |
| A_09_P052821 | 88,112 | 49,556 | 137,374 | 69,223 | 1,778 | 1,985 | 1,881 | CG13707 | - |
| A_09_P070491 | 44,646 | 13,582 | 41,035 | 23,690 | 3,287 | 1,732 | 2,510 | CG13838 | - |
| A_09_P070186 | 53,063 | 14,103 | 70,926 | 26,711 | 3,762 | 2,655 | 3,209 | CG13855 | - |
| A_09_P027986 | 42993,316 | 12573,696 | 34041,434 | 13258,971 | 3,419 | 2,567 | 2,993 | CG13891 | - |
| A_09_P023706 | 955,602 | 289,238 | 1196,177 | 416,634 | 3,304 | 2,871 | 3,087 | CG13970 | - |
| A_09_P072361 | 97,628 | 51,648 | 148,081 | 91,721 | 1,890 | 1,614 | 1,752 | CG13972 | - |
| A_09_P113130 | 1044,815 | 576,779 | 2328,547 | 1498,106 | 1,811 | 1,554 | 1,683 | CG14075 | - |
| A_09_P055721 | 236,972 | 145,558 | 404,024 | 244,301 | 1,628 | 1,654 | 1,641 | CG14109 | - |
| A_09_P210725 | 151,028 | 75,215 | 220,340 | 105,345 | 2,008 | 2,092 | 2,050 | CG14143 | - |
| A_09_P071976 | 106,259 | 62,539 | 104,375 | 48,488 | 1,699 | 2,153 | 1,926 | CG14237 | - |
| A_09_P071981 | 195,253 | 106,443 | 250,190 | 151,366 | 1,834 | 1,653 | 1,744 | CG14238 | - |
| A_09_P020711 | 68,817 | 20,508 | 133,094 | 51,726 | 3,356 | 2,573 | 2,964 | CG14277 | - |
| A_09_P069131 | 19,583 | 6,633 | 24,152 | 5,231 | 2,953 | 4,617 | 3,785 | CG14282 | - |
| A_09_P018136 | 77,074 | 39,971 | 71,764 | 33,013 | 1,928 | 2,174 | 2,051 | CG14339 | - |
| A_09_P038731 | 85,740 | 37,265 | 393,755 | 39,337 | 2,301 | 10,010 | 6,155 | CG14410 | - |
| A_09_P062791 | 114,965 | 52,448 | 209,527 | 83,086 | 2,192 | 2,522 | 2,357 | CG14416 | - |
| A_09_P130240 | 61,031 | 11,172 | 119,908 | 50,757 | 5,463 | 2,362 | 3,913 | CG14439 | - |
| A_09_P035836 | 154,288 | 77,236 | 192,582 | 116,947 | 1,998 | 1,647 | 1,822 | CG14451 | - |
| A_09_P035816 | 37,061 | 7,396 | 62,852 | 10,454 | 5,011 | 6,012 | 5,512 | CG14453 | - |
| A_09_P212425 | 1191,465 | 706,048 | 2010,712 | 1185,462 | 1,688 | 1,696 | 1,692 | CG15021 | - |
| A_09_P052696 | 1045,036 | 615,231 | 1951,919 | 1243,088 | 1,699 | 1,570 | 1,634 | CG15021 | - |
| A_09_P216475 | 214,085 | 119,895 | 194,253 | 113,974 | 1,786 | 1,704 | 1,745 | CG15127 | - |
| A_09_P065836 | 352,163 | 196,504 | 612,842 | 269,288 | 1,792 | 2,276 | 2,034 | CG15282 | - |
| A_09_P018951 | 228,169 | 134,892 | 250,961 | 148,785 | 1,691 | 1,687 | 1,689 | CG15412 | - |
| A_09_P019116 | 158,363 | 73,006 | 155,688 | 96,618 | 2,169 | 1,611 | 1,890 | CG15422 | - |
| A_09_P130600 | 40,165 | 6,212 | 37,030 | 4,438 | 6,466 | 8,344 | 7,405 | CG15425 | - |
| A_09_P019146 | 56,007 | 13,912 | 58,528 | 7,149 | 4,026 | 8,187 | 6,106 | CG15425 | - |
| A_09_P061256 | 47,483 | 15,455 | 56,401 | 33,761 | 3,072 | 1,671 | 2,371 | CG15526 | - |
| A_09_P061246 | 124,180 | 9,196 | 119,537 | 14,530 | 13,504 | 8,227 | 10,865 | CG15528 | - |
| A_09_P128415 | 220,049 | 8,615 | 517,412 | 89,461 | 25,543 | 5,784 | 15,663 | CG15549 | - |
| A_09_P066956 | 23,815 | 7,022 | 26,686 | 12,933 | 3,392 | 2,063 | 2,728 | CG15570 | - |
| A_09_P036726 | 757,248 | 206,829 | 901,100 | 235,928 | 3,661 | 3,819 | 3,740 | CG15594 | - |
| A_09_P039072 | 269,791 | 168,380 | 477,646 | 231,067 | 1,602 | 2,067 | 1,835 | CG15646 | - |
| A_09_P025986 | 39,640 | 6,848 | 78,761 | 8,542 | 5,789 | 9,220 | 7,504 | CG15657 | - |
| A_09_P038076 | 99,472 | 30,794 | 65,466 | 26,370 | 3,230 | 2,483 | 2,856 | CG15731 | - |
| A_09_P067402 | 880,422 | 504,024 | 690,430 | 486,312 | 1,747 | 1,420 | 1,583 | CG15765 | - |
| A_09_P030741 | 410,060 | 86,887 | 431,210 | 143,682 | 4,719 | 3,001 | 3,860 | CG15865 | - |
| A_09_P145755 | 51317,402 | 8703,313 | 86057,940 | 28070,492 | 5,896 | 3,066 | 4,481 | CG1628 | - |
| A_09_P145860 | 695,578 | 444,770 | 992,304 | 643,856 | 1,564 | 1,541 | 1,553 | CG1636 | - |
| A_09_P072956 | 463,064 | 277,316 | 639,713 | 401,228 | 1,670 | 1,594 | 1,632 | CG16749 | - |
| A_09_P001931 | 51,486 | 21,693 | 77,619 | 45,751 | 2,373 | 1,697 | 2,035 | CG16984 | - |
| A_09_P112795 | 59,264 | 26,689 | 135,214 | 82,844 | 2,221 | 1,632 | 1,926 | CG17048 | - |
| A_09_P070526 | 27,974 | 9,386 | 27,721 | 15,539 | 2,980 | 1,784 | 2,382 | CG17110 | - |
| A_09_P070501 | 112,093 | 16,379 | 134,192 | 85,000 | 6,844 | 1,579 | 4,211 | CG17121 | - |
| A_09_P061991 | 32,516 | 9,224 | 24,388 | 17,169 | 3,525 | 1,420 | 2,473 | CG17167 | - |
| A_09_P020821 | 2549,501 | 529,526 | 2328,830 | 700,415 | 4,815 | 3,325 | 4,070 | CG17294 | - |
| A_09_P184610 | 392,599 | 96,017 | 259,237 | 78,721 | 4,089 | 3,293 | 3,691 | CG17544 | - |
| A_09_P205060 | 61,342 | 17,204 | 70,203 | 33,763 | 3,566 | 2,079 | 2,822 | CG17598 | - |
| A_09_P009131 | 9968,807 | 22,089 | 14338,947 | 18,685 | 451,302 | 767,404 | 609,353 | CG17669 | - |
| A_09_P115505 | 9639,363 | 119,256 | 15367,579 | 158,366 | 80,829 | 97,038 | 88,934 | CG17669 | - |
| A_09_P225680 | 4217,077 | 2503,919 | 7753,830 | 3656,190 | 1,684 | 2,121 | 1,902 | CG17716 | - |
| A_09_P204280 | 195,508 | 111,959 | 525,075 | 338,614 | 1,746 | 1,551 | 1,648 | CG17716 | - |
| A_09_P034806 | 244,445 | 151,003 | 377,211 | 244,394 | 1,619 | 1,543 | 1,581 | CG17732 | - |
| A_09_P006771 | 117,881 | 74,760 | 255,549 | 159,007 | 1,577 | 1,607 | 1,592 | CG17739 | - |
| A_09_P022831 | 177,694 | 50,251 | 180,672 | 112,166 | 3,536 | 1,611 | 2,573 | CG17928 | - |
| A_09_P122205 | 68,320 | 21,282 | 159,604 | 5,168 | 3,210 | 30,886 | 17,048 | CG17991 | - |
| A_09_P072271 | 989,835 | 253,802 | 1073,536 | 358,002 | 3,900 | 2,999 | 3,449 | CG17991 | - |
| A_09_P040876 | 2622,936 | 740,063 | 1710,943 | 625,414 | 3,544 | 2,736 | 3,140 | CG1835 | - |
| A_09_P191845 | 2475,618 | 648,646 | 1422,207 | 811,147 | 3,817 | 1,753 | 2,785 | CG2186 | - |
| A_09_P068066 | 38,236 | 7,710 | 61,557 | 40,373 | 4,959 | 1,525 | 3,242 | CG2233 | - |
| A_09_P037816 | 57,086 | 36,207 | 165,087 | 75,889 | 1,577 | 2,175 | 1,876 | CG2444 | - |
| A_09_P065211 | 139,807 | 60,622 | 230,906 | 127,964 | 2,306 | 1,804 | 2,055 | CG30116 | - |
| A_09_P057901 | 208,313 | 26,088 | 340,559 | 77,332 | 7,985 | 4,404 | 6,194 | CG30195 | - |
| A_09_P057916 | 440,606 | 185,210 | 800,604 | 359,394 | 2,379 | 2,228 | 2,303 | CG30203 | - |
| A_09_P058031 | 144,153 | 55,401 | 172,551 | 112,665 | 2,602 | 1,532 | 2,067 | CG30285 | - |
| A_09_P058371 | 260,479 | 48,864 | 135,541 | 53,912 | 5,331 | 2,514 | 3,922 | CG30413 | - |
| A_09_P058996 | 65,173 | 39,209 | 140,666 | 67,303 | 1,662 | 2,090 | 1,876 | CG31100 | - |
| A_09_P163175 | 83,710 | 18,242 | 83,302 | 26,489 | 4,589 | 3,145 | 3,867 | CG31195 | - |
| A_09_P059376 | 1063,957 | 160,503 | 637,882 | 128,668 | 6,629 | 4,958 | 5,793 | CG31226 | - |
| A_09_P221820 | 1683,379 | 238,462 | 366,382 | 93,842 | 7,059 | 3,904 | 5,482 | CG31226 | - |
| A_09_P059941 | 128,501 | 8,234 | 142,339 | 5,245 | 15,606 | 27,138 | 21,372 | CG31469 | - |
| A_09_P060031 | 110,730 | 49,472 | 103,719 | 49,802 | 2,238 | 2,083 | 2,160 | CG31524 | - |
| A_09_P018796 | 1683,183 | 998,845 | 3384,566 | 1388,457 | 1,685 | 2,438 | 2,061 | CG3165 | - |
| A_09_P018651 | 136,037 | 72,461 | 271,795 | 144,674 | 1,877 | 1,879 | 1,878 | CG31689 | - |
| A_09_P060491 | 31,737 | 6,073 | 36,068 | 18,281 | 5,226 | 1,973 | 3,599 | CG31704 | - |
| A_09_P065911 | 118,838 | 64,261 | 198,652 | 116,142 | 1,849 | 1,710 | 1,780 | CG31771 | - |
| A_09_P013921 | 2117,466 | 385,627 | 719,535 | 418,091 | 5,491 | 1,721 | 3,606 | CG31849 | - |
| A_09_P217070 | 110,852 | 52,486 | 118,541 | 60,912 | 2,112 | 1,946 | 2,029 | CG31861 | - |
| A_09_P166490 | 129,660 | 71,176 | 152,709 | 98,388 | 1,822 | 1,552 | 1,687 | CG31861 | - |
| A_09_P013981 | 40,470 | 12,796 | 74,523 | 23,748 | 3,163 | 3,138 | 3,150 | CG31867 | - |
| A_09_P014176 | 43,123 | 40,983 | 63,719 | 29,819 | 1,052 | 2,137 | 1,595 | CG31954 | - |
| A_09_P014176 | 43,123 | 40,983 | 63,719 | 29,819 | 1,052 | 2,137 | 1,595 | CG31954 | - |
| A_09_P014551 | 552,502 | 227,897 | 569,555 | 376,865 | 2,424 | 1,511 | 1,968 | CG32091 | - |
| A_09_P014691 | 2582,447 | 1585,575 | 3951,754 | 2283,290 | 1,629 | 1,731 | 1,680 | CG32137 | - |
| A_09_P025906 | 124,597 | 16,448 | 332,803 | 58,842 | 7,575 | 5,656 | 6,616 | CG3216 | - |
| A_09_P015036 | 890,355 | 170,165 | 1255,366 | 396,602 | 5,232 | 3,165 | 4,199 | CG32243 | - |
| A_09_P142210 | 887,964 | 407,280 | 1189,504 | 596,819 | 2,180 | 1,993 | 2,087 | CG32243 | - |
| A_09_P015331 | 4322,322 | 217,855 | 6136,046 | 393,008 | 19,840 | 15,613 | 17,727 | CG32354 | - |
| A_09_P016061 | 1313,742 | 183,888 | 3355,357 | 890,080 | 7,144 | 3,770 | 5,457 | CG32645 | - |
| A_09_P016081 | 160,715 | 48,543 | 330,844 | 101,834 | 3,311 | 3,249 | 3,280 | CG32650 | - |
| A_09_P160270 | 1328,043 | 590,169 | 2957,579 | 935,794 | 2,250 | 3,161 | 2,705 | CG32850 | - |
| A_09_P016846 | 746,450 | 462,149 | 1362,223 | 801,738 | 1,615 | 1,699 | 1,657 | CG33111 | - |
| A_09_P177020 | 6264,451 | 1545,125 | 4326,201 | 1380,709 | 4,054 | 3,133 | 3,594 | CG33235 | - |
| A_09_P191930 | 61,856 | 29,805 | 149,887 | 76,508 | 2,075 | 1,959 | 2,017 | CG3328 | - |
| A_09_P000066 | 50,950 | 9,856 | 23,929 | 14,333 | 5,169 | 1,670 | 3,419 | CG33511 | - |
| A_09_P178210 | 198,307 | 51,477 | 608,482 | 48,229 | 3,852 | 12,617 | 8,234 | CG33655 | - |
| A_09_P000466 | 73,286 | 16,928 | 81,585 | 28,053 | 4,329 | 2,908 | 3,619 | CG33681 | - |
| A_09_P198075 | 205,741 | 71,326 | 273,390 | 76,557 | 2,885 | 3,571 | 3,228 | CG33722 | - |
| A_09_P217360 | 143,911 | 59,811 | 210,585 | 108,526 | 2,406 | 1,940 | 2,173 | CG33926 | - |
| A_09_P000821 | 459,966 | 291,762 | 761,448 | 491,873 | 1,577 | 1,548 | 1,562 | CG33926 | - |
| A_09_P002601 | 88,185 | 53,694 | 121,542 | 80,486 | 1,642 | 1,510 | 1,576 | CG34124 | - |
| A_09_P047781 | 1649,536 | 466,551 | 884,458 | 339,604 | 3,536 | 2,604 | 3,070 | CG34175 | - |
| A_09_P101745 | 205,792 | 22,023 | 124,297 | 20,210 | 9,345 | 6,150 | 7,747 | CG34221 | - |
| A_09_P003291 | 64,772 | 14,168 | 42,208 | 10,942 | 4,572 | 3,857 | 4,215 | CG34224 | - |
| A_09_P003496 | 755,051 | 369,886 | 560,779 | 274,356 | 2,041 | 2,044 | 2,043 | CG34265 | - |
| A_09_P003501 | 299,825 | 66,460 | 154,509 | 46,792 | 4,511 | 3,302 | 3,907 | CG34266 | - |
| A_09_P101715 | 264,267 | 63,756 | 369,276 | 110,436 | 4,145 | 3,344 | 3,744 | CG34288 | - |
| A_09_P003611 | 245,129 | 144,844 | 321,954 | 177,218 | 1,692 | 1,817 | 1,755 | CG34288 | - |
| A_09_P176335 | 32840,125 | 9369,375 | 14265,818 | 5283,782 | 3,505 | 2,700 | 3,102 | CG34347 | - |
| A_09_P191970 | 2204,315 | 1278,754 | 3725,217 | 2397,752 | 1,724 | 1,554 | 1,639 | CG34347 | - |
| A_09_P197545 | 1346,760 | 645,931 | 2650,394 | 1221,939 | 2,085 | 2,169 | 2,127 | CG34376 | - |
| A_09_P004041 | 1421,247 | 801,684 | 1940,964 | 1179,614 | 1,773 | 1,645 | 1,709 | CG34376 | - |
| A_09_P004101 | 707,718 | 101,602 | 2086,855 | 496,967 | 6,966 | 4,199 | 5,582 | CG34386 | - |
| A_09_P018526 | 100,372 | 36,555 | 189,296 | 83,930 | 2,746 | 2,255 | 2,501 | CG3597 | - |
| A_09_P163295 | 471,487 | 212,052 | 902,784 | 388,397 | 2,223 | 2,324 | 2,274 | CG3655 | - |
| A_09_P182255 | 175,643 | 69,774 | 231,578 | 124,333 | 2,517 | 1,863 | 2,190 | CG3655 | - |
| A_09_P181990 | 32,979 | 8,317 | 21,767 | 3,625 | 3,965 | 6,005 | 4,985 | CG3842 | - |
| A_09_P001231 | 72,620 | 35,530 | 95,614 | 57,901 | 2,044 | 1,651 | 1,848 | CG40001 | - |
| A_09_P021201 | 9405,851 | 1330,580 | 6518,953 | 1443,023 | 7,069 | 4,518 | 5,793 | CG4017 | - |
| A_09_P001756 | 217,880 | 27,201 | 302,179 | 18,779 | 8,010 | 16,091 | 12,051 | CG40385 | - |
| A_09_P004621 | 139,618 | 35,630 | 284,476 | 75,139 | 3,919 | 3,786 | 3,852 | CG40600 | - |
| A_09_P004626 | 164,018 | 55,021 | 302,636 | 89,700 | 2,981 | 3,374 | 3,177 | CG40600 | - |
| A_09_P182275 | 55,842 | 20,176 | 109,269 | 56,839 | 2,768 | 1,922 | 2,345 | CG40813 | - |
| A_09_P004836 | 1153,884 | 736,200 | 2269,417 | 1093,359 | 1,567 | 2,076 | 1,821 | CG40971 | - |
| A_09_P164245 | 171,434 | 84,520 | 218,614 | 129,498 | 2,028 | 1,688 | 1,858 | CG4098 | - |
| A_09_P002351 | 44,667 | 5,929 | 53,745 | 12,125 | 7,534 | 4,433 | 5,983 | CG41072 | - |
| A_09_P005171 | 7527,029 | 2037,489 | 4387,560 | 1552,539 | 3,694 | 2,826 | 3,260 | CG41520 | - |
| A_09_P005481 | 76,564 | 6,904 | 42,448 | 10,378 | 11,090 | 4,090 | 7,590 | CG41551 | - |
| A_09_P005491 | 3777,345 | 2109,397 | 7465,879 | 2965,133 | 1,791 | 2,518 | 2,154 | CG41553 | - |
| A_09_P005511 | 2456,629 | 1127,613 | 5441,570 | 1738,132 | 2,179 | 3,131 | 2,655 | CG41557 | - |
| A_09_P114470 | 1694,565 | 781,173 | 3214,544 | 1243,018 | 2,169 | 2,586 | 2,378 | CG41557 | - |
| A_09_P005526 | 567,591 | 277,335 | 1945,504 | 619,042 | 2,047 | 3,143 | 2,595 | CG41560 | - |
| A_09_P005556 | 900,348 | 280,726 | 1371,372 | 459,314 | 3,207 | 2,986 | 3,096 | CG41569 | - |
| A_09_P048451 | 3722,681 | 1548,117 | 7496,328 | 2470,555 | 2,405 | 3,034 | 2,719 | CG41579 | - |
| A_09_P048466 | 568,668 | 261,256 | 1408,650 | 475,989 | 2,177 | 2,959 | 2,568 | CG41584 | - |
| A_09_P048486 | 1405,981 | 650,161 | 3096,601 | 921,536 | 2,163 | 3,360 | 2,761 | CG41589 | - |
| A_09_P002259 | 4371,854 | 2349,177 | 7267,737 | 2796,907 | 1,861 | 2,598 | 2,230 | CG41589 | - |
| A_09_P048516 | 1580,481 | 466,873 | 1677,591 | 496,232 | 3,385 | 3,381 | 3,383 | CG41616 | - |
| A_09_P037566 | 368,559 | 76,627 | 182,663 | 69,660 | 4,810 | 2,622 | 3,716 | CG42249 | - |
| A_09_P110655 | 1131,388 | 259,277 | 1425,422 | 416,948 | 4,364 | 3,419 | 3,891 | CG42326 | - |
| A_09_P217760 | 471,540 | 293,720 | 961,563 | 611,095 | 1,605 | 1,574 | 1,589 | CG42327 | - |
| A_09_P164145 | 77,456 | 33,806 | 99,691 | 65,038 | 2,291 | 1,533 | 1,912 | CG42531 | - |
| A_09_P015051 | 1558,705 | 998,518 | 2602,358 | 988,656 | 1,561 | 2,632 | 2,097 | CG42540 | - |
| A_09_P203515 | 178,471 | 107,859 | 182,614 | 95,347 | 1,655 | 1,915 | 1,785 | CG42541 | - |
| A_09_P180910 | 4130,471 | 489,855 | 3401,401 | 710,802 | 8,432 | 4,785 | 6,609 | CG42578 | - |
| A_09_P103205 | 3968,511 | 1051,024 | 9265,403 | 3106,457 | 3,776 | 2,983 | 3,379 | CG42637 | - |
| A_09_P026526 | 1495,029 | 550,189 | 1040,729 | 666,837 | 2,717 | 1,561 | 2,139 | CG4269 | - |
| A_09_P018501 | 1586,040 | 417,518 | 654,539 | 212,544 | 3,799 | 3,080 | 3,439 | CG4271 | - |
| A_09_P013606 | 106,728 | 50,331 | 117,572 | 71,202 | 2,121 | 1,651 | 1,886 | CG42750 | - |
| A_09_P196893 | 165,338 | 57,512 | 219,469 | 113,772 | 2,875 | 1,929 | 2,402 | CG42788 | - |
| A_09_P022671 | 167,962 | 66,986 | 151,674 | 65,851 | 2,507 | 2,303 | 2,405 | CG42810 | - |
| A_09_P160030 | 328,777 | 53,728 | 142,370 | 75,690 | 6,119 | 1,881 | 4,000 | CG43051 | - |
| A_09_P199250 | 53,036 | 18,859 | 67,912 | 33,154 | 2,812 | 2,048 | 2,430 | CG43078 | - |
| A_09_P181135 | 522,094 | 131,713 | 148,350 | 49,971 | 3,964 | 2,969 | 3,466 | CG43139 | - |
| A_09_P102960 | 531,486 | 313,754 | 920,951 | 579,842 | 1,694 | 1,588 | 1,641 | CG43163 | - |
| A_09_P215765 | 33,580 | 6,281 | 55,341 | 30,802 | 5,346 | 1,797 | 3,571 | CG43340 | - |
| A_09_P175000 | 70,984 | 17,857 | 77,811 | 32,204 | 3,975 | 2,416 | 3,196 | CG43658 | - |
| A_09_P205010 | 1641,054 | 494,314 | 892,015 | 311,257 | 3,320 | 2,866 | 3,093 | CG43736 | - |
| A_09_P002036 | 1687,341 | 464,925 | 1809,325 | 774,179 | 3,629 | 2,337 | 2,983 | CG43736 | - |
| A_09_P144675 | 148,612 | 66,026 | 134,132 | 67,272 | 2,251 | 1,994 | 2,122 | CG43800 | - |
| A_09_P197760 | 8532,383 | 5240,254 | 10370,923 | 5989,891 | 1,628 | 1,731 | 1,680 | CG44774 | - |
| A_09_P046166 | 3047,320 | 1950,218 | 5126,743 | 3301,181 | 1,563 | 1,553 | 1,558 | CG45050 | - |
| A_09_P027476 | 852,966 | 440,655 | 1318,418 | 654,290 | 1,936 | 2,015 | 1,975 | CG4563 | - |
| A_09_P169130 | 114,870 | 54,800 | 134,271 | 85,392 | 2,096 | 1,572 | 1,834 | CG4563 | - |
| A_09_P169420 | 45,327 | 7,693 | 51,976 | 15,005 | 5,892 | 3,464 | 4,678 | CG4587 | - |
| A_09_P067826 | 75,338 | 7,622 | 321,021 | 34,575 | 9,884 | 9,285 | 9,585 | CG4593 | - |
| A_09_P137410 | 158,687 | 46,024 | 201,151 | 3,705 | 3,448 | 54,292 | 28,870 | CG46280 | - |
| A_09_P174930 | 186,751 | 117,681 | 223,856 | 128,031 | 1,587 | 1,748 | 1,668 | CG46385 | - |
| A_09_P052906 | 951,431 | 94,059 | 1357,626 | 268,935 | 10,115 | 5,048 | 7,582 | CG4669 | - |
| A_09_P182425 | 305,317 | 97,680 | 686,259 | 265,205 | 3,126 | 2,588 | 2,857 | CG4730 | - |
| A_09_P033581 | 197,279 | 73,748 | 474,786 | 290,012 | 2,675 | 1,637 | 2,156 | CG4998 | - |
| A_09_P035156 | 137,722 | 27,343 | 166,501 | 53,280 | 5,037 | 3,125 | 4,081 | CG5078 | - |
| A_09_P150545 | 122,832 | 34,463 | 145,434 | 90,713 | 3,564 | 1,603 | 2,584 | CG5172 | - |
| A_09_P125620 | 46,786 | 13,300 | 62,030 | 36,327 | 3,518 | 1,708 | 2,613 | CG5188 | - |
| A_09_P172700 | 132,087 | 19,344 | 158,561 | 11,616 | 6,828 | 13,650 | 10,239 | CG5262 | - |
| A_09_P073886 | 72,274 | 35,355 | 148,535 | 62,184 | 2,044 | 2,389 | 2,216 | CG5342 | - |
| A_09_P129360 | 1628,561 | 837,090 | 2118,802 | 1055,636 | 1,946 | 2,007 | 1,976 | CG5348 | - |
| A_09_P008476 | 1964,475 | 1143,773 | 3026,378 | 1669,787 | 1,718 | 1,812 | 1,765 | CG5348 | - |
| A_09_P018266 | 85,085 | 51,237 | 96,262 | 60,701 | 1,661 | 1,586 | 1,623 | CG5561 | - |
| A_09_P054176 | 2346,612 | 1347,927 | 2888,554 | 1755,312 | 1,741 | 1,646 | 1,693 | CG5644 | - |
| A_09_P021376 | 47,495 | 18,306 | 84,330 | 44,362 | 2,594 | 1,901 | 2,248 | CG5731 | - |
| A_09_P188960 | 75,085 | 21,414 | 453,691 | 149,632 | 3,506 | 3,032 | 3,269 | CG5790 | - |
| A_09_P167125 | 73,878 | 6,843 | 63,282 | 16,308 | 10,796 | 3,880 | 7,338 | CG5830 | - |
| A_09_P186925 | 1624,763 | 259,156 | 1726,054 | 442,359 | 6,269 | 3,902 | 5,086 | CG5830 | - |
| A_09_P181580 | 124,808 | 35,130 | 181,517 | 43,914 | 3,553 | 4,133 | 3,843 | CG5830 | - |
| A_09_P033336 | 6698,318 | 1780,315 | 7442,622 | 2285,758 | 3,762 | 3,256 | 3,509 | CG5830 | - |
| A_09_P168555 | 282,524 | 90,810 | 449,879 | 132,479 | 3,111 | 3,396 | 3,254 | CG5830 | - |
| A_09_P191600 | 91,268 | 22,598 | 75,525 | 33,090 | 4,039 | 2,282 | 3,161 | CG5830 | - |
| A_09_P161585 | 2195,396 | 766,634 | 3866,994 | 1168,841 | 2,864 | 3,308 | 3,086 | CG5830 | - |
| A_09_P072246 | 223,618 | 143,927 | 183,875 | 161,570 | 1,554 | 1,138 | 1,346 | CG5882 | - |
| A_09_P022856 | 228,548 | 115,573 | 242,856 | 132,705 | 1,978 | 1,830 | 1,904 | CG6012 | - |
| A_09_P071951 | 122,420 | 57,202 | 176,880 | 108,268 | 2,140 | 1,634 | 1,887 | CG6154 | - |
| A_09_P124315 | 2401,994 | 1184,249 | 2518,770 | 1302,184 | 2,028 | 1,934 | 1,981 | CG6357 | - |
| A_09_P007441 | 5076,096 | 2994,967 | 5602,169 | 3187,826 | 1,695 | 1,757 | 1,726 | CG6357 | - |
| A_09_P180870 | 914,570 | 555,506 | 2637,170 | 569,984 | 1,646 | 4,627 | 3,137 | CG6428 | - |
| A_09_P008506 | 45,599 | 16,919 | 34,749 | 11,454 | 2,695 | 3,034 | 2,865 | CG6435 | - |
| A_09_P209270 | 157,350 | 58,858 | 216,024 | 120,411 | 2,673 | 1,794 | 2,234 | CG6701 | - |
| A_09_P054576 | 435,098 | 282,211 | 616,451 | 382,012 | 1,542 | 1,614 | 1,578 | CG6709 | - |
| A_09_P217375 | 2520,428 | 1671,578 | 3283,776 | 2090,310 | 1,508 | 1,571 | 1,539 | CG6709 | - |
| A_09_P053971 | 76,091 | 9,150 | 101,810 | 5,554 | 8,316 | 18,331 | 13,323 | CG6983 | - |
| A_09_P069231 | 17,633 | 6,401 | 37,730 | 4,301 | 2,755 | 8,772 | 5,764 | CG7342 | - |
| A_09_P184460 | 58,349 | 20,091 | 77,174 | 37,170 | 2,904 | 2,076 | 2,490 | CG7720 | - |
| A_09_P174495 | 229,686 | 42,329 | 366,410 | 124,765 | 5,426 | 2,937 | 4,182 | CG7785 | - |
| A_09_P037196 | 32,391 | 5,957 | 40,244 | 8,723 | 5,438 | 4,613 | 5,026 | CG7900 | - |
| A_09_P217635 | 53,662 | 9,819 | 85,563 | 19,755 | 5,465 | 4,331 | 4,898 | CG7900 | - |
| A_09_P174300 | 65,434 | 12,090 | 87,790 | 26,666 | 5,412 | 3,292 | 4,352 | CG7900 | - |
| A_09_P195560 | 487,203 | 244,453 | 665,931 | 425,524 | 1,993 | 1,565 | 1,779 | CG7956 | - |
| A_09_P074611 | 168,777 | 111,842 | 258,761 | 141,514 | 1,509 | 1,829 | 1,669 | CG7966 | - |
| A_09_P028426 | 348,399 | 236,869 | 427,795 | 298,627 | 1,471 | 1,433 | 1,452 | CG7971 | - |
| A_09_P065226 | 2262,734 | 1161,802 | 3032,011 | 1803,188 | 1,948 | 1,681 | 1,815 | CG7985 | - |
| A_09_P073041 | 238,381 | 128,382 | 459,738 | 279,869 | 1,857 | 1,643 | 1,750 | CG8176 | - |
| A_09_P053726 | 4688,845 | 2014,327 | 4753,062 | 2716,030 | 2,328 | 1,750 | 2,039 | CG8209 | - |
| A_09_P006721 | 764,645 | 445,526 | 1623,388 | 842,392 | 1,716 | 1,927 | 1,822 | CG8858 | - |
| A_09_P072946 | 200,414 | 129,094 | 517,212 | 290,880 | 1,552 | 1,778 | 1,665 | CG8861 | - |
| A_09_P196775 | 206,344 | 40,921 | 140,138 | 41,947 | 5,042 | 3,341 | 4,192 | CG9121 | - |
| A_09_P182930 | 258,273 | 76,688 | 266,550 | 120,032 | 3,368 | 2,221 | 2,794 | CG9135 | - |
| A_09_P023951 | 15527,263 | 9783,255 | 20384,348 | 13713,680 | 1,587 | 1,486 | 1,537 | CG9253 | - |
| A_09_P074801 | 76,215 | 33,630 | 117,313 | 62,297 | 2,266 | 1,883 | 2,075 | CG9269 | - |
| A_09_P073091 | 31,386 | 7,680 | 48,396 | 14,826 | 4,087 | 3,264 | 3,675 | CG9396 | - |
| A_09_P024546 | 37,574 | 7,588 | 50,363 | 7,014 | 4,952 | 7,180 | 6,066 | CG9447 | - |
| A_09_P210305 | 47,114 | 19,692 | 68,766 | 35,149 | 2,393 | 1,956 | 2,174 | CG9447 | - |
| A_09_P075586 | 115,281 | 59,913 | 170,104 | 101,167 | 1,924 | 1,681 | 1,803 | CG9593 | - |
| A_09_P221665 | 427,671 | 98,378 | 655,266 | 195,267 | 4,347 | 3,356 | 3,851 | CHES-1-like | Checkpoint suppressor 1-like |
| A_09_P209160 | 90,816 | 43,943 | 89,127 | 21,947 | 2,067 | 4,061 | 3,064 | chinmo | Chronologically inappropriate morphogenesis |
| A_09_P056986 | 2115,214 | 1209,324 | 4051,616 | 1635,472 | 1,749 | 2,477 | 2,113 | CHKov1 | CHK domain ov1 |
| A_09_P037256 | 261,978 | 169,935 | 995,571 | 259,689 | 1,542 | 3,834 | 2,688 | Cht6 | Chitinase 6 |
| A_09_P222795 | 505,435 | 330,911 | 1983,981 | 528,617 | 1,527 | 3,753 | 2,640 | Cht6 | Chitinase 6 |
| A_09_P037266 | 256,564 | 166,474 | 718,382 | 380,536 | 1,541 | 1,888 | 1,714 | Cht6 | Chitinase 6 |
| A_09_P079756 | 2019,905 | 1284,377 | 3598,281 | 2346,976 | 1,573 | 1,533 | 1,553 | cindr | CIN85 and CD2AP related |
| A_09_P051066 | 961,421 | 301,979 | 1025,719 | 350,639 | 3,184 | 2,925 | 3,055 | CLIP-190 | Cytoplasmic linker protein 190 |
| A_09_P049926 | 7201,479 | 4159,848 | 12040,413 | 7040,894 | 1,731 | 1,710 | 1,721 | cnc | cap-n-collar |
| A_09_P192595 | 2620,498 | 1489,685 | 4487,499 | 2500,641 | 1,759 | 1,795 | 1,777 | comm | commissureless |
| A_09_P015421 | 4163,983 | 2188,286 | 6704,175 | 2767,106 | 1,903 | 2,423 | 2,163 | corn | cornetto |
| A_09_P041796 | 530,597 | 119,381 | 595,807 | 208,653 | 4,445 | 2,855 | 3,650 | Cp38 | Chorion protein 38 |
| A_09_P030336 | 29,826 | 9,128 | 108,583 | 40,684 | 3,267 | 2,669 | 2,968 | Cp7Fa | Chorion protein a at 7F |
| A_09_P113905 | 210,929 | 126,475 | 209,506 | 133,303 | 1,668 | 1,572 | 1,620 | Cpr64Ac | Cuticular protein 64Ac |
| A_09_P144470 | 326274,200 | 193607,920 | 582476,440 | 365006,300 | 1,685 | 1,596 | 1,641 | CR40959 | - |
| A_09_P171105 | 863,030 | 117,005 | 460,898 | 100,202 | 7,376 | 4,600 | 5,988 | Csk | C-terminal Src kinase |
| A_09_P210820 | 370,086 | 67,530 | 71,253 | 9,568 | 5,480 | 7,447 | 6,464 | cv-c | crossveinless c |
| A_09_P006966 | 98,765 | 60,302 | 148,683 | 90,106 | 1,638 | 1,650 | 1,644 | Cyp301a1 | Cyp301a1 |
| A_09_P040201 | 79,157 | 35,952 | 46,897 | 14,274 | 2,202 | 3,286 | 2,744 | Cyp308a1 | Cyp308a1 |
| A_09_P132155 | 1036,644 | 68,126 | 1236,215 | 102,917 | 15,217 | 12,012 | 13,614 | Cyp6a20 | Cyp6a20 |
| A_09_P007841 | 1079,016 | 188,541 | 1111,836 | 191,028 | 5,723 | 5,820 | 5,772 | Cyp6a20 | Cyp6a20 |
| A_09_P007846 | 180,706 | 42,748 | 172,816 | 52,377 | 4,227 | 3,299 | 3,763 | Cyp6a21 | Cyp6a21 |
| A_09_P029906 | 77,837 | 41,496 | 57,911 | 23,755 | 1,876 | 2,438 | 2,157 | Cyp6a9 | Cytochrome P450-6a9 |
| A_09_P074906 | 626,542 | 397,519 | 1007,115 | 555,195 | 1,576 | 1,814 | 1,695 | Cyp6d5 | Cyp6d5 |
| A_09_P060886 | 788,145 | 350,186 | 1142,314 | 499,104 | 2,251 | 2,289 | 2,270 | Cyt-c1L | Cytochrome c1-like |
| A_09_P012021 | 313,554 | 125,016 | 423,285 | 251,783 | 2,508 | 1,681 | 2,095 | Dbp45A | DEAD box protein 45A |
| A_09_P001686 | 22622,941 | 3165,431 | 44015,414 | 12653,934 | 7,147 | 3,478 | 5,313 | Dbp80 | Dead box protein 80 |
| A_09_P002166 | 1628,316 | 731,708 | 2017,282 | 1237,346 | 2,225 | 1,630 | 1,928 | dbr | debra |
| A_09_P028136 | 716,878 | 272,078 | 677,752 | 421,822 | 2,635 | 1,607 | 2,121 | Dci | Dodecenoyl-CoA delta-isomerase |
| A_09_P177440 | 13659,378 | 5811,374 | 12547,980 | 7996,546 | 2,350 | 1,569 | 1,960 | Dgp-1 | Dgp-1 |
| A_09_P020876 | 1792,372 | 898,771 | 3480,451 | 2053,775 | 1,994 | 1,695 | 1,844 | Dh31 | Diuretic hormone 31 |
| A_09_P006931 | 45,946 | 19,539 | 60,973 | 25,329 | 2,352 | 2,407 | 2,379 | Dh44-R2 | Diuretic hormone 44 receptor 2 |
| A_09_P006936 | 680,958 | 374,270 | 816,247 | 545,992 | 1,819 | 1,495 | 1,657 | Dh44-R2 | Diuretic hormone 44 receptor 2 |
| A_09_P170540 | 512,680 | 148,228 | 578,073 | 199,576 | 3,459 | 2,897 | 3,178 | dj | don juan |
| A_09_P103295 | 2634,072 | 1645,185 | 4232,369 | 2607,343 | 1,601 | 1,623 | 1,612 | Dlg5 | Discs large 5 |
| A_09_P069766 | 175,064 | 97,010 | 208,837 | 121,198 | 1,805 | 1,723 | 1,764 | dmrt93B | doublesex-Mab related 93B |
| A_09_P012651 | 3230,046 | 1713,063 | 3101,317 | 2298,364 | 1,886 | 1,349 | 1,617 | dock | dreadlocks |
| A_09_P042056 | 1282,255 | 721,096 | 2322,206 | 1379,813 | 1,778 | 1,683 | 1,731 | dpp | decapentaplegic |
| A_09_P220910 | 1431,676 | 545,443 | 2061,658 | 901,960 | 2,625 | 2,286 | 2,455 | dpr6 | defective proboscis extension response 6 |
| A_09_P063661 | 1717,385 | 949,429 | 3209,444 | 1511,598 | 1,809 | 2,123 | 1,966 | dpr6 | defective proboscis extension response 6 |
| A_09_P190075 | 1921,690 | 1068,465 | 4053,917 | 1962,602 | 1,799 | 2,066 | 1,932 | dpr6 | defective proboscis extension response 6 |
| A_09_P177975 | 182,166 | 113,147 | 316,230 | 201,027 | 1,610 | 1,573 | 1,592 | dpr6 | defective proboscis extension response 6 |
| A_09_P017076 | 923,735 | 596,428 | 2021,629 | 1090,247 | 1,549 | 1,854 | 1,702 | dpy | dumpy |
| A_09_P032206 | 2426,231 | 1195,554 | 3490,709 | 2149,961 | 2,029 | 1,624 | 1,826 | drongo | drongo |
| A_09_P103420 | 153,635 | 65,923 | 192,973 | 119,202 | 2,331 | 1,619 | 1,975 | Dscam2 | Down syndrome cell adhesion molecule 2 |
| A_09_P047676 | 336,690 | 188,274 | 453,089 | 210,804 | 1,788 | 2,149 | 1,969 | Dscam2 | Down syndrome cell adhesion molecule 2 |
| A_09_P015411 | 349,702 | 206,747 | 477,190 | 310,310 | 1,691 | 1,538 | 1,615 | Dscam2 | Down syndrome cell adhesion molecule 2 |
| A_09_P029371 | 138469,660 | 47233,664 | 165101,810 | 82822,664 | 2,932 | 1,993 | 2,463 | Dsp1 | Dorsal switch protein 1 |
| A_09_P010656 | 9726,027 | 2403,574 | 10549,125 | 3829,625 | 4,046 | 2,755 | 3,401 | dy | dusky |
| A_09_P031711 | 190,119 | 138,490 | 398,803 | 253,386 | 1,373 | 1,574 | 1,473 | Dyrk2 | Dual-specificity tyrosine phosphorylation-regulated kinase 2 |
| A_09_P041261 | 179,649 | 29,692 | 937,661 | 98,773 | 6,050 | 9,493 | 7,772 | dyw | daywake |
| A_09_P047106 | 3438,172 | 1179,217 | 3467,433 | 1669,758 | 2,916 | 2,077 | 2,496 | E(bx) | Enhancer of bithorax |
| A_09_P043566 | 2095,465 | 1462,234 | 5023,495 | 2474,662 | 1,433 | 2,030 | 1,732 | E(spl)mgamma-HLH | Enhancer of split mgamma, helix-loop-helix |
| A_09_P103180 | 141,078 | 76,938 | 212,464 | 138,896 | 1,834 | 1,530 | 1,682 | e(y)3 | enhancer of yellow 3 |
| A_09_P203210 | 850,317 | 167,445 | 183,134 | 60,960 | 5,078 | 3,004 | 4,041 | ect | ectodermal |
| A_09_P204870 | 40,671 | 10,078 | 54,193 | 28,469 | 4,036 | 1,904 | 2,970 | Ect4 | Ectoderm-expressed 4 |
| A_09_P160005 | 1446,259 | 775,181 | 1752,868 | 798,246 | 1,866 | 2,196 | 2,031 | Ect4 | Ectoderm-expressed 4 |
| A_09_P217320 | 584,787 | 287,813 | 449,034 | 259,252 | 2,032 | 1,732 | 1,882 | Ect4 | Ectoderm-expressed 4 |
| A_09_P050391 | 1535,155 | 969,541 | 2138,807 | 1335,152 | 1,583 | 1,602 | 1,593 | Eip74EF | Ecdysone-induced protein 74EF |
| A_09_P011171 | 83,596 | 41,286 | 121,737 | 76,437 | 2,025 | 1,593 | 1,809 | Eip78C | Ecdysone-induced protein 78C |
| A_09_P029956 | 902,887 | 512,274 | 1470,939 | 827,432 | 1,763 | 1,778 | 1,770 | Eip93F | Ecdysone-induced protein 93F |
| A_09_P144045 | 4413,843 | 1313,669 | 4734,157 | 1761,230 | 3,360 | 2,688 | 3,024 | elm | ethanol sensitive with low memory |
| A_09_P036466 | 7745,009 | 2553,753 | 8578,236 | 3564,614 | 3,033 | 2,406 | 2,720 | elm | ethanol sensitive with low memory |
| A_09_P202805 | 4475,977 | 544,763 | 5026,677 | 1041,242 | 8,216 | 4,828 | 6,522 | ems | empty spiracles |
| A_09_P049201 | 39,360 | 8,641 | 63,084 | 15,027 | 4,555 | 4,198 | 4,377 | Esyt2 | Extended synaptotagmin-like protein 2 |
| A_09_P069876 | 372,303 | 240,124 | 590,103 | 378,817 | 1,550 | 1,558 | 1,554 | ETHR | ETHR |
| A_09_P010756 | 83,715 | 41,033 | 187,386 | 101,948 | 2,040 | 1,838 | 1,939 | ex | expanded |
| A_09_P070066 | 193,406 | 89,405 | 258,029 | 132,747 | 2,163 | 1,944 | 2,054 | Fadd | Fas-associated death domain |
| A_09_P025351 | 10430,594 | 2566,486 | 2546,727 | 1056,792 | 4,064 | 2,410 | 3,237 | FANCI | Fanconi anemia complementation group I |
| A_09_P042346 | 4168,295 | 2133,694 | 8089,615 | 4870,262 | 1,954 | 1,661 | 1,807 | Fas3 | Fasciclin 3 |
| A_09_P064696 | 62,768 | 32,570 | 99,481 | 55,572 | 1,927 | 1,790 | 1,859 | FASN2 | Fatty acid synthase 2 |
| A_09_P204435 | 197,245 | 24,319 | 278,047 | 109,475 | 8,111 | 2,540 | 5,325 | Fem-1 | Fem-1 |
| A_09_P212270 | 10376,601 | 1670,799 | 6274,907 | 1643,812 | 6,211 | 3,817 | 5,014 | fend | forked end |
| A_09_P060931 | 47,221 | 8,462 | 115,637 | 46,996 | 5,581 | 2,461 | 4,021 | FipoQ | F-box involved in polyQ pathogenesis |
| A_09_P033196 | 3251,370 | 1518,095 | 2717,179 | 1686,554 | 2,142 | 1,611 | 1,876 | fray | frayed |
| A_09_P073141 | 24,426 | 6,146 | 57,571 | 27,722 | 3,974 | 2,077 | 3,026 | Fst | Frost |
| A_09_P076401 | 81,453 | 46,325 | 184,186 | 104,448 | 1,758 | 1,763 | 1,761 | fus | fusilli |
| A_09_P010676 | 502,326 | 319,830 | 729,686 | 469,750 | 1,571 | 1,553 | 1,562 | Gad1 | Glutamic acid decarboxylase 1 |
| A_09_P030216 | 368,812 | 206,449 | 606,956 | 326,584 | 1,786 | 1,858 | 1,822 | gcm | glial cells missing |
| A_09_P069151 | 1738,967 | 350,683 | 2559,787 | 477,491 | 4,959 | 5,361 | 5,160 | Gdn1 | Gdown1 |
| A_09_P057696 | 869,291 | 379,336 | 1692,951 | 659,186 | 2,292 | 2,568 | 2,430 | GEFmeso | Guanine nucleotide exchange factor in mesoderm |
| A_09_P012966 | 290,133 | 92,588 | 131,261 | 70,804 | 3,134 | 1,854 | 2,494 | gish | gilgamesh |
| A_09_P012961 | 3925,079 | 2583,332 | 6999,791 | 4201,517 | 1,519 | 1,666 | 1,593 | gish | gilgamesh |
| A_09_P182735 | 2914,871 | 1849,197 | 4591,541 | 2910,560 | 1,576 | 1,578 | 1,577 | gish | gilgamesh |
| A_09_P030581 | 8640,132 | 5467,906 | 11520,522 | 7130,669 | 1,580 | 1,616 | 1,598 | glec | gliolectin |
| A_09_P225425 | 132,955 | 81,221 | 189,020 | 90,845 | 1,637 | 2,081 | 1,859 | Glut1 | Glucose transporter 1 |
| A_09_P056936 | 354,036 | 40,409 | 627,719 | 136,326 | 8,761 | 4,605 | 6,683 | Gr22e | Gustatory receptor 22e |
| A_09_P064156 | 119,346 | 78,010 | 181,622 | 110,126 | 1,530 | 1,649 | 1,590 | Gr59e | Gustatory receptor 59e |
| A_09_P053881 | 88,528 | 29,098 | 107,049 | 54,972 | 3,042 | 1,947 | 2,495 | Gr66a | Gustatory receptor 66a |
| A_09_P056841 | 73,072 | 21,686 | 138,136 | 61,744 | 3,370 | 2,237 | 2,803 | Gr93d | Gustatory receptor 93d |
| A_09_P209530 | 962,963 | 611,825 | 1738,665 | 839,122 | 1,574 | 2,072 | 1,823 | GstD10 | Glutathione S transferase D10 |
| A_09_P147905 | 102457,945 | 60298,965 | 104640,320 | 60375,785 | 1,699 | 1,733 | 1,716 | GstD2 | Glutathione S transferase D2 |
| A_09_P011881 | 98939,650 | 55995,996 | 98316,240 | 63780,883 | 1,767 | 1,541 | 1,654 | GstD2 | Glutathione S transferase D2 |
| A_09_P011911 | 3497,541 | 778,396 | 2530,121 | 715,494 | 4,493 | 3,536 | 4,015 | GstD8 | Glutathione S transferase D8 |
| A_09_P042656 | 2557,845 | 1370,308 | 5635,132 | 1954,323 | 1,867 | 2,883 | 2,375 | h | hairy |
| A_09_P172120 | 181,753 | 81,192 | 147,765 | 82,842 | 2,239 | 1,784 | 2,011 | h | hairy |
| A_09_P191595 | 1189,562 | 688,854 | 1914,158 | 1060,728 | 1,727 | 1,805 | 1,766 | hdc | headcase |
| A_09_P059601 | 271,216 | 101,312 | 566,637 | 195,074 | 2,677 | 2,905 | 2,791 | HEATR2 | HEAT repeat containing 2 |
| A_09_P209780 | 111,104 | 69,269 | 95,253 | 52,066 | 1,604 | 1,829 | 1,717 | Hel89B | Helicase 89B |
| A_09_P203410 | 658,293 | 115,139 | 124,344 | 34,068 | 5,717 | 3,650 | 4,684 | Hex-A | Hexokinase A |
| A_09_P010911 | 61,265 | 28,661 | 102,062 | 57,866 | 2,138 | 1,764 | 1,951 | hh | hedgehog |
| A_09_P204610 | 82,027 | 38,199 | 123,139 | 72,241 | 2,147 | 1,705 | 1,926 | hig | hikaru genki |
| A_09_P173980 | 4035,773 | 1804,070 | 8818,820 | 4048,165 | 2,237 | 2,178 | 2,208 | Hipk | Homeodomain interacting protein kinase |
| A_09_P028001 | 11713,715 | 7256,921 | 19857,040 | 11312,585 | 1,614 | 1,755 | 1,685 | Hipk | Homeodomain interacting protein kinase |
| A_09_P206450 | 64,587 | 32,995 | 266,583 | 63,528 | 1,957 | 4,196 | 3,077 | how | held out wings |
| A_09_P052411 | 8158,689 | 3221,736 | 9615,777 | 5888,695 | 2,532 | 1,633 | 2,083 | Hrb87F | Heterogeneous nuclear ribonucleoprotein at 87F |
| A_09_P136085 | 23567,770 | 11742,206 | 37258,050 | 20972,229 | 2,007 | 1,777 | 1,892 | Hrb98DE | Heterogeneous nuclear ribonucleoprotein at 98DE |
| A_09_P049066 | 106,008 | 38,417 | 89,196 | 33,380 | 2,759 | 2,672 | 2,716 | Hs3st-A | Heparan sulfate 3-O sulfotransferase-A |
| A_09_P042786 | 151308,580 | 74967,680 | 235524,890 | 141744,030 | 2,018 | 1,662 | 1,840 | Hsp22 | Heat shock protein 22 |
| A_09_P022666 | 40,407 | 15,594 | 62,505 | 31,986 | 2,591 | 1,954 | 2,273 | Hsp60D | Heat shock protein 60D |
| A_09_P029581 | 29342,960 | 18413,620 | 58153,240 | 26789,945 | 1,594 | 2,171 | 1,882 | Hsp70Bb | Heat-shock-protein-70Bb |
| A_09_P206666 | 62,170 | 8,443 | 91,205 | 20,757 | 7,364 | 4,394 | 5,879 | Hydr1 | alpha/beta hydrolase 1 |
| A_09_P103360 | 53134,770 | 13094,418 | 20607,857 | 7260,136 | 4,058 | 2,838 | 3,448 | IA-2 | IA-2 protein tyrosine phosphatase |
| A_09_P069811 | 204,171 | 73,985 | 339,039 | 142,698 | 2,760 | 2,376 | 2,568 | Ice2 | Interacts with the C terminus of ELL 2 |
| A_09_P032346 | 180,386 | 94,325 | 429,710 | 157,818 | 1,912 | 2,723 | 2,318 | Idgf3 | Imaginal disc growth factor 3 |
| A_09_P017016 | 164,235 | 88,206 | 133,379 | 100,771 | 1,862 | 1,324 | 1,593 | inaE | inactivation no afterpotential E |
| A_09_P145775 | 79,112 | 23,247 | 32,669 | 5,946 | 3,403 | 5,494 | 4,449 | Inx2 | Innexin 2 |
| A_09_P002696 | 22,340 | 5,987 | 45,035 | 17,865 | 3,731 | 2,521 | 3,126 | Ir10a | Ionotropic receptor 10a |
| A_09_P008001 | 35,841 | 8,667 | 64,408 | 15,802 | 4,135 | 4,076 | 4,106 | Ir52a | Ionotropic receptor 52a |
| A_09_P034876 | 17112,594 | 4879,113 | 21416,875 | 7694,392 | 3,507 | 2,783 | 3,145 | Ir76b | Ionotropic receptor 76b |
| A_09_P203330 | 214,750 | 121,605 | 353,643 | 135,048 | 1,766 | 2,619 | 2,192 | Irk1 | Inwardly rectifying potassium channel 1 |
| A_09_P014521 | 169,478 | 92,918 | 232,041 | 163,640 | 1,824 | 1,418 | 1,621 | IRSp53 | Insulin receptor substrate 53 kDa |
| A_09_P109975 | 22641,299 | 8352,295 | 40539,620 | 13444,517 | 2,711 | 3,015 | 2,863 | isoQC | iso Glutaminyl cyclase |
| A_09_P001041 | 129,217 | 11,460 | 23,949 | 7,699 | 11,275 | 3,111 | 7,193 | Jabba | Jabba |
| A_09_P009331 | 8745,182 | 2352,693 | 9260,413 | 3350,014 | 3,717 | 2,764 | 3,241 | Jheh3 | Juvenile hormone epoxide hydrolase 3 |
| A_09_P166565 | 639,470 | 197,601 | 821,524 | 207,154 | 3,236 | 3,966 | 3,601 | jim | jim |
| A_09_P079226 | 1411,056 | 822,402 | 2391,934 | 1519,046 | 1,716 | 1,575 | 1,645 | jim | jim |
| A_09_P015451 | 128,218 | 34,550 | 233,263 | 69,029 | 3,711 | 3,379 | 3,545 | jv | javelin |
| A_09_P178885 | 61,255 | 24,719 | 93,337 | 38,111 | 2,478 | 2,449 | 2,464 | Kat60 | Katanin 60 |
| A_09_P032941 | 7676,296 | 2980,753 | 10942,798 | 3748,657 | 2,575 | 2,919 | 2,747 | KdelR | KDEL receptor |
| A_09_P048566 | 3479,163 | 1519,688 | 2412,681 | 1218,201 | 2,289 | 1,981 | 2,135 | kdn | knockdown |
| A_09_P052191 | 783,210 | 445,840 | 855,167 | 488,655 | 1,757 | 1,750 | 1,753 | kek1 | kekkon 1 |
| A_09_P204710 | 435,177 | 34,244 | 336,917 | 53,810 | 12,708 | 6,261 | 9,485 | kek3 | kekkon 3 |
| A_09_P022486 | 189,952 | 66,325 | 232,977 | 153,057 | 2,864 | 1,522 | 2,193 | kek4 | kekkon 4 |
| A_09_P012941 | 1036,998 | 518,582 | 2742,413 | 1096,206 | 2,000 | 2,502 | 2,251 | ken | ken and barbie |
| A_09_P118005 | 559,032 | 326,829 | 1444,254 | 644,699 | 1,710 | 2,240 | 1,975 | ken | ken and barbie |
| A_09_P203930 | 234,520 | 148,730 | 1120,901 | 307,718 | 1,577 | 3,643 | 2,610 | kirre | kin of irre |
| A_09_P043016 | 23,699 | 6,204 | 54,616 | 5,978 | 3,820 | 9,136 | 6,478 | Kr | Kruppel |
| A_09_P146365 | 2666,586 | 1290,926 | 3983,147 | 2535,353 | 2,066 | 1,571 | 1,818 | KrT95D | Krueppel target at 95D |
| A_09_P199440 | 1750,883 | 787,583 | 2269,646 | 1359,383 | 2,223 | 1,670 | 1,946 | kuz | kuzbanian |
| A_09_P029691 | 324,502 | 182,099 | 590,305 | 280,048 | 1,782 | 2,108 | 1,945 | l(2)dtl | lethal-(2)-denticleless |
| A_09_P192425 | 194,216 | 28,691 | 336,149 | 127,108 | 6,769 | 2,645 | 4,707 | l(3)L1231 | lethal (3) L1231 |
| A_09_P137855 | 223,787 | 139,365 | 434,999 | 230,673 | 1,606 | 1,886 | 1,746 | Lasp | Lasp |
| A_09_P046656 | 141,967 | 18,088 | 157,150 | 44,426 | 7,849 | 3,537 | 5,693 | lbl | ladybird late |
| A_09_P032541 | 87,524 | 29,886 | 91,810 | 48,302 | 2,929 | 1,901 | 2,415 | Lcp65Ag2 | Lcp65Ag2 |
| A_09_P062346 | 72,836 | 16,229 | 103,459 | 62,257 | 4,488 | 1,662 | 3,075 | lectin-46Ca | lectin-46Ca |
| A_09_P007341 | 138,003 | 82,105 | 186,473 | 65,113 | 1,681 | 2,864 | 2,272 | link | link |
| A_09_P076441 | 68,903 | 11,900 | 42,308 | 22,382 | 5,790 | 1,890 | 3,840 | Lip3 | Lip3 |
| A_09_P006346 | 414,787 | 82,501 | 120,432 | 42,845 | 5,028 | 2,811 | 3,919 | Listericin | Listericin |
| A_09_P176845 | 32844,490 | 4340,188 | 76573,720 | 20110,428 | 7,568 | 3,808 | 5,688 | lncRNA:CR32218 | long non-coding RNA:CR32218 |
| A_09_P001151 | 74,996 | 7,259 | 76,243 | 46,526 | 10,332 | 1,639 | 5,985 | lncRNA:CR34044 | long non-coding RNA:CR34044 |
| A_09_P196695 | 48,999 | 18,853 | 77,495 | 5,907 | 2,599 | 13,119 | 7,859 | lncRNA:CR42719 | long non-coding RNA:CR42719 |
| A_09_P182310 | 94,641 | 7,358 | 86,863 | 21,276 | 12,862 | 4,083 | 8,473 | lncRNA:CR43242 | long non-coding RNA:CR43242 |
| A_09_P208735 | 455,659 | 198,201 | 568,994 | 218,153 | 2,299 | 2,608 | 2,454 | lncRNA:CR43242 | long non-coding RNA:CR43242 |
| A_09_P221405 | 1912,408 | 1082,963 | 2840,668 | 1003,081 | 1,766 | 2,832 | 2,299 | lncRNA:CR43459 | long non-coding RNA:CR43459 |
| A_09_P175380 | 375,995 | 250,045 | 624,555 | 301,792 | 1,504 | 2,069 | 1,787 | lncRNA:CR44068 | long non-coding RNA:CR44068 |
| A_09_P193395 | 223,893 | 82,640 | 276,534 | 180,027 | 2,709 | 1,536 | 2,123 | lncRNA:CR44922 | long non-coding RNA:CR44922 |
| A_09_P002906 | 625,947 | 155,084 | 733,994 | 258,436 | 4,036 | 2,840 | 3,438 | lncRNA:CR44997 | long non-coding RNA:CR44997 |
| A_09_P005376 | 659,090 | 440,199 | 1119,451 | 572,407 | 1,497 | 1,956 | 1,726 | lncRNA:CR45177 | long non-coding RNA:CR45177 |
| A_09_P110765 | 217,634 | 99,846 | 304,155 | 148,206 | 2,180 | 2,052 | 2,116 | Loxl1 | Lysyl oxidase-like 1 |
| A_09_P025071 | 4302,579 | 2625,188 | 7760,871 | 4510,797 | 1,639 | 1,721 | 1,680 | Lpin | Lipin |
| A_09_P219540 | 123,868 | 19,073 | 500,379 | 101,099 | 6,494 | 4,949 | 5,722 | Lrch | Leucine-rich-repeats and calponin homology domain protein |
| A_09_P208180 | 129,379 | 71,049 | 178,403 | 99,224 | 1,821 | 1,798 | 1,809 | Lrt | Leucine-rich tendon-specific protein |
| A_09_P205005 | 35,274 | 8,215 | 81,198 | 34,727 | 4,294 | 2,338 | 3,316 | Lsd-2 | Lipid storage droplet-2 |
| A_09_P051906 | 223,964 | 144,372 | 318,617 | 211,874 | 1,551 | 1,504 | 1,528 | mAChR-A | muscarinic Acetylcholine Receptor, A-type |
| A_09_P180765 | 2256,972 | 550,428 | 378,649 | 192,196 | 4,100 | 1,970 | 3,035 | Mbs | Myosin binding subunit |
| A_09_P071836 | 58,118 | 33,233 | 94,996 | 59,411 | 1,749 | 1,599 | 1,674 | Mco3 | Multicopper oxidase 3 |
| A_09_P034341 | 1633,624 | 876,992 | 2038,238 | 1350,013 | 1,863 | 1,510 | 1,686 | MED11 | Mediator complex subunit 11 |
| A_09_P198290 | 868,589 | 379,042 | 1475,242 | 684,952 | 2,292 | 2,154 | 2,223 | mei-38 | meiotic 38 |
| A_09_P161295 | 696,598 | 322,734 | 1065,504 | 465,410 | 2,158 | 2,289 | 2,224 | Meltrin | Meltrin |
| A_09_P192440 | 644,790 | 317,607 | 1002,019 | 425,156 | 2,030 | 2,357 | 2,193 | Meltrin | Meltrin |
| A_09_P162315 | 1538,537 | 839,906 | 2456,318 | 1043,202 | 1,832 | 2,355 | 2,093 | Meltrin | Meltrin |
| A_09_P172130 | 60,180 | 8,280 | 71,585 | 23,827 | 7,268 | 3,004 | 5,136 | Mhc | Myosin heavy chain |
| A_09_P195840 | 881,024 | 166,303 | 942,125 | 255,689 | 5,298 | 3,685 | 4,491 | Mink | Mitotic spindle and nuclear protein |
| A_09_P076466 | 956,305 | 357,368 | 5773,230 | 1583,599 | 2,676 | 3,646 | 3,161 | mip130 | Myb-interacting protein 130 |
| A_09_P043581 | 136,683 | 82,439 | 205,155 | 133,385 | 1,658 | 1,538 | 1,598 | Mlc1 | Myosin alkali light chain 1 |
| A_09_P224875 | 2920,987 | 738,802 | 862,767 | 271,787 | 3,954 | 3,174 | 3,564 | Mlc2 | Myosin light chain 2 |
| A_09_P182475 | 150,106 | 62,583 | 295,087 | 138,026 | 2,399 | 2,138 | 2,268 | mnd | minidiscs |
| A_09_P162535 | 32,648 | 5,906 | 48,333 | 26,716 | 5,528 | 1,809 | 3,669 | Mnt | Mnt |
| A_09_P016996 | 805,081 | 535,888 | 1133,950 | 689,852 | 1,502 | 1,644 | 1,573 | Mp | Multiplexin |
| A_09_P146950 | 125,990 | 76,840 | 267,276 | 158,235 | 1,640 | 1,689 | 1,664 | MrgBP | MRG/MORF4L binding protein |
| A_09_P126120 | 72,110 | 36,367 | 103,521 | 66,377 | 1,983 | 1,560 | 1,771 | mRpL15 | mitochondrial ribosomal protein L15 |
| A_09_P180150 | 115,133 | 46,752 | 183,853 | 115,416 | 2,463 | 1,593 | 2,028 | mRpS31 | mitochondrial ribosomal protein S31 |
| A_09_P188665 | 4388,642 | 2759,797 | 8001,257 | 4322,839 | 1,590 | 1,851 | 1,721 | mtgo | miles to go |
| A_09_P203800 | 188,164 | 119,240 | 272,798 | 97,201 | 1,578 | 2,807 | 2,192 | MtnA | Metallothionein A |
| A_09_P194615 | 1111,325 | 734,613 | 1332,412 | 717,978 | 1,513 | 1,856 | 1,684 | Muc68Ca | Mucin 68Ca |
| A_09_P101105 | 301,237 | 145,835 | 762,676 | 217,956 | 2,066 | 3,499 | 2,782 | mwh | multiple wing hairs |
| A_09_P028191 | 121,626 | 57,247 | 220,150 | 72,151 | 2,125 | 3,051 | 2,588 | mwh | multiple wing hairs |
| A_09_P174650 | 47,983 | 14,817 | 59,730 | 27,651 | 3,238 | 2,160 | 2,699 | mxt | mextli |
| A_09_P000231 | 266,508 | 124,287 | 418,872 | 187,267 | 2,144 | 2,237 | 2,191 | nab | nab |
| A_09_P223405 | 92,346 | 33,342 | 52,676 | 26,933 | 2,770 | 1,956 | 2,363 | nAChRalpha2 | nicotinic Acetylcholine Receptor alpha2 |
| A_09_P091020 | 73,549 | 30,078 | 124,866 | 72,986 | 2,445 | 1,711 | 2,078 | nAChRalpha6 | nicotinic Acetylcholine Receptor alpha6 |
| A_09_P169310 | 80,590 | 41,867 | 138,510 | 77,011 | 1,925 | 1,799 | 1,862 | nAChRalpha6 | nicotinic Acetylcholine Receptor alpha6 |
| A_09_P207700 | 24706,494 | 6192,372 | 26405,799 | 9741,283 | 3,990 | 2,711 | 3,350 | ND-13B | NADH dehydrogenase (ubiquinone) 13 kDa B subunit |
| A_09_P031786 | 673,556 | 234,589 | 982,247 | 401,848 | 2,871 | 2,444 | 2,658 | Nelf-E | Negative elongation factor E |
| A_09_P054701 | 589,932 | 264,783 | 629,472 | 408,090 | 2,228 | 1,542 | 1,885 | NijA | Ninjurin A |
| A_09_P135525 | 6103,533 | 3583,439 | 11266,115 | 6638,462 | 1,703 | 1,697 | 1,700 | Nmda1 | N-methyl-D-aspartate receptor-associated protein |
| A_09_P165885 | 1403,920 | 397,468 | 531,265 | 198,628 | 3,532 | 2,675 | 3,103 | nmo | nemo |
| A_09_P030266 | 474,690 | 315,778 | 1059,299 | 664,359 | 1,503 | 1,594 | 1,549 | nol | no optic lobe |
| A_09_P030041 | 769,367 | 163,894 | 1031,879 | 261,186 | 4,694 | 3,951 | 4,323 | Nsf2 | N-ethylmaleimide-sensitive factor 2 |
| A_09_P007686 | 16567,994 | 4563,810 | 21920,549 | 7466,649 | 3,630 | 2,936 | 3,283 | Oaz | O/E-associated zinc finger protein |
| A_09_P026621 | 95,194 | 52,046 | 119,985 | 50,901 | 1,829 | 2,357 | 2,093 | Obp58c | Odorant-binding protein 58c |
| A_09_P105570 | 1048,678 | 647,229 | 729,835 | 469,476 | 1,620 | 1,555 | 1,587 | Obp69a | Odorant-binding protein 69a |
| A_09_P010916 | 1225,885 | 799,199 | 2011,173 | 1208,636 | 1,534 | 1,664 | 1,599 | ogre | optic ganglion reduced |
| A_09_P064301 | 371,836 | 211,131 | 512,652 | 270,130 | 1,761 | 1,898 | 1,829 | olf186-F | olf186-F |
| A_09_P028871 | 72,769 | 15,543 | 83,376 | 30,444 | 4,682 | 2,739 | 3,710 | Or63a | Odorant receptor 63a |
| A_09_P053956 | 199,210 | 136,959 | 384,505 | 257,365 | 1,455 | 1,494 | 1,474 | Oseg1 | Outer segment 1 |
| A_09_P036651 | 137,966 | 50,290 | 390,983 | 147,937 | 2,743 | 2,643 | 2,693 | Osi1 | Osiris 1 |
| A_09_P185305 | 773,067 | 492,890 | 1894,149 | 639,763 | 1,568 | 2,961 | 2,265 | par-1 | par-1 |
| A_09_P054416 | 9714,037 | 3214,958 | 12034,779 | 5744,496 | 3,022 | 2,095 | 2,558 | path | pathetic |
| A_09_P064446 | 1057,917 | 369,042 | 1377,783 | 676,500 | 2,867 | 2,037 | 2,452 | Pax | Paxillin |
| A_09_P064441 | 379,044 | 228,651 | 526,212 | 334,007 | 1,658 | 1,575 | 1,617 | Pax | Paxillin |
| A_09_P222875 | 2263,205 | 574,164 | 2279,861 | 674,246 | 3,942 | 3,381 | 3,662 | Pcyt1 | Phosphocholine cytidylyltransferase 1 |
| A_09_P223085 | 104,773 | 60,736 | 160,773 | 106,152 | 1,725 | 1,515 | 1,620 | Pdcd4 | Programmed cell death 4 |
| A_09_P116730 | 1192,971 | 296,208 | 373,741 | 236,961 | 4,027 | 1,577 | 2,802 | Pde1c | Phosphodiesterase 1c |
| A_09_P117990 | 582,174 | 340,072 | 627,830 | 396,482 | 1,712 | 1,584 | 1,648 | Pdp1 | PAR-domain protein 1 |
| A_09_P054071 | 514,468 | 197,587 | 665,710 | 407,547 | 2,604 | 1,633 | 2,119 | Pex7 | Peroxin 7 |
| A_09_P129065 | 1359,648 | 707,657 | 2359,468 | 1119,915 | 1,921 | 2,107 | 2,014 | Pex7 | Peroxin 7 |
| A_09_P056566 | 40,907 | 6,553 | 58,728 | 17,685 | 6,242 | 3,321 | 4,781 | PGRP-SB1 | Peptidoglycan recognition protein SB1 |
| A_09_P111805 | 86,160 | 31,587 | 97,201 | 21,310 | 2,728 | 4,561 | 3,644 | PGRP-SB1 | Peptidoglycan recognition protein SB1 |
| A_09_P056576 | 399,466 | 122,594 | 343,414 | 125,931 | 3,258 | 2,727 | 2,993 | phu | phurba tashi |
| A_09_P215370 | 670,192 | 222,482 | 559,044 | 325,740 | 3,012 | 1,716 | 2,364 | pirk | poor Imd response upon knock-in |
| A_09_P026156 | 559,390 | 197,550 | 507,386 | 291,484 | 2,832 | 1,741 | 2,286 | pirk | poor Imd response upon knock-in |
| A_09_P207530 | 652,473 | 242,508 | 529,913 | 290,511 | 2,691 | 1,824 | 2,257 | pirk | poor Imd response upon knock-in |
| A_09_P038121 | 11279,647 | 1762,855 | 6738,618 | 1765,791 | 6,399 | 3,816 | 5,107 | Pits | Protein interacting with Ttk69 and Sin3A |
| A_09_P047511 | 53,526 | 16,229 | 99,335 | 26,853 | 3,298 | 3,699 | 3,499 | Pkcdelta | Protein kinase C delta |
| A_09_P223490 | 310,152 | 154,015 | 495,879 | 289,044 | 2,014 | 1,716 | 1,865 | PKD | Protein Kinase D |
| A_09_P183330 | 5501,636 | 1666,032 | 2042,335 | 814,135 | 3,302 | 2,509 | 2,905 | plx | pollux |
| A_09_P172175 | 7443,827 | 1764,908 | 13790,987 | 4929,052 | 4,218 | 2,798 | 3,508 | PNUTS | PNUTS |
| A_09_P218550 | 157,663 | 13,869 | 222,380 | 54,584 | 11,368 | 4,074 | 7,721 | pon | partner of numb |
| A_09_P058766 | 102,052 | 55,137 | 222,489 | 98,145 | 1,851 | 2,267 | 2,059 | Ppi1 | Protein phosphatase 1c interacting protein 1 |
| A_09_P039826 | 35,840 | 8,617 | 55,443 | 29,736 | 4,159 | 1,865 | 3,012 | ppk23 | pickpocket 23 |
| A_09_P011301 | 883,699 | 570,738 | 2172,302 | 1368,506 | 1,548 | 1,587 | 1,568 | Prat | Phosphoribosylamidotransferase |
| A_09_P046481 | 463022,440 | 240927,980 | 646356,900 | 404465,750 | 1,922 | 1,598 | 1,760 | pre-rRNA:CR45847 | ribosomal RNA primary transcript:CR45847 |
| A_09_P122045 | 135,920 | 15,127 | 39,201 | 14,953 | 8,986 | 2,622 | 5,804 | primo-1 | primo-1 |
| A_09_P149685 | 1955,129 | 491,789 | 2495,048 | 641,730 | 3,976 | 3,888 | 3,932 | Prm | Paramyosin |
| A_09_P044231 | 3186,101 | 806,669 | 3299,963 | 880,427 | 3,950 | 3,748 | 3,849 | Prm | Paramyosin |
| A_09_P213765 | 1129,302 | 692,928 | 2502,238 | 1293,404 | 1,630 | 1,935 | 1,782 | prom | prominin |
| A_09_P013146 | 680,093 | 290,277 | 814,186 | 513,554 | 2,343 | 1,585 | 1,964 | PR-Set7 | PR/SET domain containing protein 7 |
| A_09_P220875 | 165,346 | 77,828 | 270,651 | 144,999 | 2,125 | 1,867 | 1,996 | ptc | patched |
| A_09_P186330 | 41,900 | 6,112 | 38,567 | 16,621 | 6,855 | 2,320 | 4,588 | Ptp61F | Protein tyrosine phosphatase 61F |
| A_09_P052421 | 89,475 | 20,142 | 70,883 | 30,995 | 4,442 | 2,287 | 3,365 | pyd | polychaetoid |
| A_09_P005056 | 988,123 | 477,793 | 2053,618 | 1030,569 | 2,068 | 1,993 | 2,030 | Pzl | Piezo-like |
| A_09_P005331 | 822,247 | 518,863 | 1122,556 | 537,189 | 1,585 | 2,090 | 1,837 | Pzl | Piezo-like |
| A_09_P005066 | 114,614 | 70,926 | 97,262 | 67,300 | 1,616 | 1,445 | 1,531 | Pzl | Piezo-like |
| A_09_P198245 | 88,134 | 45,150 | 90,507 | 50,111 | 1,952 | 1,806 | 1,879 | Rab7 | Rab7 |
| A_09_P076416 | 530,621 | 357,517 | 1285,187 | 863,348 | 1,484 | 1,489 | 1,486 | Rbcn-3A | Rabconnectin-3A |
| A_09_P068346 | 702,766 | 454,236 | 1332,386 | 757,609 | 1,547 | 1,759 | 1,653 | Rbm13 | RNA-binding motif protein 13 |
| A_09_P196475 | 76,957 | 19,739 | 76,390 | 40,401 | 3,899 | 1,891 | 2,895 | r-cup | ryder cup |
| A_09_P040941 | 42,373 | 19,101 | 88,879 | 60,301 | 2,218 | 1,474 | 1,846 | r-cup | ryder cup |
| A_09_P051346 | 1932,952 | 999,867 | 3431,386 | 1966,315 | 1,933 | 1,745 | 1,839 | rdx | roadkill |
| A_09_P189010 | 87,375 | 25,303 | 147,023 | 40,179 | 3,453 | 3,659 | 3,556 | Reck | Reversion-inducing-cysteine-rich protein with kazal motifs |
| A_09_P218525 | 87,241 | 33,925 | 126,441 | 68,074 | 2,572 | 1,857 | 2,214 | Rgk1 | Rad, Gem/Kir family member 1 |
| A_09_P078481 | 2235,658 | 1192,321 | 3784,700 | 1767,942 | 1,875 | 2,141 | 2,008 | Rgl | Ral guanine nucleotide dissociation stimulator-like |
| A_09_P033901 | 56063,105 | 18166,713 | 43941,890 | 17906,133 | 3,086 | 2,454 | 2,770 | rogdi | rogdi |
| A_09_P191960 | 571,501 | 369,195 | 830,523 | 435,856 | 1,548 | 1,906 | 1,727 | Rox8 | Rox8 |
| A_09_P145095 | 327,929 | 211,691 | 553,842 | 285,143 | 1,549 | 1,942 | 1,746 | RpL7 | Ribosomal protein L7 |
| A_09_P150000 | 304,176 | 113,869 | 589,723 | 178,677 | 2,671 | 3,300 | 2,986 | RpLP1 | Ribosomal protein LP1 |
| A_09_P203270 | 5807,111 | 2612,350 | 6629,565 | 3653,962 | 2,223 | 1,814 | 2,019 | RpS21 | Ribosomal protein S21 |
| A_09_P044496 | 5463,379 | 2888,879 | 7211,368 | 4333,514 | 1,891 | 1,664 | 1,778 | ru | roughoid |
| A_09_P016126 | 22805,236 | 6154,619 | 6063,989 | 2479,532 | 3,705 | 2,446 | 3,076 | rudhira | rudhira |
| A_09_P011111 | 29,475 | 5,749 | 70,579 | 31,834 | 5,127 | 2,217 | 3,672 | RYa-R | RYamide receptor |
| A_09_P048956 | 1408,089 | 400,990 | 2799,617 | 956,774 | 3,512 | 2,926 | 3,219 | S | Star |
| A_09_P132165 | 3327,993 | 1572,12 | 7678,367 | 1791,276 | 2,117 | 4,287 | 3,202 | scb | scab |
| A_09_P032656 | 543,982 | 172,496 | 1295,499 | 492,842 | 3,154 | 2,629 | 2,891 | Scp2 | Sarcoplasmic calcium-binding protein 2 |
| A_09_P058931 | 47,563 | 6,816 | 38,885 | 11,954 | 6,978 | 3,253 | 5,116 | scrib | scribble |
| A_09_P078221 | 3686,928 | 1020,151 | 7788,675 | 1177,690 | 3,614 | 6,614 | 5,114 | scrib | scribble |
| A_09_P103000 | 1516,412 | 348,036 | 2918,270 | 673,445 | 4,357 | 4,333 | 4,345 | scrib | scribble |
| A_09_P078226 | 2274,195 | 610,084 | 3165,843 | 914,922 | 3,728 | 3,460 | 3,594 | scrib | scribble |
| A_09_P078231 | 1472,777 | 441,562 | 2020,592 | 559,607 | 3,335 | 3,611 | 3,473 | scrib | scribble |
| A_09_P215580 | 766,641 | 260,150 | 1206,158 | 366,441 | 2,947 | 3,292 | 3,119 | scrib | scribble |
| A_09_P071996 | 988,925 | 335,275 | 1838,162 | 585,228 | 2,950 | 3,141 | 3,045 | scrib | scribble |
| A_09_P103855 | 494,096 | 222,768 | 692,265 | 224,232 | 2,218 | 3,087 | 2,653 | scrib | scribble |
| A_09_P162590 | 165,504 | 100,220 | 288,586 | 161,998 | 1,651 | 1,781 | 1,716 | scrib | scribble |
| A_09_P010266 | 235,506 | 151,853 | 671,127 | 297,371 | 1,551 | 2,257 | 1,904 | Ser | Serrate |
| A_09_P173610 | 30,407 | 7,736 | 35,081 | 16,967 | 3,930 | 2,068 | 2,999 | Sfp26Ad | Seminal fluid protein 26Ad |
| A_09_P044641 | 146,215 | 83,847 | 277,469 | 139,297 | 1,744 | 1,992 | 1,868 | Sgs3 | Salivary gland secretion 3 |
| A_09_P186535 | 500,754 | 301,035 | 7369,018 | 1822,529 | 1,663 | 4,043 | 2,853 | Sh | Shaker |
| A_09_P183095 | 837,606 | 131,649 | 473,142 | 109,373 | 6,362 | 4,326 | 5,344 | shep | alan shepard |
| A_09_P044726 | 779,145 | 408,734 | 1503,629 | 847,897 | 1,906 | 1,773 | 1,840 | shn | schnurri |
| A_09_P047796 | 124,290 | 54,912 | 142,207 | 66,871 | 2,263 | 2,127 | 2,195 | side-III | sidestep III |
| A_09_P219160 | 15845,726 | 1981,673 | 43121,824 | 11055,027 | 7,996 | 3,901 | 5,948 | side-IV | sidestep IV |
| A_09_P190555 | 722,894 | 342,644 | 1273,102 | 765,034 | 2,110 | 1,664 | 1,887 | side-V | sidestep V |
| A_09_P107810 | 1008,184 | 649,991 | 1796,576 | 994,776 | 1,551 | 1,806 | 1,679 | skd | skuld |
| A_09_P050176 | 316,322 | 204,984 | 780,776 | 474,446 | 1,543 | 1,646 | 1,594 | skd | skuld |
| A_09_P118180 | 3063,502 | 1145,415 | 5599,396 | 2430,038 | 2,675 | 2,304 | 2,489 | sktl | skittles |
| A_09_P027451 | 5103,682 | 3068,011 | 8733,102 | 4044,367 | 1,664 | 2,159 | 1,911 | Slik | Sterile20-like kinase |
| A_09_P003971 | 441,609 | 156,242 | 1019,393 | 578,523 | 2,826 | 1,762 | 2,294 | SLO2 | slowpoke 2 |
| A_09_P220695 | 147,029 | 28,681 | 82,183 | 17,439 | 5,126 | 4,713 | 4,919 | sls | sallimus |
| A_09_P203015 | 3620,004 | 1938,041 | 7272,774 | 3381,659 | 1,868 | 2,151 | 2,009 | Smg5 | Smg5 |
| A_09_P125685 | 134,891 | 25,952 | 113,604 | 34,458 | 5,198 | 3,297 | 4,247 | smp-30 | Senescence marker protein-30 |
| A_09_P008571 | 211,711 | 40,060 | 544,963 | 24,055 | 5,285 | 22,655 | 13,970 | SmydA-6 | SET and MYND domain containing, arthropod-specific, member 6 |
| A_09_P163240 | 870,488 | 228,670 | 1251,132 | 349,081 | 3,807 | 3,584 | 3,695 | SNF4Agamma | SNF4/AMP-activated protein kinase gamma subunit |
| A_09_P018686 | 5574,744 | 3482,665 | 7618,788 | 2748,829 | 1,601 | 2,772 | 2,186 | Snx21 | Sorting nexin 21 |
| A_09_P045976 | 11756,427 | 7256,605 | 18995,992 | 11179,332 | 1,620 | 1,699 | 1,660 | Socs36E | Suppressor of cytokine signaling at 36E |
| A_09_P090255 | 298,137 | 121,764 | 454,441 | 180,691 | 2,448 | 2,515 | 2,482 | sowah | sosondowah |
| A_09_P168010 | 55,382 | 14,356 | 57,134 | 22,840 | 3,858 | 2,501 | 3,180 | SP1029 | SP1029 |
| A_09_P045541 | 45,034 | 18,677 | 286,025 | 146,078 | 2,411 | 1,958 | 2,185 | SPARC | Secreted protein, acidic, cysteine-rich |
| A_09_P044851 | 4296,976 | 2095,405 | 6972,599 | 3641,759 | 2,051 | 1,915 | 1,983 | spir | spire |
| A_09_P103090 | 1784,742 | 1013,079 | 2321,679 | 1453,230 | 1,762 | 1,598 | 1,680 | Spn | Spinophilin |
| A_09_P048671 | 767,879 | 513,275 | 1333,490 | 819,300 | 1,496 | 1,628 | 1,562 | spn-F | spn-F |
| A_09_P214100 | 568,868 | 242,822 | 676,008 | 331,125 | 2,343 | 2,042 | 2,192 | stac | staccato |
| A_09_P017276 | 222,524 | 109,022 | 367,838 | 180,051 | 2,041 | 2,043 | 2,042 | Ste:CG33245 | Ste:CG33245 |
| A_09_P211925 | 1164,230 | 759,493 | 2695,752 | 601,795 | 1,533 | 4,480 | 3,006 | stet | stem cell tumor |
| A_09_P065441 | 1476,190 | 152,728 | 4572,060 | 283,837 | 9,665 | 16,108 | 12,887 | sut2 | sugar transporter 2 |
| A_09_P177240 | 1702,271 | 215,444 | 1122,005 | 239,474 | 7,901 | 4,685 | 6,293 | Svil | Supervillin |
| A_09_P150720 | 266,990 | 126,691 | 507,030 | 259,809 | 2,107 | 1,952 | 2,029 | Svil | Supervillin |
| A_09_P028741 | 193,637 | 151,462 | 314,404 | 224,084 | 1,278 | 1,403 | 1,341 | Svil | Supervillin |
| A_09_P049131 | 185,395 | 103,001 | 302,039 | 184,571 | 1,800 | 1,636 | 1,718 | svp | seven up |
| A_09_P177215 | 12416,950 | 7757,686 | 24212,463 | 13722,503 | 1,601 | 1,764 | 1,683 | Syp | Syncrip |
| A_09_P063971 | 985,455 | 567,189 | 1256,632 | 711,689 | 1,737 | 1,766 | 1,752 | tai | taiman |
| A_09_P221300 | 358,595 | 179,520 | 373,663 | 187,580 | 1,998 | 1,992 | 1,995 | Task6 | TWIK-related acid-sensitive K[] channel 6 |
| A_09_P186530 | 212,231 | 114,004 | 298,325 | 154,999 | 1,862 | 1,925 | 1,893 | Ten-a | Tenascin accessory |
| A_09_P213450 | 90,584 | 37,426 | 184,370 | 74,076 | 2,420 | 2,489 | 2,455 | Tep2 | Thioester-containing protein 2 |
| A_09_P064071 | 191,372 | 101,451 | 513,681 | 205,531 | 1,886 | 2,499 | 2,193 | Tep2 | Thioester-containing protein 2 |
| A_09_P197870 | 2661,921 | 734,938 | 3227,711 | 1112,289 | 3,622 | 2,902 | 3,262 | Theg | Testicular haploid expressed gene |
| A_09_P126325 | 132,097 | 77,477 | 272,633 | 113,930 | 1,705 | 2,393 | 2,049 | Tim17a1 | Tim17a1 |
| A_09_P165580 | 67,111 | 34,043 | 178,879 | 109,962 | 1,971 | 1,627 | 1,799 | Tis11 | Tis11 zinc finger protein |
| A_09_P021051 | 81,003 | 29,522 | 120,637 | 52,788 | 2,744 | 2,285 | 2,515 | Toll-4 | Toll-4 |
| A_09_P066436 | 274,533 | 166,370 | 587,292 | 237,797 | 1,650 | 2,470 | 2,060 | Tollo | Tollo |
| A_09_P026216 | 36,840 | 6,941 | 28,505 | 4,434 | 5,308 | 6,429 | 5,868 | tpr | tracheal-prostasin |
| A_09_P161285 | 31,440 | 6,186 | 56,265 | 31,488 | 5,082 | 1,787 | 3,435 | TrissinR | Trissin receptor |
| A_09_P181405 | 383,060 | 50,103 | 497,608 | 106,085 | 7,645 | 4,691 | 6,168 | trol | terribly reduced optic lobes |
| A_09_P077116 | 47,153 | 10,071 | 110,041 | 54,396 | 4,682 | 2,023 | 3,353 | Ts | Thymidylate synthase |
| A_09_P190810 | 57,307 | 13,680 | 43,169 | 18,930 | 4,189 | 2,280 | 3,235 | Tsp29Fa | Tetraspanin 29Fa |
| A_09_P020971 | 70,030 | 37,894 | 89,978 | 46,369 | 1,848 | 1,940 | 1,894 | Tsp29Fa | Tetraspanin 29Fa |
| A_09_P024626 | 287,138 | 143,628 | 421,115 | 285,838 | 1,999 | 1,473 | 1,736 | Tsp42En | Tetraspanin 42En |
| A_09_P020521 | 73,775 | 44,520 | 160,520 | 79,484 | 1,657 | 2,020 | 1,838 | TwdlE | TweedleE |
| A_09_P074486 | 1411,614 | 384,316 | 3453,294 | 1377,597 | 3,673 | 2,507 | 3,090 | Ugt37A2 | UDP-glycosyltransferase family 37 member A2 |
| A_09_P167045 | 264,875 | 98,885 | 325,907 | 177,544 | 2,679 | 1,836 | 2,257 | unc79 | uncoordinated 79 |
| A_09_P150245 | 6683,232 | 3130,122 | 8232,296 | 4028,568 | 2,135 | 2,043 | 2,089 | Unr | Upstream of N-ras |
| A_09_P000216 | 413,362 | 265,082 | 1008,048 | 380,026 | 1,559 | 2,653 | 2,106 | upd3 | unpaired 3 |
| A_09_P185995 | 6307,211 | 3433,128 | 8304,772 | 5329,043 | 1,837 | 1,558 | 1,698 | Updo | Updo |
| A_09_P005746 | 11869,607 | 7740,602 | 15496,183 | 9790,209 | 1,533 | 1,583 | 1,558 | Updo | Updo |
| A_09_P037991 | 12142,185 | 3280,673 | 18218,158 | 6988,815 | 3,701 | 2,607 | 3,154 | Usp7 | Ubiquitin-specific protease 7 |
| A_09_P009976 | 98,789 | 55,215 | 218,916 | 138,455 | 1,789 | 1,581 | 1,685 | v | vermilion |
| A_09_P066121 | 53,886 | 10,287 | 129,628 | 32,459 | 5,238 | 3,994 | 4,616 | Vajk1 | - |
| A_09_P057646 | 265,426 | 93,566 | 641,148 | 356,924 | 2,837 | 1,796 | 2,317 | Vajk4 | - |
| A_09_P068876 | 86,266 | 26,254 | 171,967 | 39,887 | 3,286 | 4,311 | 3,799 | Vha100-4 | Vacuolar H[] ATPase 100kD subunit 4 |
| A_09_P219695 | 1666,766 | 528,303 | 5076,220 | 1981,309 | 3,155 | 2,562 | 2,859 | Vha26 | Vacuolar H[]-ATPase 26kD subunit |
| A_09_P176985 | 121,773 | 26,761 | 115,022 | 43,458 | 4,550 | 2,647 | 3,599 | Vha68-3 | Vacuolar H[] ATPase 68kD subunit 3 |
| A_09_P016861 | 120,909 | 7,564 | 114,009 | 5,891 | 15,985 | 19,353 | 17,669 | Victoria | Victoria |
| A_09_P031486 | 1929,395 | 692,617 | 1911,748 | 868,677 | 2,786 | 2,201 | 2,493 | vkg | viking |
| A_09_P177925 | 86,826 | 50,732 | 149,929 | 93,315 | 1,711 | 1,607 | 1,659 | Vps52 | Vacuolar protein sorting 52 |
| A_09_P026321 | 596,840 | 293,942 | 844,357 | 572,489 | 2,030 | 1,475 | 1,753 | Vrp1 | Verprolin 1 |
| A_09_P194840 | 3291,554 | 451,711 | 752,705 | 195,715 | 7,287 | 3,846 | 5,566 | vtd | verthandi |
| A_09_P046191 | 427,466 | 14,510 | 494,206 | 27,598 | 29,460 | 17,907 | 23,684 | w | white |
| A_09_P010031 | 1031,506 | 68,708 | 1464,451 | 88,526 | 15,013 | 16,543 | 15,778 | w | white |
| A_09_P070321 | 671,676 | 281,177 | 864,479 | 465,260 | 2,389 | 1,858 | 2,123 | wake | wide awake |
| A_09_P210050 | 40,384 | 11,382 | 51,415 | 22,645 | 3,548 | 2,270 | 2,909 | wry | weary |
| A_09_P022821 | 262,898 | 108,760 | 655,016 | 84,008 | 2,417 | 7,797 | 5,107 | yellow-b | yellow-b |
| A_09_P010936 | 35,375 | 8,363 | 90,495 | 38,563 | 4,230 | 2,347 | 3,288 | yl | yolkless |
| A_09_P010831 | 378,825 | 245,714 | 710,165 | 380,691 | 1,542 | 1,865 | 1,704 | zfh1 | Zn finger homeodomain 1 |

**Table S4. List of genes down-regulated upon hTau^WT^ expression in *Drosophila* Primary Neurons**

|  | **Experiment N°1** | | **Experiment N°2** | |  | | | | |
| --- | --- | --- | --- | --- | --- | --- | --- | --- | --- |
| **Probe ID** | **Cy5 processed signal** | **Cy3 processed signal** | **Cy5 processed signal** | **Cy3 processed signal** | **FoldChge 1** | **Foldchge 2** | **Mean FoldChge** | **Gene Symbol** | **Gene Name** |
| A_09_P041076 | 53,306 | 103,524 | 82,180 | 227,395 | 0,515 | 0,361 | 0,438 | Abd-B | Abdominal B |
| A_09_P075991 | 114,556 | 250,669 | 230,095 | 514,395 | 0,457 | 0,447 | 0,452 | AdSL | Adenylosuccinate Lyase |
| A_09_P041311 | 569,63 | 885,339 | 687,529 | 1127,778 | 0,643 | 0,610 | 0,627 | Aprt | Adenine phosphoribosyltransferase |
| A_09_P090440 | 5,757 | 185,638 | 7,27 | 374,472 | 0,031 | 0,019 | 0,025 | Argl | Argininosuccinate lyase |
| A_09_P021876 | 519,916 | 780,75 | 706,179 | 1129,422 | 0,666 | 0,625 | 0,646 | Art8 | Arginine methyltransferase 8 |
| A_09_P026031 | 375,262 | 1049,673 | 762,111 | 1324,86 | 0,358 | 0,575 | 0,466 | ASPP | Ankyrin-repeat, SH3-domain, and Proline-rich-region containing Protein |
| A_09_P115485 | 266,364 | 404,278 | 308,643 | 588,133 | 0,659 | 0,525 | 0,592 | asRNA:CR42254 | antisense RNA:CR42254 |
| A_09_P049166 | 3402,04 | 6294,47 | 5840,994 | 10557,866 | 0,540 | 0,553 | 0,547 | Atx2 | Ataxin-2 |
| A_09_P018541 | 240,77 | 439,515 | 428,755 | 1135,102 | 0,548 | 0,378 | 0,463 | Atxn7 | Ataxin 7 |
| A_09_P041386 | 58721,777 | 87347,93 | 91320,62 | 146048,67 | 0,672 | 0,625 | 0,649 | awd | abnormal wing discs |
| A_09_P006286 | 88,327 | 147,597 | 122,986 | 197,358 | 0,598 | 0,623 | 0,611 | BBS4 | Bardet-Biedl syndrome 4 |
| A_09_P036161 | 36,825 | 70,187 | 40,378 | 67,661 | 0,525 | 0,597 | 0,561 | BBS5 | Bardet-Biedl syndrome 5 |
| A_09_P090650 | 57,04 | 521,127 | 226,275 | 1124,973 | 0,109 | 0,201 | 0,155 | beat-Ia | beaten path Ia |
| A_09_P113005 | 60,477 | 169,568 | 82,317 | 170,961 | 0,357 | 0,481 | 0,419 | bma | black match |
| A_09_P003921 | 61,142 | 191,868 | 87,066 | 197,86 | 0,319 | 0,440 | 0,379 | bma | black match |
| A_09_P057811 | 229,965 | 354,682 | 326,29 | 494,666 | 0,648 | 0,660 | 0,654 | Brca2 | BRCA2, DNA repair associated |
| A_09_P041546 | 155,211 | 310,187 | 243,347 | 466,795 | 0,500 | 0,521 | 0,511 | c(3)G | crossover suppressor on 3 of Gowen |
| A_09_P105800 | 120,901 | 257,803 | 188,706 | 327,019 | 0,469 | 0,577 | 0,523 | C15 | C15 |
| A_09_P222550 | 351,533 | 561,944 | 552,77 | 1031,966 | 0,626 | 0,536 | 0,581 | Ca-alpha1T | Ca[2]-channel protein alpha[[1]] subunit T |
| A_09_P061106 | 37,47 | 72,546 | 86,999 | 173,541 | 0,516 | 0,501 | 0,509 | Cad99C | Cadherin 99C |
| A_09_P144915 | 73,746 | 136,191 | 115,864 | 271,48 | 0,541 | 0,427 | 0,484 | Cbl | Cbl proto-oncogene |
| A_09_P145910 | 173,103 | 272,615 | 167,487 | 296,676 | 0,635 | 0,565 | 0,600 | CG10077 | - |
| A_09_P024226 | 647,856 | 989,903 | 669,64 | 1127,059 | 0,654 | 0,594 | 0,624 | CG10395 | - |
| A_09_P115860 | 181,612 | 322,9 | 284,058 | 430,178 | 0,562 | 0,660 | 0,611 | CG10469 | - |
| A_09_P053106 | 256,41 | 418,553 | 468,469 | 672,254 | 0,613 | 0,697 | 0,655 | CG10479 | - |
| A_09_P053101 | 528,343 | 879,481 | 770,598 | 1229,806 | 0,601 | 0,627 | 0,614 | CG10483 | - |
| A_09_P023291 | 114,244 | 179,088 | 205,385 | 316,541 | 0,638 | 0,649 | 0,643 | CG10623 | - |
| A_09_P023726 | 328,674 | 593,974 | 476,703 | 805,861 | 0,553 | 0,592 | 0,572 | CG10949 | - |
| A_09_P020151 | 13,513 | 32,800 | 21,564 | 58,238 | 0,412 | 0,370 | 0,391 | CG11236 | - |
| A_09_P020061 | 1375,872 | 2932,45 | 2451,312 | 3776,014 | 0,469 | 0,649 | 0,559 | CG11319 | - |
| A_09_P189330 | 1636,277 | 3447,934 | 3090,53 | 4995,899 | 0,475 | 0,619 | 0,547 | CG11319 | - |
| A_09_P219130 | 310,45 | 469,208 | 414,673 | 622,643 | 0,662 | 0,666 | 0,664 | CG11360 | - |
| A_09_P178530 | 68,006 | 125,082 | 92,481 | 145,872 | 0,544 | 0,634 | 0,589 | CG1146 | - |
| A_09_P026291 | 145,863 | 269,573 | 166,600 | 414,297 | 0,541 | 0,402 | 0,472 | CG11474 | - |
| A_09_P006106 | 322,944 | 498,428 | 566,522 | 1017,435 | 0,648 | 0,557 | 0,602 | CG11825 | - |
| A_09_P107930 | 288,594 | 450,312 | 463,623 | 879,902 | 0,641 | 0,527 | 0,584 | CG11825 | - |
| A_09_P028456 | 8,73 | 46,827 | 23,22 | 85,561 | 0,186 | 0,271 | 0,229 | CG12011 | - |
| A_09_P137325 | 755,346 | 1150,649 | 866,083 | 1689,861 | 0,656 | 0,513 | 0,584 | CG12096 | - |
| A_09_P065236 | 110,037 | 189,403 | 156,487 | 290,479 | 0,581 | 0,539 | 0,560 | CG12182 | - |
| A_09_P078171 | 372,045 | 560,575 | 560,362 | 988,978 | 0,664 | 0,567 | 0,615 | CG12253 | - |
| A_09_P052796 | 213,557 | 335,167 | 362,991 | 762,822 | 0,637 | 0,476 | 0,557 | CG12493 | - |
| A_09_P054811 | 28,212 | 118,328 | 28,257 | 165,698 | 0,238 | 0,171 | 0,204 | CG12522 | - |
| A_09_P110950 | 52,613 | 197,663 | 89,982 | 141,391 | 0,266 | 0,636 | 0,451 | CG12655 | - |
| A_09_P040706 | 322,432 | 1573,484 | 468,229 | 1344,226 | 0,205 | 0,348 | 0,277 | CG12655 | - |
| A_09_P057086 | 134,711 | 258,998 | 150,661 | 248,565 | 0,520 | 0,606 | 0,563 | CG12699 | - |
| A_09_P056396 | 77,239 | 123,975 | 99,920 | 160,743 | 0,623 | 0,622 | 0,622 | CG12713 | - |
| A_09_P029206 | 99,396 | 171,348 | 172,862 | 294,007 | 0,580 | 0,588 | 0,584 | CG12766 | - |
| A_09_P129880 | 100,632 | 174,650 | 157,205 | 236,499 | 0,576 | 0,665 | 0,620 | CG12811 | - |
| A_09_P029311 | 82,153 | 119,906 | 149,858 | 316,676 | 0,685 | 0,473 | 0,579 | CG1299 | - |
| A_09_P006466 | 131,716 | 380,731 | 218,399 | 514,121 | 0,346 | 0,425 | 0,385 | CG13203 | - |
| A_09_P045431 | 91,927 | 391,705 | 207,338 | 490,810 | 0,235 | 0,422 | 0,329 | CG13203 | - |
| A_09_P006461 | 1589,234 | 2925,153 | 2545,663 | 3860,397 | 0,543 | 0,659 | 0,601 | CG13204 | - |
| A_09_P063526 | 21,544 | 53,094 | 30,896 | 60,708 | 0,406 | 0,509 | 0,457 | CG13230 | - |
| A_09_P057006 | 75,625 | 310,837 | 70,856 | 406,734 | 0,243 | 0,174 | 0,209 | CG13245 | - |
| A_09_P144065 | 26,753 | 76,321 | 83,8 | 226,05 | 0,351 | 0,371 | 0,361 | CG13332 | - |
| A_09_P025781 | 31,418 | 76,623 | 58,642 | 102,789 | 0,410 | 0,571 | 0,490 | CG13436 | - |
| A_09_P026676 | 1303,664 | 2195,193 | 1720,638 | 3501,988 | 0,594 | 0,491 | 0,543 | CG13532 | - |
| A_09_P071136 | 44,843 | 97,019 | 66,045 | 122,668 | 0,462 | 0,538 | 0,500 | CG13617 | - |
| A_09_P071221 | 4813,371 | 8214,135 | 6620,613 | 10863,488 | 0,586 | 0,609 | 0,598 | CG13630 | - |
| A_09_P071586 | 33,378 | 61,24 | 36,86 | 57,052 | 0,545 | 0,646 | 0,596 | CG13659 | - |
| A_09_P070191 | 374,321 | 578,785 | 501,103 | 858,661 | 0,647 | 0,584 | 0,615 | CG13850 | - |
| A_09_P129320 | 68,361 | 109,634 | 102,507 | 164,025 | 0,624 | 0,625 | 0,624 | CG13898 | - |
| A_09_P028156 | 34,647 | 94,153 | 51,222 | 93,736 | 0,368 | 0,546 | 0,457 | CG13905 | - |
| A_09_P035791 | 18,930 | 42,528 | 18,309 | 55,985 | 0,445 | 0,327 | 0,386 | CG14457 | - |
| A_09_P102080 | 43,943 | 99,389 | 58,744 | 112,653 | 0,442 | 0,521 | 0,482 | CG14566 | - |
| A_09_P035611 | 115,861 | 194,808 | 166,082 | 368,922 | 0,595 | 0,450 | 0,522 | CG14567 | - |
| A_09_P035936 | 92,287 | 411,621 | 155,506 | 765,861 | 0,224 | 0,203 | 0,214 | CG14636 | - |
| A_09_P139055 | 136,141 | 298,305 | 68,648 | 269,28 | 0,456 | 0,255 | 0,356 | CG14757 | - |
| A_09_P022061 | 127,274 | 299,996 | 75,413 | 443,266 | 0,424 | 0,170 | 0,297 | CG14932 | - |
| A_09_P046791 | 77,753 | 200,284 | 37,108 | 236,834 | 0,388 | 0,157 | 0,272 | CG14932 | - |
| A_09_P028966 | 79,362 | 135,663 | 59,572 | 141,241 | 0,585 | 0,422 | 0,503 | CG14963 | - |
| A_09_P217210 | 71,374 | 175,531 | 68,235 | 172,039 | 0,407 | 0,397 | 0,402 | CG14963 | - |
| A_09_P040081 | 32,315 | 71,847 | 16,856 | 63,755 | 0,450 | 0,264 | 0,357 | CG15056 | - |
| A_09_P009266 | 112,853 | 246,411 | 351,618 | 721,959 | 0,458 | 0,487 | 0,473 | CG15093 | - |
| A_09_P024536 | 1421,985 | 2461,613 | 1104,509 | 3538,145 | 0,578 | 0,312 | 0,445 | CG15236 | - |
| A_09_P066946 | 37,764 | 70,728 | 41,303 | 75,240 | 0,534 | 0,549 | 0,541 | CG15375 | - |
| A_09_P018456 | 126,531 | 194,588 | 186,491 | 269,862 | 0,650 | 0,691 | 0,671 | CG15385 | - |
| A_09_P067931 | 283,121 | 600,574 | 518,521 | 927,187 | 0,471 | 0,559 | 0,515 | CG15478 | - |
| A_09_P061521 | 50,408 | 92,96 | 49,747 | 155,337 | 0,542 | 0,320 | 0,431 | CG15547 | - |
| A_09_P116195 | 798,29 | 1830,272 | 1015,849 | 2893,701 | 0,436 | 0,351 | 0,394 | CG15547 | - |
| A_09_P063456 | 83,878 | 177,610 | 100,963 | 236,203 | 0,472 | 0,427 | 0,450 | CG16926 | - |
| A_09_P022461 | 1566,85 | 2431,719 | 1989,191 | 4111,789 | 0,644 | 0,484 | 0,564 | CG16974 | - |
| A_09_P163120 | 150,047 | 251,594 | 104,37 | 248,122 | 0,596 | 0,421 | 0,509 | CG16986 | - |
| A_09_P070406 | 187,735 | 304,956 | 237,067 | 479,102 | 0,616 | 0,495 | 0,555 | CG17141 | - |
| A_09_P063171 | 144,332 | 362,434 | 315,293 | 546,875 | 0,398 | 0,577 | 0,487 | CG17193 | - |
| A_09_P219520 | 75,614 | 188,205 | 125,201 | 367,834 | 0,402 | 0,340 | 0,371 | CG17233 | - |
| A_09_P202905 | 307,689 | 582,565 | 378,663 | 745,816 | 0,528 | 0,508 | 0,518 | CG17278 | - |
| A_09_P057171 | 915,357 | 1832,572 | 1513,326 | 3042,861 | 0,499 | 0,497 | 0,498 | CG17278 | - |
| A_09_P006246 | 15,992 | 42,876 | 28,437 | 65,931 | 0,373 | 0,431 | 0,402 | CG18003 | - |
| A_09_P033996 | 183,131 | 317,012 | 230,875 | 355,944 | 0,578 | 0,649 | 0,613 | CG18265 | - |
| A_09_P037096 | 54,113 | 109,368 | 87,617 | 136,397 | 0,495 | 0,642 | 0,569 | CG18268 | - |
| A_09_P007561 | 519,919 | 783,392 | 777,264 | 1305,481 | 0,664 | 0,595 | 0,630 | CG18324 | - |
| A_09_P120170 | 20,178 | 79,427 | 16,205 | 111,427 | 0,254 | 0,145 | 0,200 | CG18754 | - |
| A_09_P060761 | 128,827 | 297,148 | 315,041 | 580,889 | 0,434 | 0,542 | 0,488 | CG1951 | - |
| A_09_P169460 | 267,411 | 459,829 | 440,602 | 666,699 | 0,582 | 0,661 | 0,621 | CG2225 | - |
| A_09_P057326 | 322,059 | 491,606 | 411,23 | 693,429 | 0,655 | 0,593 | 0,624 | CG30010 | - |
| A_09_P026771 | 134,434 | 217,573 | 182,076 | 417,998 | 0,618 | 0,436 | 0,527 | CG3085 | - |
| A_09_P067346 | 227,553 | 468,779 | 765,688 | 1387,411 | 0,485 | 0,552 | 0,519 | CG3097 | - |
| A_09_P058801 | 57,719 | 240,745 | 127,189 | 249,395 | 0,240 | 0,510 | 0,375 | CG31038 | - |
| A_09_P218080 | 33,870 | 88,662 | 50,724 | 83,727 | 0,382 | 0,606 | 0,494 | CG31141 | - |
| A_09_P059131 | 16,300 | 67,122 | 27,295 | 48,703 | 0,243 | 0,560 | 0,402 | CG31141 | - |
| A_09_P059381 | 181,805 | 270,341 | 180,825 | 354,13 | 0,673 | 0,511 | 0,592 | CG31229 | - |
| A_09_P059651 | 80,579 | 125,056 | 58,217 | 99,498 | 0,644 | 0,585 | 0,615 | CG31345 | - |
| A_09_P059806 | 236,232 | 371,237 | 250,521 | 382,341 | 0,636 | 0,655 | 0,646 | CG31415 | - |
| A_09_P059836 | 79,789 | 136,989 | 92,490 | 146,486 | 0,582 | 0,631 | 0,607 | CG31431 | - |
| A_09_P060361 | 166,894 | 249,632 | 260,932 | 403,683 | 0,669 | 0,646 | 0,657 | CG31664 | - |
| A_09_P179335 | 137,551 | 314,367 | 161,621 | 518,054 | 0,438 | 0,312 | 0,375 | CG31673 | - |
| A_09_P060381 | 396,202 | 1297,480 | 426,227 | 1630,091 | 0,305 | 0,261 | 0,283 | CG31673 | - |
| A_09_P060401 | 6,278 | 34,406 | 10,150 | 41,102 | 0,182 | 0,247 | 0,215 | CG31677 | - |
| A_09_P090045 | 122,455 | 233,570 | 318,336 | 487,962 | 0,524 | 0,652 | 0,588 | CG31759 | - |
| A_09_P160565 | 82,823 | 187,653 | 128,849 | 249,586 | 0,441 | 0,516 | 0,479 | CG31808 | - |
| A_09_P130455 | 248,351 | 404,566 | 435,935 | 742,613 | 0,614 | 0,587 | 0,600 | CG31855 | - |
| A_09_P014066 | 31,069 | 140,623 | 112,085 | 209,210 | 0,221 | 0,536 | 0,378 | CG31904 | - |
| A_09_P181385 | 183,456 | 326,611 | 219,569 | 647,230 | 0,562 | 0,339 | 0,450 | CG31957 | - |
| A_09_P014811 | 47,383 | 96,543 | 52,598 | 170,468 | 0,491 | 0,309 | 0,400 | CG32182 | - |
| A_09_P014871 | 413,135 | 664,376 | 435,630 | 675,573 | 0,622 | 0,645 | 0,633 | CG32195 | - |
| A_09_P037156 | 1172,405 | 2453,848 | 1491,82 | 3264,421 | 0,478 | 0,457 | 0,467 | CG3223 | - |
| A_09_P015131 | 216,447 | 323,268 | 310,537 | 540,508 | 0,670 | 0,575 | 0,622 | CG32267 | - |
| A_09_P015351 | 598,624 | 1238,394 | 460,305 | 1459,939 | 0,483 | 0,315 | 0,399 | CG32368 | - |
| A_09_P177585 | 108,121 | 280,411 | 165,207 | 355,85 | 0,386 | 0,464 | 0,425 | CG32369 | - |
| A_09_P015776 | 90,629 | 177,856 | 181,743 | 305,227 | 0,510 | 0,595 | 0,552 | CG32537 | - |
| A_09_P198000 | 149,204 | 551,16 | 166,718 | 672,939 | 0,271 | 0,248 | 0,259 | CG3262 | - |
| A_09_P115755 | 72,987 | 218,130 | 189,400 | 550,358 | 0,335 | 0,344 | 0,339 | CG32820 | - |
| A_09_P016731 | 50,956 | 219,108 | 99,598 | 328,844 | 0,233 | 0,303 | 0,268 | CG33054 | - |
| A_09_P016886 | 22,416 | 38,175 | 15,296 | 27,854 | 0,587 | 0,549 | 0,568 | CG33125 | - |
| A_09_P017646 | 350,827 | 601,248 | 108,798 | 568,031 | 0,583 | 0,192 | 0,388 | CG33468 | - |
| A_09_P000791 | 28,886 | 59,804 | 26,659 | 102,292 | 0,483 | 0,261 | 0,372 | CG33914 | - |
| A_09_P001076 | 89,658 | 151,414 | 90,601 | 150,45 | 0,592 | 0,602 | 0,597 | CG34027 | - |
| A_09_P048836 | 107,958 | 179,175 | 164,281 | 299,495 | 0,603 | 0,549 | 0,576 | CG34112 | - |
| A_09_P162830 | 1014,28 | 1575,42 | 1155,182 | 1998,973 | 0,644 | 0,578 | 0,611 | CG34174 | - |
| A_09_P003151 | 164,377 | 318,504 | 185,267 | 531,744 | 0,516 | 0,348 | 0,432 | CG34195 | - |
| A_09_P003381 | 134,348 | 528,525 | 111,436 | 748,108 | 0,254 | 0,149 | 0,202 | CG34242 | - |
| A_09_P100390 | 31,881 | 299,925 | 46,399 | 454,728 | 0,106 | 0,102 | 0,104 | CG34242 | - |
| A_09_P003791 | 85,801 | 209,793 | 104,390 | 323,296 | 0,409 | 0,323 | 0,366 | CG34332 | - |
| A_09_P186735 | 1133,913 | 2007,302 | 1951,824 | 3526,939 | 0,565 | 0,553 | 0,559 | CG3634 | - |
| A_09_P197190 | 176,669 | 331,276 | 261,237 | 489,158 | 0,533 | 0,534 | 0,534 | CG3792 | - |
| A_09_P062986 | 2665,665 | 4259,015 | 3778,741 | 5672,318 | 0,626 | 0,666 | 0,646 | CG3939 | - |
| A_09_P203370 | 366,917 | 603,298 | 563,148 | 858,763 | 0,608 | 0,656 | 0,632 | CG3939 | - |
| A_09_P001501 | 110,252 | 193,284 | 166,979 | 257,665 | 0,570 | 0,648 | 0,609 | CG40189 | - |
| A_09_P001631 | 717,389 | 1187,488 | 1135,432 | 2101,914 | 0,604 | 0,540 | 0,572 | CG40275 | - |
| A_09_P001816 | 105,795 | 267,458 | 141,539 | 349,520 | 0,396 | 0,405 | 0,400 | CG40470 | - |
| A_09_P059221 | 181,312 | 560,472 | 427,521 | 915,212 | 0,323 | 0,467 | 0,395 | CG42335 | - |
| A_09_P210260 | 7,15 | 59,133 | 20,087 | 58,325 | 0,121 | 0,344 | 0,233 | CG42337 | - |
| A_09_P133980 | 24,554 | 56,739 | 26,159 | 53,91 | 0,433 | 0,485 | 0,459 | CG42445 | - |
| A_09_P091085 | 121,234 | 264,335 | 138,793 | 394,284 | 0,459 | 0,352 | 0,405 | CG42494 | - |
| A_09_P028531 | 49,291 | 392,53 | 76,356 | 461,618 | 0,126 | 0,165 | 0,145 | CG42676 | - |
| A_09_P049746 | 1298,520 | 2064,893 | 912,527 | 1653,322 | 0,629 | 0,552 | 0,590 | CG42808 | - |
| A_09_P196230 | 235,103 | 367,039 | 338,451 | 608,572 | 0,641 | 0,556 | 0,598 | CG43085 | - |
| A_09_P069471 | 384,143 | 1206,982 | 587,391 | 2061,513 | 0,318 | 0,285 | 0,302 | CG4390 | - |
| A_09_P148265 | 179,599 | 361,745 | 299,632 | 724,730 | 0,496 | 0,413 | 0,455 | CG4415 | - |
| A_09_P018116 | 60,075 | 141,433 | 117,030 | 306,708 | 0,425 | 0,382 | 0,403 | CG4415 | - |
| A_09_P037636 | 9,499 | 33,943 | 13,606 | 34,732 | 0,280 | 0,392 | 0,336 | CG44422 | - |
| A_09_P218055 | 183,322 | 886,576 | 302,883 | 1029,530 | 0,207 | 0,294 | 0,250 | CG4467 | - |
| A_09_P202110 | 128,581 | 265,023 | 108,297 | 243,287 | 0,485 | 0,445 | 0,465 | CG45049 | - |
| A_09_P208670 | 28,434 | 483,265 | 62,291 | 434,200 | 0,059 | 0,143 | 0,101 | CG45487 | - |
| A_09_P171345 | 22,760 | 354,523 | 44,025 | 380,459 | 0,064 | 0,116 | 0,090 | CG45487 | - |
| A_09_P169816 | 4,187 | 381,389 | 21,142 | 345,997 | 0,011 | 0,061 | 0,036 | CG45487 | - |
| A_09_P074191 | 31,86 | 72,391 | 41,969 | 84,278 | 0,440 | 0,498 | 0,469 | CG4830 | - |
| A_09_P065951 | 267,685 | 428,421 | 239,562 | 417,151 | 0,625 | 0,574 | 0,600 | CG4892 | - |
| A_09_P054231 | 128,694 | 280,112 | 167,389 | 282,284 | 0,459 | 0,593 | 0,526 | CG4942 | - |
| A_09_P021426 | 284,788 | 437,499 | 404,305 | 753,338 | 0,651 | 0,537 | 0,594 | CG4953 | - |
| A_09_P021486 | 125,146 | 195,816 | 192,578 | 305,285 | 0,639 | 0,631 | 0,635 | CG4972 | - |
| A_09_P021556 | 145,703 | 274,871 | 271,218 | 445,313 | 0,530 | 0,609 | 0,570 | CG5096 | - |
| A_09_P075646 | 13,206 | 40,876 | 17,673 | 49,897 | 0,323 | 0,354 | 0,339 | CG5478 | - |
| A_09_P027086 | 1945,499 | 3346,373 | 2401,392 | 6498,666 | 0,581 | 0,370 | 0,475 | CG5532 | - |
| A_09_P069136 | 1194,359 | 1872,423 | 1830,837 | 2987,426 | 0,638 | 0,613 | 0,625 | CG5555 | - |
| A_09_P035036 | 1677,292 | 2912,591 | 2012,548 | 3351,573 | 0,576 | 0,600 | 0,588 | CG5618 | - |
| A_09_P078711 | 90,138 | 181,754 | 129,921 | 282,414 | 0,496 | 0,460 | 0,478 | CG5704 | - |
| A_09_P078761 | 24,800 | 53,315 | 40,504 | 123,993 | 0,465 | 0,327 | 0,396 | CG5707 | - |
| A_09_P224625 | 62,179 | 109,826 | 92,947 | 159,575 | 0,566 | 0,582 | 0,574 | CG5746 | - |
| A_09_P069836 | 123,671 | 201,794 | 153,557 | 257,409 | 0,613 | 0,597 | 0,605 | CG5810 | - |
| A_09_P124490 | 69,951 | 164,87 | 105,893 | 185,497 | 0,424 | 0,571 | 0,498 | CG5810 | - |
| A_09_P110270 | 254,097 | 384,522 | 389,3 | 792,284 | 0,661 | 0,491 | 0,576 | CG5969 | - |
| A_09_P070901 | 54,88 | 238,414 | 59,806 | 314,706 | 0,230 | 0,190 | 0,210 | CG6000 | - |
| A_09_P118320 | 396,133 | 667,206 | 568,607 | 987,722 | 0,594 | 0,576 | 0,585 | CG6071 | - |
| A_09_P055021 | 357,512 | 737,429 | 548,787 | 1092,263 | 0,485 | 0,502 | 0,494 | CG6071 | - |
| A_09_P054846 | 17,688 | 39,464 | 23,586 | 62,413 | 0,448 | 0,378 | 0,413 | CG6216 | - |
| A_09_P007386 | 38,931 | 75,663 | 30,836 | 52,138 | 0,515 | 0,591 | 0,553 | CG6220 | - |
| A_09_P008771 | 36,277 | 76,269 | 45,333 | 88,798 | 0,476 | 0,511 | 0,493 | CG6484 | - |
| A_09_P112980 | 22,972 | 55,959 | 14,208 | 54,947 | 0,411 | 0,259 | 0,335 | CG6602 | - |
| A_09_P075321 | 416,234 | 793,237 | 601,659 | 1229,549 | 0,525 | 0,489 | 0,507 | CG6654 | - |
| A_09_P024116 | 5,441 | 51,073 | 4,793 | 75,229 | 0,107 | 0,064 | 0,085 | CG6675 | - |
| A_09_P217155 | 4,963 | 108,102 | 11,051 | 125,988 | 0,046 | 0,088 | 0,067 | CG6675 | - |
| A_09_P070221 | 200,819 | 387,183 | 325,800 | 697,881 | 0,519 | 0,467 | 0,493 | CG7054 | - |
| A_09_P124335 | 669,891 | 1151,131 | 1076,343 | 1882,791 | 0,582 | 0,572 | 0,577 | CG7083 | - |
| A_09_P055141 | 14,924 | 39,186 | 17,89 | 66,083 | 0,381 | 0,271 | 0,326 | CG7264 | - |
| A_09_P040276 | 250,533 | 515,021 | 396,801 | 1101,416 | 0,486 | 0,360 | 0,423 | CG7322 | - |
| A_09_P053811 | 14,523 | 28,983 | 6,035 | 28,955 | 0,501 | 0,208 | 0,355 | CG7366 | - |
| A_09_P132610 | 209,094 | 475,735 | 331,543 | 790,882 | 0,440 | 0,419 | 0,429 | CG7376 | - |
| A_09_P074536 | 64,281 | 178,057 | 122,78 | 253,16 | 0,361 | 0,485 | 0,423 | CG7381 | - |
| A_09_P009441 | 439,259 | 785,597 | 583,317 | 1254,652 | 0,559 | 0,465 | 0,512 | CG7461 | - |
| A_09_P006441 | 558,364 | 813,787 | 722,958 | 1130,897 | 0,686 | 0,639 | 0,663 | CG7741 | - |
| A_09_P061261 | 820,504 | 1393,744 | 1257,592 | 2498,622 | 0,589 | 0,503 | 0,546 | CG7950 | - |
| A_09_P075096 | 104,544 | 169,525 | 107,727 | 223,21 | 0,617 | 0,483 | 0,550 | CG8066 | - |
| A_09_P007891 | 56,928 | 105,629 | 83,054 | 151,394 | 0,539 | 0,549 | 0,544 | CG8089 | - |
| A_09_P008036 | 332,753 | 688,622 | 288,833 | 1031,684 | 0,483 | 0,280 | 0,382 | CG8204 | - |
| A_09_P073131 | 411,876 | 613,356 | 743,73 | 1152,812 | 0,672 | 0,645 | 0,658 | CG8319 | - |
| A_09_P217650 | 256,971 | 433,24 | 458,771 | 698,316 | 0,593 | 0,657 | 0,625 | CG8319 | - |
| A_09_P006681 | 1638,296 | 2987,884 | 2383,945 | 4431,316 | 0,548 | 0,538 | 0,543 | CG8407 | - |
| A_09_P028186 | 141,565 | 206,256 | 158,945 | 310,279 | 0,686 | 0,512 | 0,599 | CG9119 | - |
| A_09_P028216 | 26,827 | 46,292 | 51,358 | 95,597 | 0,580 | 0,537 | 0,558 | CG9129 | - |
| A_09_P180055 | 1651,525 | 2985,000 | 2665,304 | 5781,720 | 0,553 | 0,461 | 0,507 | CG9331 | - |
| A_09_P023851 | 1417,684 | 2648,161 | 2330,847 | 5106,024 | 0,535 | 0,456 | 0,496 | CG9331 | - |
| A_09_P163585 | 72,272 | 138,264 | 90,032 | 275,403 | 0,523 | 0,327 | 0,425 | CG9527 | - |
| A_09_P068611 | 100,665 | 160,615 | 122,088 | 223,278 | 0,627 | 0,547 | 0,587 | CG9689 | - |
| A_09_P026746 | 36,458 | 86,079 | 26,824 | 84,408 | 0,424 | 0,318 | 0,371 | CG9897 | - |
| A_09_P105590 | 435,364 | 721,649 | 667,225 | 1018,256 | 0,603 | 0,655 | 0,629 | Con | Connectin |
| A_09_P203305 | 116,186 | 224,809 | 172,689 | 327,804 | 0,517 | 0,527 | 0,522 | Con | Connectin |
| A_09_P068871 | 30,515 | 68,213 | 22,982 | 134,078 | 0,447 | 0,171 | 0,309 | cona | corona |
| A_09_P012206 | 2382,596 | 4364,866 | 4326,676 | 6744,558 | 0,546 | 0,642 | 0,594 | corto | corto |
| A_09_P070546 | 405,846 | 1225,509 | 865,356 | 1785,618 | 0,331 | 0,485 | 0,408 | Cow | Carrier of Wingless |
| A_09_P053411 | 6,264 | 8,393 | 4,975 | 6,049 | 0,746 | 0,823 | 0,784 | Cpr65Ea | Cuticular protein 65Ea |
| A_09_P053411 | 6,264 | 8,393 | 4,975 | 6,049 | 0,746 | 0,823 | 0,784 | Cpr65Ea | Cuticular protein 65Ea |
| A_09_P091155 | 4,831 | 30,596 | 4,207 | 40,658 | 0,158 | 0,103 | 0,131 | cry | cryptochrome |
| A_09_P148385 | 1328,94 | 2224,681 | 1267,343 | 3854,726 | 0,597 | 0,329 | 0,463 | Cul1 | Cullin 1 |
| A_09_P006711 | 146,95 | 268,859 | 205,124 | 545,903 | 0,547 | 0,376 | 0,461 | Cyp6g2 | Cyp6g2 |
| A_09_P108985 | 186,935 | 579,839 | 315,894 | 980,358 | 0,322 | 0,322 | 0,322 | Cyp6g2 | Cyp6g2 |
| A_09_P076456 | 83,299 | 134,444 | 165,418 | 255,443 | 0,620 | 0,648 | 0,634 | D2hgdh | D-2-hydroxyglutaric acid dehydrogenase |
| A_09_P147775 | 1523,43 | 2819,955 | 1184,177 | 3345,236 | 0,540 | 0,354 | 0,447 | DCTN5-p25 | Dynactin 5, p25 subunit |
| A_09_P056046 | 24,519 | 51,387 | 21,819 | 45,489 | 0,477 | 0,480 | 0,478 | DCX-EMAP | Doublecortin-domain-containing echinoderm-microtubule-associated protein |
| A_09_P012861 | 1864,057 | 3013,488 | 2130,33 | 3708,74 | 0,619 | 0,574 | 0,596 | dia | diaphanous |
| A_09_P041981 | 574,98 | 937,47 | 783,766 | 1495,861 | 0,613 | 0,524 | 0,569 | Dip-C | Dipeptidase C |
| A_09_P171695 | 53,362 | 225,753 | 78,389 | 327,369 | 0,236 | 0,239 | 0,238 | DJ-1alpha | DJ-1alpha |
| A_09_P063006 | 368,417 | 522,762 | 463,443 | 903,564 | 0,705 | 0,513 | 0,609 | Dlip2 | Dorsal interacting protein 2 |
| A_09_P131730 | 5,826 | 53,41 | 6,365 | 22,086 | 0,109 | 0,288 | 0,199 | Drep4 | DNA fragmentation factor-related protein 4 |
| A_09_P046486 | 211,414 | 322,901 | 290,714 | 497,093 | 0,655 | 0,585 | 0,620 | Drsl5 | Drosomycin-like 5 |
| A_09_P029071 | 495,619 | 858,053 | 688,228 | 1072,512 | 0,578 | 0,642 | 0,610 | Drsl5 | Drosomycin-like 5 |
| A_09_P026081 | 95,856 | 158,504 | 115,58 | 241,622 | 0,605 | 0,478 | 0,542 | eEFSec | eukaryotic translation elongation factor, selenocysteine-specific |
| A_09_P027021 | 34,152 | 119,306 | 61,245 | 220,089 | 0,286 | 0,278 | 0,282 | Eglp4 | Entomoglyceroporin 4 |
| A_09_P060756 | 88,730 | 138,211 | 88,783 | 156,654 | 0,642 | 0,567 | 0,604 | eIF4E6 | eukaryotic translation initiation factor 4E6 |
| A_09_P075636 | 87,838 | 228,08 | 280,385 | 656 | 0,385 | 0,427 | 0,406 | EndoU | Endoribonuclease U-specific |
| A_09_P042401 | 121,047 | 210,153 | 129,778 | 320,072 | 0,576 | 0,405 | 0,491 | FMRFa | FMRFamide |
| A_09_P016631 | 577,201 | 1038,287 | 647,568 | 1518,605 | 0,556 | 0,426 | 0,491 | FoxP | Forkhead box P |
| A_09_P077366 | 2316,806 | 4265,075 | 3311,123 | 6940,951 | 0,543 | 0,477 | 0,510 | Fpps | Farnesyl pyrophosphate synthase |
| A_09_P010966 | 8,067 | 34,189 | 18,997 | 63,683 | 0,236 | 0,298 | 0,267 | fru | fruitless |
| A_09_P052866 | 647,893 | 1007,487 | 892,705 | 1472,518 | 0,643 | 0,606 | 0,625 | Gdap1 | Gdap1 |
| A_09_P079236 | 7,789 | 109,584 | 15,817 | 137,678 | 0,071 | 0,115 | 0,093 | Gfat1 | Glutamine:fructose-6-phosphate aminotransferase 1 |
| A_09_P002101 | 454,704 | 672,886 | 650,846 | 1279,695 | 0,676 | 0,509 | 0,592 | GlcAT-I | Glucuronyltransferase I |
| A_09_P170170 | 53,474 | 116,542 | 89,032 | 137,741 | 0,459 | 0,646 | 0,553 | Gprk1 | G protein-coupled receptor kinase 1 |
| A_09_P064196 | 54,078 | 87,369 | 65,965 | 108,417 | 0,619 | 0,608 | 0,614 | Gr47b | Gustatory receptor 47b |
| A_09_P005601 | 179,7 | 339,886 | 321,762 | 617,053 | 0,529 | 0,521 | 0,525 | GstE13 | Glutathione S transferase E13 |
| A_09_P171435 | 76,294 | 172,912 | 87,926 | 164,362 | 0,441 | 0,535 | 0,488 | gw | gawky |
| A_09_P061861 | 118,707 | 188,545 | 131,494 | 198,705 | 0,630 | 0,662 | 0,646 | Hcf | Host cell factor |
| A_09_P059066 | 218,754 | 336,824 | 273,307 | 518,999 | 0,649 | 0,527 | 0,588 | HDAC11 | Histone deacetylase 11 |
| A_09_P069736 | 191,405 | 471,731 | 346,355 | 817,312 | 0,406 | 0,424 | 0,415 | hdly | hadley |
| A_09_P123110 | 538,057 | 1504,641 | 1117,870 | 2674,734 | 0,358 | 0,418 | 0,388 | hdly | hadley |
| A_09_P005821 | 102,374 | 197,855 | 127,392 | 375,902 | 0,517 | 0,339 | 0,428 | hebe | hebe |
| A_09_P019231 | 116,975 | 226,139 | 97,013 | 367,562 | 0,517 | 0,264 | 0,391 | HP6 | Heterochromatin protein 6 |
| A_09_P007976 | 945,875 | 1452,567 | 1184,443 | 2462,877 | 0,651 | 0,481 | 0,566 | Hr51 | Hormone receptor 51 |
| A_09_P029116 | 673,448 | 1098,538 | 1006,680 | 1683,205 | 0,613 | 0,598 | 0,606 | Ids | Iduronate 2-sulfatase |
| A_09_P045526 | 316,160 | 517,069 | 395,381 | 668,811 | 0,611 | 0,591 | 0,601 | Ids | Iduronate 2-sulfatase |
| A_09_P042901 | 98,234 | 159,006 | 113,058 | 168,603 | 0,618 | 0,671 | 0,644 | inaD | inactivation no afterpotential D |
| A_09_P033186 | 51,024 | 178,387 | 126,446 | 270,571 | 0,286 | 0,467 | 0,377 | inaF-D | inaF-D |
| A_09_P131130 | 91,199 | 305,705 | 193,010 | 427,315 | 0,298 | 0,452 | 0,375 | inaF-D | inaF-D |
| A_09_P030011 | 495,939 | 1105,194 | 926,572 | 1407,986 | 0,449 | 0,658 | 0,553 | InR | Insulin-like receptor |
| A_09_P075981 | 146,121 | 252,592 | 292,533 | 612,358 | 0,578 | 0,478 | 0,528 | Irc | Immune-regulated catalase |
| A_09_P050301 | 926,963 | 2076,281 | 1225,306 | 2281,459 | 0,446 | 0,537 | 0,492 | Jarid2 | Jumonji, AT rich interactive domain 2 |
| A_09_P007766 | 273,329 | 495,102 | 316,144 | 603,567 | 0,552 | 0,524 | 0,538 | jef | jet fuel |
| A_09_P019381 | 128,602 | 351,627 | 126,026 | 265,691 | 0,366 | 0,474 | 0,420 | Jon25Biii | Jonah 25Biii |
| A_09_P053936 | 228,225 | 556,555 | 206,613 | 464,053 | 0,410 | 0,445 | 0,428 | Jon66Ci | Jonah 66Ci |
| A_09_P053941 | 165,137 | 369,627 | 128,504 | 219,215 | 0,447 | 0,586 | 0,516 | Jon66Cii | Jonah 66Cii |
| A_09_P046576 | 34,798 | 95,500 | 46,874 | 98,288 | 0,364 | 0,477 | 0,421 | Jon66Cii | Jonah 66Cii |
| A_09_P044611 | 29,705 | 65,6 | 46,859 | 83,94 | 0,453 | 0,558 | 0,506 | Jon99Ci | Jonah 99Ci |
| A_09_P061396 | 73,851 | 156,316 | 83,548 | 148,8 | 0,472 | 0,561 | 0,517 | Jon99Fii | Jonah 99Fii |
| A_09_P147255 | 56,786 | 100,563 | 72,112 | 138,862 | 0,565 | 0,519 | 0,542 | jtb | jetboil |
| A_09_P069716 | 4494,401 | 6983,781 | 7340,154 | 11139,373 | 0,644 | 0,659 | 0,651 | KaiR1D | Kainate-type ionotropic glutamate receptor subunit 1D |
| A_09_P222300 | 653,947 | 1604,622 | 1044,629 | 2465,933 | 0,408 | 0,424 | 0,416 | KaiR1D | Kainate-type ionotropic glutamate receptor subunit 1D |
| A_09_P062411 | 213,989 | 560,275 | 594,237 | 1169,806 | 0,382 | 0,508 | 0,445 | kat80 | katanin 80 |
| A_09_P066186 | 129,054 | 195,365 | 247,218 | 381,907 | 0,661 | 0,647 | 0,654 | Kebab | Kinetochore and EB1 associated basic protein |
| A_09_P022431 | 31,072 | 86,866 | 90,865 | 191,669 | 0,358 | 0,474 | 0,416 | kmg | kumgang |
| A_09_P079151 | 84,725 | 133,145 | 86,076 | 165,012 | 0,636 | 0,522 | 0,579 | Kmn1 | kinetochore Mis12-Ndc80 network component 1 |
| A_09_P043356 | 59,646 | 122,533 | 108,953 | 323,423 | 0,487 | 0,337 | 0,412 | l(1)sc | lethal of scute |
| A_09_P012841 | 50,464 | 112,922 | 83,159 | 154,119 | 0,447 | 0,540 | 0,493 | l(3)07882 | lethal (3) 07882 |
| A_09_P014721 | 302,752 | 666,923 | 468,21 | 1230,206 | 0,454 | 0,381 | 0,417 | l(3)72Dr | lethal (3) 72Dr |
| A_09_P120605 | 196,754 | 391,354 | 178,554 | 533,174 | 0,503 | 0,335 | 0,419 | lncRNA:CR32652 | long non-coding RNA:CR32652 |
| A_09_P016091 | 73,276 | 190,221 | 81,187 | 243,152 | 0,385 | 0,334 | 0,360 | lncRNA:CR32652 | long non-coding RNA:CR32652 |
| A_09_P186245 | 179,370 | 451,459 | 223,592 | 907,812 | 0,397 | 0,246 | 0,322 | lncRNA:CR32658 | long non-coding RNA:CR32658 |
| A_09_P198135 | 137,233 | 431,606 | 177,048 | 739,032 | 0,318 | 0,240 | 0,279 | lncRNA:CR32658 | long non-coding RNA:CR32658 |
| A_09_P168405 | 108,294 | 331,404 | 140,165 | 631,451 | 0,327 | 0,222 | 0,274 | lncRNA:CR32658 | long non-coding RNA:CR32658 |
| A_09_P182880 | 13,641 | 52,697 | 23,36 | 94,699 | 0,259 | 0,247 | 0,253 | lncRNA:CR45030 | long non-coding RNA:CR45030 |
| A_09_P162655 | 51,509 | 253,992 | 65,737 | 296,53 | 0,203 | 0,222 | 0,212 | lncRNA:CR45030 | long non-coding RNA:CR45030 |
| A_09_P043416 | 160,582 | 268,915 | 251,787 | 442,968 | 0,597 | 0,568 | 0,583 | m | miniature |
| A_09_P162095 | 80,468 | 157,932 | 101,812 | 173,612 | 0,510 | 0,586 | 0,548 | mav | maverick |
| A_09_P015831 | 959,311 | 2105,455 | 1755,855 | 3226,506 | 0,456 | 0,544 | 0,500 | Mco4 | Multicopper oxidase 4 |
| A_09_P065021 | 26,066 | 66,604 | 12,084 | 39,982 | 0,391 | 0,302 | 0,347 | Met75Ca | Met75Ca |
| A_09_P028396 | 769,266 | 1232,099 | 948,186 | 2089,95 | 0,624 | 0,454 | 0,539 | metl | methyltransferase-like |
| A_09_P030241 | 125,968 | 183,941 | 151,515 | 246,165 | 0,685 | 0,616 | 0,650 | mia | meiosis I arrest |
| A_09_P072446 | 56,235 | 87,375 | 52,575 | 126,018 | 0,644 | 0,417 | 0,530 | mil | milkah |
| A_09_P213030 | 44,369 | 82,866 | 42,524 | 107,382 | 0,535 | 0,396 | 0,466 | mil | milkah |
| A_09_P006196 | 40,819 | 67,966 | 24,166 | 77,990 | 0,601 | 0,310 | 0,455 | mms4 | Methyl methanesulfonate sensitivity 4 |
| A_09_P027391 | 252,072 | 384,111 | 305,484 | 588,918 | 0,656 | 0,519 | 0,587 | mRpS17 | mitochondrial ribosomal protein S17 |
| A_09_P116945 | 48,573 | 98,572 | 75,123 | 133,192 | 0,493 | 0,564 | 0,528 | Msh6 | Msh6 |
| A_09_P168960 | 45072,355 | 69100,49 | 74968,77 | 121756,28 | 0,652 | 0,616 | 0,634 | mt:CoIII | mitochondrial Cytochrome c oxidase subunit III |
| A_09_P105955 | 69,125 | 183,777 | 87,165 | 197,63 | 0,376 | 0,441 | 0,409 | mus304 | mutagen-sensitive 304 |
| A_09_P040561 | 295,069 | 509,204 | 456,571 | 713,884 | 0,579 | 0,640 | 0,610 | Naa20A | N(alpha)-acetyltransferase 20 A |
| A_09_P006261 | 157,777 | 268,282 | 236,57 | 388,051 | 0,588 | 0,610 | 0,599 | ND-B14 | NADH dehydrogenase (ubiquinone) B14 subunit |
| A_09_P078591 | 237,186 | 509,508 | 796,900 | 1597,457 | 0,466 | 0,499 | 0,482 | Ndg | Nidogen/entactin |
| A_09_P013651 | 24,106 | 64,164 | 30,081 | 53,731 | 0,376 | 0,560 | 0,468 | NimA | Nimrod A |
| A_09_P030261 | 1135,35 | 2065,162 | 1818,861 | 3215,177 | 0,550 | 0,566 | 0,558 | noi | noisette |
| A_09_P072696 | 421,692 | 674,078 | 521,784 | 927,225 | 0,626 | 0,563 | 0,594 | nom | numerous disordered muscles |
| A_09_P031456 | 19,336 | 58,432 | 19,154 | 69,082 | 0,331 | 0,277 | 0,304 | nompA | no mechanoreceptor potential A |
| A_09_P055851 | 514,564 | 780,725 | 817,174 | 1244,654 | 0,659 | 0,657 | 0,658 | Nprl3 | Nitrogen permease regulator-like 3 |
| A_09_P000921 | 38,767 | 121,834 | 63,052 | 118,629 | 0,318 | 0,532 | 0,425 | obst-H | obstructor-H |
| A_09_P077356 | 496,575 | 736,764 | 856,059 | 1692,199 | 0,674 | 0,506 | 0,590 | Optix | Optix |
| A_09_P090060 | 841,714 | 1396,668 | 1456,922 | 2845,964 | 0,603 | 0,512 | 0,557 | Optix | Optix |
| A_09_P045136 | 42,46 | 85,243 | 54,91 | 112,735 | 0,498 | 0,487 | 0,493 | Optix | Optix |
| A_09_P001936 | 33,790 | 1178,046 | 49,358 | 2065,525 | 0,029 | 0,024 | 0,026 | Or19b | Odorant receptor 19b |
| A_09_P078556 | 25,031 | 62,08 | 35,994 | 64,09 | 0,403 | 0,562 | 0,482 | Or23a | Odorant receptor 23a |
| A_09_P072436 | 5,741 | 78,555 | 5,284 | 161,291 | 0,073 | 0,033 | 0,053 | Or98a | Odorant receptor 98a |
| A_09_P019606 | 514,481 | 788,892 | 728,971 | 1209,907 | 0,652 | 0,603 | 0,627 | Oscillin | Oscillin |
| A_09_P211965 | 20304,248 | 36606,953 | 26340,684 | 49462,992 | 0,555 | 0,533 | 0,544 | p47 | p47 |
| A_09_P024766 | 18692,627 | 34142,168 | 27457,582 | 52557,680 | 0,547 | 0,522 | 0,535 | p47 | p47 |
| A_09_P215965 | 5,648 | 32,796 | 4,385 | 28,332 | 0,172 | 0,155 | 0,163 | p47 | p47 |
| A_09_P031221 | 1620,166 | 2776,599 | 2249,245 | 3859,101 | 0,584 | 0,583 | 0,583 | P5cr | Pyrroline 5-carboyxlate reductase |
| A_09_P117435 | 197,651 | 424,196 | 456,547 | 733,774 | 0,466 | 0,622 | 0,544 | pbl | pebble |
| A_09_P207675 | 867,886 | 1389,901 | 996,046 | 2009,848 | 0,624 | 0,496 | 0,560 | Pcd | pterin-4a-carbinolamine dehydratase |
| A_09_P044006 | 58,308 | 95,83 | 46,588 | 106,176 | 0,608 | 0,439 | 0,524 | Pcp | Pupal cuticle protein |
| A_09_P004546 | 378,518 | 718,63 | 583,652 | 1384,021 | 0,527 | 0,422 | 0,474 | Pdxk | Pyridoxal kinase |
| A_09_P070226 | 28,875 | 46,782 | 31,992 | 71,583 | 0,617 | 0,447 | 0,532 | Pebp1 | Phosphatidylethanolamine-binding protein 1 |
| A_09_P103830 | 70,771 | 153,642 | 193,897 | 303,511 | 0,461 | 0,639 | 0,550 | PEK | pancreatic eIF-2alpha kinase |
| A_09_P018961 | 1585,261 | 3238,007 | 3256,300 | 5028,448 | 0,490 | 0,648 | 0,569 | Pgant2 | Polypeptide N-Acetylgalactosaminyltransferase 2 |
| A_09_P163270 | 569,874 | 1125,984 | 942,757 | 1912,947 | 0,506 | 0,493 | 0,499 | Pka-R2 | Protein kinase, cAMP-dependent, regulatory subunit type 2 |
| A_09_P013336 | 58,052 | 128,03 | 111,042 | 224,21 | 0,453 | 0,495 | 0,474 | Pms2 | Pms2 |
| A_09_P212195 | 5,434 | 34,829 | 32,135 | 62,079 | 0,156 | 0,518 | 0,337 | Poxm | Pox meso |
| A_09_P074021 | 798,867 | 1286,343 | 1489,036 | 2228,913 | 0,621 | 0,668 | 0,645 | prd1 | pruning defect 1 |
| A_09_P028206 | 84,913 | 173,501 | 133,795 | 271,710 | 0,489 | 0,492 | 0,491 | Psf1 | Psf1 |
| A_09_P190880 | 122,37 | 315,626 | 124,296 | 285,667 | 0,388 | 0,435 | 0,411 | Ptp99A | Protein tyrosine phosphatase 99A |
| A_09_P172785 | 72,098 | 183,353 | 71,375 | 246,016 | 0,393 | 0,290 | 0,342 | Pura | Puratrophin-1-like |
| A_09_P029461 | 508,95 | 930,087 | 864,733 | 1601,631 | 0,547 | 0,540 | 0,544 | Pxn | Peroxidasin |
| A_09_P162475 | 224,835 | 355,887 | 297,109 | 476,606 | 0,632 | 0,623 | 0,628 | qkr58E-3 | quaking related 58E-3 |
| A_09_P065496 | 93,389 | 191,578 | 287,232 | 484,529 | 0,487 | 0,593 | 0,540 | qsm | quasimodo |
| A_09_P052086 | 8772,759 | 13517,841 | 12510,553 | 24202,238 | 0,649 | 0,517 | 0,583 | Rala | Ras-like protein A |
| A_09_P181490 | 6093,480 | 9532,755 | 8981,603 | 17954,812 | 0,639 | 0,500 | 0,570 | Rala | Ras-like protein A |
| A_09_P203680 | 85,818 | 148,184 | 64,862 | 123,126 | 0,579 | 0,527 | 0,553 | raw | raw |
| A_09_P021036 | 53,141 | 147,086 | 90,558 | 311,924 | 0,361 | 0,290 | 0,326 | Rcd-1r | Rcd-1 related |
| A_09_P169535 | 810,804 | 1479,243 | 1071,505 | 1896,069 | 0,548 | 0,565 | 0,557 | Rdl | Resistant to dieldrin |
| A_09_P021586 | 419,099 | 632,894 | 585,134 | 963,727 | 0,662 | 0,607 | 0,635 | RfC3 | Replication factor C subunit 3 |
| A_09_P046866 | 147,531 | 267,886 | 99,740 | 439,588 | 0,551 | 0,227 | 0,389 | RpL37b | Ribosomal protein L37b |
| A_09_P113845 | 58,092 | 118,668 | 26,310 | 133,816 | 0,490 | 0,197 | 0,343 | RpL37b | Ribosomal protein L37b |
| A_09_P061436 | 287,076 | 449,367 | 443,666 | 830,996 | 0,639 | 0,534 | 0,586 | Rpt6R | Regulatory particle triple-A ATPase 6-related |
| A_09_P178910 | 242,563 | 374,480 | 375,050 | 740,859 | 0,648 | 0,506 | 0,577 | Rpt6R | Regulatory particle triple-A ATPase 6-related |
| A_09_P164520 | 70,545 | 139,462 | 137,531 | 252,489 | 0,506 | 0,545 | 0,525 | Rpt6R | Regulatory particle triple-A ATPase 6-related |
| A_09_P044491 | 105,197 | 192,141 | 155,967 | 281,870 | 0,547 | 0,553 | 0,550 | rt | rotated abdomen |
| A_09_P141955 | 1040,547 | 2304,837 | 2253,427 | 3598,517 | 0,451 | 0,626 | 0,539 | Sema1a | Semaphorin 1a |
| A_09_P065586 | 58,75 | 127,521 | 184,012 | 278,038 | 0,461 | 0,662 | 0,561 | Sema5c | Semaphorin 5c |
| A_09_P019456 | 279,8 | 441,588 | 410,644 | 636,795 | 0,634 | 0,645 | 0,639 | senju | senju |
| A_09_P044706 | 81,777 | 118,326 | 106,953 | 166,861 | 0,691 | 0,641 | 0,666 | shf | shifted |
| A_09_P000171 | 215,175 | 974,722 | 127,422 | 1743,964 | 0,221 | 0,073 | 0,147 | SIFa | SIFamide |
| A_09_P073521 | 47,103 | 178,100 | 154,109 | 312,810 | 0,264 | 0,493 | 0,379 | Skeletor | Skeletor |
| A_09_P078216 | 130,955 | 239,172 | 126,655 | 207,874 | 0,548 | 0,609 | 0,578 | SkpB | SKP1-related B |
| A_09_P007601 | 289,752 | 427,473 | 563,113 | 871,138 | 0,678 | 0,646 | 0,662 | SmydA-1 | SET and MYND domain containing, arthropod-specific, member 1 |
| A_09_P008566 | 208,74 | 416,102 | 359,127 | 626,806 | 0,502 | 0,573 | 0,537 | SmydA-7 | SET and MYND domain containing, arthropod-specific, member 7 |
| A_09_P069841 | 74,304 | 121,591 | 92,321 | 162,12 | 0,611 | 0,569 | 0,590 | Snmp1 | Sensory neuron membrane protein 1 |
| A_09_P060661 | 16,163 | 39,259 | 41,839 | 83,931 | 0,412 | 0,498 | 0,455 | snu | snustorr |
| A_09_P036121 | 314,403 | 531,982 | 651,871 | 991,844 | 0,591 | 0,657 | 0,624 | spartin | spartin |
| A_09_P044861 | 306,462 | 586,901 | 374,306 | 664,03 | 0,522 | 0,564 | 0,543 | spn-A | spindle A |
| A_09_P160755 | 319,071 | 865,297 | 604,137 | 1196,581 | 0,369 | 0,505 | 0,437 | SPoCk | Secretory Pathway Calcium atpase |
| A_09_P009551 | 12,151 | 45,591 | 14,332 | 41,022 | 0,267 | 0,349 | 0,308 | st | scarlet |
| A_09_P010626 | 879,269 | 1667,497 | 1253,931 | 3241,312 | 0,527 | 0,387 | 0,457 | Su(P) | Suppressor of ref(2)P sterility |
| A_09_P192255 | 44,176 | 102,623 | 68,436 | 195,167 | 0,430 | 0,351 | 0,391 | sxc | super sex combs |
| A_09_P153445 | 1935,82 | 3182,817 | 2043,886 | 3380,037 | 0,608 | 0,605 | 0,606 | Syn | Synapsin |
| A_09_P218350 | 275,311 | 440,523 | 467,119 | 705,575 | 0,625 | 0,662 | 0,644 | Tango6 | Transport and Golgi organization 6 |
| A_09_P023296 | 245,396 | 400,959 | 372,764 | 568,432 | 0,612 | 0,656 | 0,634 | Tango6 | Transport and Golgi organization 6 |
| A_09_P003641 | 173,006 | 318,622 | 207,341 | 368,719 | 0,543 | 0,562 | 0,553 | tau | tau |
| A_09_P021276 | 33,162 | 74,653 | 34,068 | 105,988 | 0,444 | 0,321 | 0,383 | TbCMF46 | TbCMF46 |
| A_09_P058491 | 40,843 | 62,977 | 110,494 | 241,878 | 0,649 | 0,457 | 0,553 | Tdc2 | Tyrosine decarboxylase 2 |
| A_09_P076426 | 70,863 | 159,446 | 118,233 | 311,424 | 0,444 | 0,380 | 0,412 | teq | Tequila |
| A_09_P199330 | 99,427 | 197,922 | 101,379 | 345,844 | 0,502 | 0,293 | 0,398 | teq | Tequila |
| A_09_P105375 | 46,424 | 112,730 | 52,302 | 124,882 | 0,412 | 0,419 | 0,415 | TkR86C | Tachykinin-like receptor at 86C |
| A_09_P011106 | 46,854 | 170,564 | 92,704 | 232,400 | 0,275 | 0,399 | 0,337 | TkR86C | Tachykinin-like receptor at 86C |
| A_09_P194100 | 874,347 | 1666,449 | 1771,717 | 2817,56 | 0,525 | 0,629 | 0,577 | trv | trivet |
| A_09_P056546 | 110,848 | 200,207 | 193,094 | 406,459 | 0,554 | 0,475 | 0,514 | Tsp68C | Tetraspanin 68C |
| A_09_P112185 | 43,843 | 92,435 | 38,626 | 200,806 | 0,474 | 0,192 | 0,333 | Tsp68C | Tetraspanin 68C |
| A_09_P009876 | 807,88 | 1329,842 | 1208,197 | 2006,909 | 0,608 | 0,602 | 0,605 | tub | tube |
| A_09_P023866 | 945,209 | 1584,775 | 2030,523 | 3420,382 | 0,596 | 0,594 | 0,595 | twit | target of wit |
| A_09_P025041 | 4372,284 | 36978,727 | 6172,02 | 42156,965 | 0,118 | 0,146 | 0,132 | udd | under-developed |
| A_09_P135835 | 125,732 | 373,232 | 193,751 | 407,786 | 0,337 | 0,475 | 0,406 | UGP | UGP |
| A_09_P054301 | 238,287 | 738,275 | 401,932 | 1114,524 | 0,323 | 0,361 | 0,342 | UGP | UGP |
| A_09_P026026 | 35,339 | 67,577 | 25,949 | 68,051 | 0,523 | 0,381 | 0,452 | Ugt49C1 | UDP-glycosyltransferase family 49 member C1 |
| A_09_P011396 | 556,491 | 959,392 | 1097,723 | 2415,325 | 0,580 | 0,454 | 0,517 | upd1 | unpaired 1 |
| A_09_P193722 | 5,335 | 43,282 | 4,531 | 117,095 | 0,123 | 0,039 | 0,081 | Usp10 | Ubiquitin specific protease 10 |
| A_09_P054146 | 52,405 | 153,867 | 110,867 | 273,238 | 0,341 | 0,406 | 0,373 | ValRS-m | Valyl-tRNA synthetase, mitochondrial |
| A_09_P056616 | 642,154 | 1181,736 | 1577,386 | 2725,896 | 0,543 | 0,579 | 0,561 | vir-1 | virus-induced RNA 1 |
| A_09_P121180 | 34,909 | 112,934 | 97,802 | 227,777 | 0,309 | 0,429 | 0,369 | WRNexo | WRN exonuclease |
| A_09_P068851 | 29,518 | 108,878 | 79,321 | 192,252 | 0,271 | 0,413 | 0,342 | WRNexo | WRN exonuclease |
| A_09_P064411 | 55,826 | 98,44 | 66,12 | 104,709 | 0,567 | 0,631 | 0,599 | yellow-d | yellow-d |
| A_09_P010096 | 124,446 | 216,777 | 165,445 | 348,880 | 0,574 | 0,474 | 0,524 | Z600 | Z600 |
| A_09_P061131 | 228,449 | 515,298 | 327,409 | 893,209 | 0,443 | 0,367 | 0,405 | Zip99C | Zinc/iron regulated transporter-related protein 99C |
| A_09_P065281 | 24,782 | 64,635 | 35,481 | 64,912 | 0,383 | 0,547 | 0,465 | ZnT35C | Zinc transporter 35C |

**Table S5. List of the genetic modifiers of Tau-toxicity identified so far in *Drosophila* using the REP**

| *Drosophila* | | Human orthologs | |
| --- | --- | --- | --- |
| Gene Symbol | Gene Name | Gene Symbol | Gene Name |
| *alphaTub67C* | alpha-Tubulin at 67C | *TUBA1A* | tubulin alpha 1a |
|  |  | *TUBA3C* | tubulin alpha 3c |
|  |  | *TUBA4A* | tubulin alpha 4a |
|  |  | *TUBAL3* | tubulin alpha like 3 |
| *alphaTub84B* | alpha-Tubulin at 84B | *TUBA3D* | tubulin alpha 3d |
| ***amos*** | **absent MD neurons and olfactory sensilla** | ***ATOH1*** | **atonal bHLH transcription factor 1** |
| *Amph* | Amphiphysin | *AMPH* | amphiphysin |
|  |  | *BIN1* | bridging integrator 1 |
| *AMPKalpha* | AMP-activated protein kinase alpha subunit | *PRKAA2* | protein kinase AMP-activated catalytic subunit alpha 2 |
| *aop* | anterior open | *ETV6* | ETS variant 6 |
| *Apf* | diadenosine tetraphosphate hydrolase | *NUDT2* | nudix hydrolase 2 |
| *arm* | armadillo | *CTNNB1* | catenin beta 1 |
| *armi* | armitage | *MOV10L1* | Mov10 like RISC complex RNA helicase 1 |
| *Arp10* | Actin-related protein 10 | *ACTR10* | actin related protein 10 |
| *Arv1* | ACAT-related protein required for viability 1 | *ARV1* | ARV1 homolog, fatty acid homeostasis modulator |
| *asp* | abnormal spindle | *ASPM* | abnormal spindle microtubule assembly |
| *Atg6* | Autophagy-related 6 | *BECN1* | beclin 1 |
| *Atpalpha* | Na pump alpha subunit | *ATP1A1* | ATPase Na+/K+ transporting subunit alpha 1 |
|  |  | *ATP1A3* | ATPase Na+/K+ transporting subunit alpha 3 |
| ***Atx2*** | **Ataxin-2** | ***ATXN2*** | **ataxin 2** |
|  |  | ***ATXN2L*** | **ataxin 2 like** |
| *Bacc* | Bacchus | */* | / |
| *bbg* | big bang | *IL16* | interleukin 16 |
| *beta-Spec* | beta Spectrin | *SPTBN1* | spectrin beta, non-erythrocytic 1 |
|  |  | *SPTBN2* | spectrin beta, non-erythrocytic 2 |
| *bic* | bicaudal | *BTF3* | basic transcription factor 3 |
|  |  | *BTF3L4* | basic transcription factor 3 like 4 |
| *bnl* | branchless | *FGF16* | fibroblast growth factor 16 |
|  |  | *FGF20* | fibroblast growth factor 20 |
| *bon* | bonus | *TRIM24* | tripartite motif containing 24 |
|  |  | *TRIM33* | tripartite motif containing 33 |
| *bru1* | bruno 1 | *CELF1* | CUGBP Elav-like family member 1 |
|  |  | *CELF2* | CUGBP Elav-like family member 2 |
| *brun* | brunelleschi | *TRAPPC9* | trafficking protein particle complex 9 |
| ***bw*** | **brown** | ***ABCG4*** | **ATP binding cassette subfamily G member 4** |
|  |  | ***ABCG5*** | **ATP binding cassette subfamily G member 5** |
|  |  | ***ABCG8*** | **ATP binding cassette subfamily G member 8** |
| *Cam* | Calmodulin | *CALM3* | calmodulin 3 |
| *CaMKI* | Calcium/calmodulin-dependent protein kinase I | *CAMK1D* | calcium/calmodulin dependent protein kinase ID |
| *CaMKII* | Calcium/calmodulin-dependent protein kinase II | *CAMK2D* | calcium/calmodulin dependent protein kinase II delta |
| *cana* | CENP-ana | *CENPE* | centromere protein E |
| *CASK* | CASK | *CASK* | calcium/calmodulin dependent serine protein kinase |
| *CCT7* | Chaperonin containing TCP1 subunit 7 | *CCT7* | chaperonin containing TCP1 subunit 7 |
| *cdi* | center divider | *TESK2* | testis associated actin remodelling kinase 2 |
| *Cdk5* | Cyclin-dependent kinase 5 | *CDK5* | cyclin dependent kinase 5 |
| *Cdk5alpha* | Cdk5 activator-like protein | *CDK5R1* | cyclin dependent kinase 5 regulatory subunit 1 |
| ***CG10077*** | **-** | ***DDX5*** | **DEAD-box helicase 5** |
| *CG10889* | - | *ZC3H12A* | zinc finger CCCH-type containing 12A |
| *CG10927* | - | *ADAT3* | adenosine deaminase tRNA specific 3 |
| *CG10979* | - | *ZNF800* | zinc finger protein 800 |
| *CG12395* | - | *SPEF1* | sperm flagellar 1 |
| *CG12935* | - | *TMEM223* | transmembrane protein 223 |
| *CG14184* | - | *LAMTOR1* | late endosomal/lysosomal adaptor, MAPK and MTOR activator 1 |
| *CG14621* | - | *SLC35E1* | solute carrier family 35 member E1 |
| *CG15629* | - | *RDH10* | retinol dehydrogenase 10 |
| *CG17327* | - | *PTRH2* | peptidyl-tRNA hydrolase 2 |
| *CG1806* | - | *SSPN* | sarcospan |
| *CG18508* | - | *C18orf32* | chromosome 18 open reading frame 32 |
| *CG30015* | - | */* | / |
| *CG31259* | - | *TMEM135* | transmembrane protein 135 |
| *CG31886* | - | */* | / |
| *CG32809* | - | *KIAA1217* | KIAA1217 |
| *CG3500* | - | *TEX261* | testis expressed 261 |
| *CG3511* | - | *PPWD1* | peptidylprolyl isomerase domain and WD repeat containing 1 |
| *CG3735* | - | *DIEXF* | / |
| *CG3808* | - | *TRMT2A* | tRNA methyltransferase 2 homolog A |
| *CG42724* | - | *TCERG1* | transcription elongation regulator 1 |
| ***CG42788*** | **-** | ***FRMPD4*** | **FERM and PDZ domain containing 4** |
| ***CG46385*** | **-** | ***/*** | **/** |
| *CG5500* | - | *OXLD1* | oxidoreductase like domain containing 1 |
| ***CG5830*** | **-** | ***CTDSP1*** | **CTD small phosphatase 1** |
| *CG5986* | - | *SDE2* | SDE2 telomere maintenance homolog |
| *CG6330* | - | *UPP2* | uridine phosphorylase 2 |
| *CG6418* | - | *DDX42* | DEAD-box helicase 42 |
| ***CG6701*** | **-** | ***MOV10*** | **Mov10 RISC complex RNA helicase** |
| *CG6873* | - | *CFL2* | cofilin 2 |
| *CG7231* | - | *FAM151B* | family with sequence similarity 151 member B |
| *CG7896* | - | *IGFALS* | insulin like growth factor binding protein acid labile subunit |
| *CG7970* | - | *PXMP2* | peroxisomal membrane protein 2 |
| *CG8086* | - | *ODF3* | outer dense fiber of sperm tails 3 |
| *CG8108* | - | *CIZ1* | CDKN1A interacting zinc finger protein 1 |
| *CG8664* | - | */* | / |
| *CG8785* | - | *SLC36A4* | solute carrier family 36 member 4 |
| *chb* | chromosome bows | *CLASP1* | cytoplasmic linker associated protein 1 |
| *Chd64* | Chd64 | *TAGLN* | transgelin |
|  |  | *TAGLN2* | transgelin 2 |
|  |  | *TAGLN3* | transgelin 3 |
| *cher* | cheerio | *FLNA* | filamin A |
| *cic* | capicua | *CIC* | capicua transcriptional repressor |
| ***cindr*** | **CIN85 and CD2AP related** | ***SH3KBP1*** | **SH3 domain containing kinase binding protein 1** |
| ***CLIP-190*** | **Cytoplasmic linker protein 190** | ***CLIP1*** | **CAP-Gly domain containing linker protein 1** |
|  |  | ***CLIP2*** | **CAP-Gly domain containing linker protein 2** |
| ***comm*** | **commissureless** | ***PRRG4*** | **proline rich and Gla domain 4** |
| *cpx* | complexin | *CPLX1* | complexin 1 |
| *crp* | cropped | *TFAP4* | transcription factor AP-4 |
| *Csp* | Cysteine string protein | *DNAJC5* | DnaJ heat shock protein family (Hsp40) member C5 |
| *csul* | capsuleen | *PRMT5* | protein arginine methyltransferase 5 |
| *Cwc25* | Cwc25 | *CWC25* | CWC25 spliceosome associated protein homolog |
| *CycE* | Cyclin E | *CCNE1* | cyclin E1 |
| *CycJ* | Cyclin J | *CCNJ* | cyclin J |
| ***Cyp301a1*** | **Cyp301a1** | ***CYP27A1*** | **cytochrome P450 family 27 subfamily A member 1** |
| *Dab* | Disabled | *DAB1* | DAB adaptor protein 1 |
|  |  | *DAB2* | DAB adaptor protein 2 |
| *dally* | division abnormally delayed | *GPC5* | glypican 5 |
| *DCTN1-p150* | Dynactin 1, p150 subunit | *DCTN1* | dynactin subunit 1 |
| *DCTN2-p50* | Dynactin 2, p50 subunit | *DCTN2* | dynactin subunit 2 |
| *DCTN4-p62* | Dynactin 4, p62 subunit | *DCTN4* | dynactin subunit 4 |
| ***DCTN5-p25*** | **Dynactin 5, p25 subunit** | ***DCTN5*** | **dynactin subunit 5** |
| *Ddr* | Discoidin domain receptor | *DDR2* | discoidin domain receptor tyrosine kinase 2 |
| *Dhc93AB* | Dynein heavy chain at 93AB | *DNAH9* | dynein axonemal heavy chain 9 |
| *Diap1* | Death-associated inhibitor of apoptosis 1 | *BIRC2* | baculoviral IAP repeat containing 2 |
|  |  | *BIRC3* | baculoviral IAP repeat containing 3 |
| *Diap2* | Death-associated inhibitor of apoptosis 2 | *BIRC2* | baculoviral IAP repeat containing 2 |
| *dlg1* | discs large 1 | *DLG1* | discs large MAGUK scaffold protein 1 |
| *Dlic* | Dynein light intermediate chain | *DYNC1LI1* | dynein cytoplasmic 1 light intermediate chain 1 |
|  |  | *DYNC1LI2* | dynein cytoplasmic 1 light intermediate chain 2 |
| *Dmtn* | Dementin | *TMCC1* | transmembrane and coiled-coil domain family 1 |
|  |  | *TMCC2* | transmembrane and coiled-coil domain family 2 |
| *DnaJ-1* | DnaJ-like-1 | *DNAJB4* | DnaJ heat shock protein family (Hsp40) member B4 |
| *dop* | drop out | *MAST3* | microtubule associated serine/threonine kinase 3 |
|  |  | *MAST4* | microtubule associated serine/threonine kinase family member 4 |
| *Dp* | DP transcription factor | *TFDP1* | transcription factor Dp-1 |
|  |  | *TFDP2* | transcription factor Dp-2 |
| *dpr1* | defective proboscis extension response 1 | */* | / |
| *dpr18* | defective proboscis extension response 18 | */* | / |
| *Drak* | Death-associated protein kinase related | *STK17B* | serine/threonine kinase 17b |
| ***E(bx)*** | **Enhancer of bithorax** | ***BPTF*** | **bromodomain PHD finger transcription factor** |
| ***e(y)3*** | **enhancer of yellow 3** | ***PHF10*** | **PHD finger protein 10** |
| *eEF1alpha1* | eukaryotic translation elongation factor 1 alpha 1 | *EEF1A2* | eukaryotic translation elongation factor 1 alpha 2 |
| *eIF4EHP* | eukaryotic translation initiation factor 4E homologous protein | *EIF4E2* | eukaryotic translation initiation factor 4E family member 2 |
| *EloB* | Elongin B | *ELOB* | elongin B |
| *ena* | enabled | *ENAH* | ENAH actin regulator |
| *ens* | ensconsin | *MAP7* | microtubule associated protein 7 |
|  |  | *MAP7D1* | MAP7 domain containing 1 |
|  |  | *MAP7D2* | MAP7 domain containing 2 |
|  |  | *MAP7D3* | MAP7 domain containing 3 |
| *Eph* | Eph receptor tyrosine kinase | *EPHB1* | EPH receptor B1 |
| *eRF1* | eukaryotic translation release factor 1 | *ETF1* | eukaryotic translation termination factor 1 |
| *eRF3* | eukaryotic translation release factor 3 | *GSPT1* | G1 to S phase transition 1 |
| *Fak* | Focal adhesion kinase | *PTK2* | protein tyrosine kinase 2 |
| *fbl* | fumble | *PANK3* | pantothenate kinase 3 |
| ***Fem-1*** | **Fem-1** | ***FEM1B*** | **fem-1 homolog B** |
| *Fer1* | 48 related 1 | *PTF1A* | pancreas associated transcription factor 1a |
| *Fer1HCH* | Ferritin 1 heavy chain homologue | *FTH1* | ferritin heavy chain 1 |
|  |  | *FTHL17* | ferritin heavy chain like 17 |
|  |  | *FTMT* | ferritin mitochondrial |
| *Fit1* | Fermitin 1 | *FERMT2* | fermitin family member 2 |
| *Fit2* | Fermitin 2 | *FERMT2* | fermitin family member 2 |
| *Flo2* | Flotillin 2 | *FLOT2* | flotillin 2 |
| *Fmr1* | Fmr1 | *FMR1* | fragile X mental retardation 1 |
|  |  | *FXR1* | FMR1 autosomal homolog 1 |
| *fne* | found in neurons | *ELAVL2* | ELAV like RNA binding protein 2 |
|  |  | *ELAVL4* | ELAV like RNA binding protein 4 |
| *frc* | fringe connection | *SLC35D1* | solute carrier family 35 member D1 |
| ***fru*** | **fruitless** | ***ZBTB1*** | **zinc finger and BTB domain containing 1** |
|  |  | ***ZBTB24*** | **zinc finger and BTB domain containing 24** |
|  |  | ***ZBTB39*** | **zinc finger and BTB domain containing 39** |
|  |  | ***ZBTB45*** | **zinc finger and BTB domain containing 45** |
| *fry* | furry | *FRYL* | FRY like transcription coactivator |
| *fs(1)h* | female sterile (1) homeotic | *BRD2* | bromodomain containing 2 |
|  |  | *BRD3* | bromodomain containing 3 |
|  |  | *BRD4* | bromodomain containing 4 |
|  |  | *BRDT* | bromodomain testis associated |
| *Fs(2)Ket* | Female sterile (2) Ketel | *KPNB1* | karyopherin subunit beta 1 |
| *fzy* | fizzy | *CDC20* | cell division cycle 20 |
| *g* | garnet | *AP3D1* | adaptor related protein complex 3 subunit delta 1 |
| *Gabat* | gamma-aminobutyric acid transaminase | *ABAT* | 4-aminobutyrate aminotransferase |
| *garz* | gartenzwerg | *GBF1* | golgi brefeldin A resistant guanine nucleotide exchange factor 1 |
| *Gbs-70E* | Glycogen binding subunit 70E | *PPP1R3C* | protein phosphatase 1 regulatory subunit 3C |
| *Gdi* | GDP dissociation inhibitor | *GDI1* | GDP dissociation inhibitor 1 |
| ***Glut1*** | **Glucose transporter 1** | ***SLC2A3*** | **solute carrier family 2 member 3** |
| *gpp* | grappa | *DOT1L* | DOT1 like histone lysine methyltransferase |
| ***Gr47b*** | **Gustatory receptor 47b** | ***/*** | **/** |
| *GstS1* | Glutathione S transferase S1 | *HPGDS* | hematopoietic prostaglandin D synthase |
| ***h*** | **hairy** | ***HES4*** | **hes family bHLH transcription factor 4** |
| *haf* | hattifattener | *TRIL* | TLR4 interactor with leucine rich repeats |
| ***hdc*** | **headcase** | ***HECA*** | **hdc homolog, cell cycle regulator** |
| *hep* | hemipterous | *MAP2K7* | mitogen-activated protein kinase kinase 7 |
| *heph* | hephaestus | *PTBP1* | polypyrimidine tract binding protein 1 |
| ***Hipk*** | **Homeodomain interacting protein kinase** | ***HIPK1*** | **homeodomain interacting protein kinase 1** |
|  |  | ***HIPK2*** | **homeodomain interacting protein kinase 2** |
|  |  | ***HIPK3*** | **homeodomain interacting protein kinase 3** |
| *His2A:CG31618* | His2A:CG31618 | *HIST1H2AA* | histone cluster 1 H2A family member a |
|  |  | *HIST1H2AC* | histone cluster 1 H2A family member c |
|  |  | *HIST2H2AA4* | histone cluster 2 H2A family member a4 |
|  |  | *HIST2H2AC* | histone cluster 2 H2A family member c |
|  |  | *HIST3H2A* | histone cluster 3 H2A |
| *His2Av* | Histone H2A variant | *H2AFV* | H2A histone family member V |
|  |  | *H2AFZ* | H2A histone family member Z |
| *HisRS* | Histidyl-tRNA synthetase | *HARS* | histidyl-tRNA synthetase |
| *HnRNP-K* | Heterogeneous nuclear ribonucleoprotein K | *HNRNPK* | heterogeneous nuclear ribonucleoprotein K |
| *hop* | hopscotch | *JAK2* | Janus kinase 2 |
| *Hr39* | Hormone receptor-like in 39 | *NR6A1* | nuclear receptor subfamily 6 group A member 1 |
| *Hs6st* | Heparan sulfate 6-O-sulfotransferase | *HS6ST1* | heparan sulfate 6-O-sulfotransferase 1 |
| *Hsc70Cb* | Hsc70Cb | *HSPA4* | heat shock protein family A (Hsp70) member 4 |
| *IP3K1* | Inositol 1,4,5-triphosphate kinase 1 | *ITPKA* | inositol-trisphosphate 3-kinase A |
| *jar* | jaguar | *MYO6* | myosin VI |
| ***jim*** | **jim** | ***ZNF133*** | **zinc finger protein 133** |
|  |  | ***ZNF343*** | **zinc finger protein 343** |
|  |  | ***ZNF460*** | **zinc finger protein 460** |
|  |  | ***ZNF708*** | **zinc finger protein 708** |
|  |  | ***ZNF724*** | **zinc finger protein 724** |
| *jing* | jing | *AEBP2* | AE binding protein 2 |
| *jumu* | jumeau | *FOXN1* | forkhead box N1 |
|  |  | *FOXN4* | forkhead box N4 |
| *kay* | kayak | *FOS* | Fos proto-oncogene, AP-1 transcription factor subunit |
|  |  | *FOSL1* | FOS like 1, AP-1 transcription factor subunit |
|  |  | *FOSL2* | FOS like 2, AP-1 transcription factor subunit |
| *kibra* | kibra | *WWC1* | WW and C2 domain containing 1 |
|  |  | *WWC2* | WW and C2 domain containing 2 |
| *Klp61F* | Kinesin-like protein at 61F | *KIF11* | kinesin family member 11 |
| *krz* | kurtz | *ARRB1* | arrestin beta 1 |
|  |  | *ARRB2* | arrestin beta 2 |
| *ksr* | kinase suppressor of ras | *KSR2* | kinase suppressor of ras 2 |
| ***kuz*** | **kuzbanian** | ***ADAM10*** | **ADAM metallopeptidase domain 10** |
| *l(2)05287* | lethal (2) 05287 | *WDR75* | WD repeat domain 75 |
| *l(3)j6A6* | lethal (3) j6A6 | */* | / |
| *l(3)j11B2* | lethal (3) j11B2 | */* | / |
| *l(3)L0499* | lethal (3) L0499 | */* | / |
| ***l(3)L1231*** | **lethal (3) L1231** | ***INO80D*** | **INO80 complex subunit D** |
| *l(3)L6332* | lethal (3) L6332 | */* | / |
| *l(3)neo38* | lethal (3) neo38 | *SP3* | Sp3 transcription factor |
| *Lar* | Leukocyte-antigen-related-like | *PTPRD* | protein tyrosine phosphatase receptor type D |
| *Lerp* | lysosomal enzyme receptor protein | *IGF2R* | insulin like growth factor 2 receptor |
| *lncRNA:CR31044* | long non-coding RNA::CR31044 | */* | / |
| *lncRNA:CR43650* | long non-coding RNA:CR43650 | */* | / |
| *Mad1* | Mitotic arrest-deficient 1 | *MAD1L1* | mitotic arrest deficient 1 like 1 |
| *mam* | mastermind | *MAML1* | mastermind like transcriptional coactivator 1 |
| ***Mbs*** | **Myosin binding subunit** | ***PPP1R12B*** | **protein phosphatase 1 regulatory subunit 12B** |
| *MED14* | Mediator complex subunit 14 | *MED14* | mediator complex subunit 14 |
| *mei-9* | meiotic 9 | *ERCC4* | ERCC excision repair 4, endonuclease catalytic subunit |
| *Mekk1* | Mekk1 | *MAP3K4* | mitogen-activated protein kinase kinase kinase 4 |
| *melt* | melted | *VEPH1* | ventricular zone expressed PH domain containing 1 |
| ***Meltrin*** | **Meltrin** | ***ADAM12*** | **ADAM metallopeptidase domain 12** |
| *Mi-2* | Mi-2 | *CHD3* | chromodomain helicase DNA binding protein 3 |
| *milt* | milton | *TRAK1* | trafficking kinesin protein 1 |
| *MRG15* | MORF-related gene 15 | *MORF4L1* | mortality factor 4 like 1 |
| *mub* | mushroom-body expressed | *PCBP3* | poly(rC) binding protein 3 |
| *mura* | murashka | *RNF38* | ring finger protein 38 |
| *Myd88* | Myd88 | *MYD88* | MYD88 innate immune signal transduction adaptor |
| ***nab*** | **nab** | ***NAB1*** | **NGFI-A binding protein 1** |
|  |  | ***NAB2*** | **NGFI-A binding protein 2** |
| *NC2alpha* | Negative Cofactor 2alpha | *DRAP1* | DR1 associated protein 1 |
| *Nedd8* | Nedd8 | *NEDD8* | NEDD8 ubiquitin like modifier |
| ***Nelf-E*** | **Negative elongation factor E** | ***NELFE*** | **negative elongation factor complex member E** |
| *nord* | nord | *NDNF* | neuron derived neurotrophic factor |
| *Not1* | Not1 | *CNOT1* | CCR4-NOT transcription complex subunit 1 |
| *Nrg* | Neuroglian | *NRCAM* | neuronal cell adhesion molecule |
| *Nrx-IV* | Neurexin IV | *CNTNAP2* | contactin associated protein like 2 |
| *NSD* | Nuclear receptor binding SET domain protein | *NSD3* | nuclear receptor binding SET domain protein 3 |
| ***Nsf2*** | **N-ethylmaleimide-sensitive factor 2** | ***NSF*** | **N-ethylmaleimide sensitive factor, vesicle fusing ATPase** |
| *Nuak1* | Nuak1 ortholog | *NUAK1* | NUAK family kinase 1 |
| *Nuf2* | Nuf2 | *CCDC39* | coiled-coil domain containing 39 |
| *numb* | numb | *NUMBL* | NUMB like endocytic adaptor protein |
| *Oct-TyrR* | Octopamine-Tyramine receptor | *ADRA2A* | adrenoceptor alpha 2A |
|  |  | *ADRA2B* | adrenoceptor alpha 2B |
|  |  | *ADRA2C* | adrenoceptor alpha 2C |
|  |  | *HTR1A* | 5-hydroxytryptamine receptor 1A |
|  |  | *HTR1D* | 5-hydroxytryptamine receptor 1D |
| *Orct2* | Organic cation transporter 2 | *SLC22A13* | solute carrier family 22 member 13 |
|  |  | *SLC22A4* | solute carrier family 22 member 4 |
|  |  | *SLC22A5* | solute carrier family 22 member 5 |
| *oxt* | peptide O-xylosyltransferase | *XYLT1* | xylosyltransferase 1 |
|  |  | *XYLT2* | xylosyltransferase 2 |
| *p130CAS* | p130CAS | *BCAR1* | BCAR1 scaffold protein, Cas family member |
|  |  | *NEDD9* | neural precursor cell expressed, developmentally down-regulated 9 |
| *p35* | p35 | */* | / |
| *pan* | pangolin | *TCF7L2* | transcription factor 7 like 2 |
| ***par-1*** | **par-1** | ***MARK3*** | **microtubule affinity regulating kinase 3** |
| *par-6* | par-6 | *PARD6G* | par-6 family cell polarity regulator gamma |
| *Past1* | Putative Achaete Scute Target 1 | *EHD1* | EH domain containing 1 |
| ***Pax*** | **Paxillin** | ***PXN*** | **paxillin** |
| ***pbl*** | **pebble** | ***ECT2*** | **epithelial cell transforming 2** |
| *Pdk* | Pyruvate dehydrogenase kinase | *PDK3* | pyruvate dehydrogenase kinase 3 |
| *Pdk1* | Phosphoinositide-dependent kinase 1 | *PDPK1* | 3-phosphoinositide dependent protein kinase 1 |
| ***Pdp1*** | **PAR-domain protein 1** | ***HLF*** | **HLF transcription factor, PAR bZIP family member** |
| *Piezo* | Piezo | *PIEZO2* | piezo type mechanosensitive ion channel component 2 |
| *Pka-C1* | Protein kinase, cAMP-dependent, catalytic subunit 1 | *PRKACA* | protein kinase cAMP-activated catalytic subunit alpha |
|  |  | *PRKACB* | protein kinase cAMP-activated catalytic subunit beta |
| *poe* | purity of essence | *UBR4* | ubiquitin protein ligase E3 component n-recognin 4 |
| *Prosalpha7* | Proteasome alpha7 subunit | *PSMA3* | proteasome subunit alpha 3 |
| *Prosbeta2* | Proteasome beta2 subunit | *PSMB7* | proteasome subunit beta 7 |
| *Prp8* | pre-mRNA processing factor 8 | *PRPF8* | pre-mRNA processing factor 8 |
| *Psa* | Puromycin sensitive aminopeptidase | *NPEPPS* | aminopeptidase puromycin sensitive |
| *Ptp4E* | Protein tyrosine phosphatase 4E | *PTPRB* | protein tyrosine phosphatase receptor type B |
| ***pyd*** | **polychaetoid** | ***TJP1*** | **tight junction protein 1** |
|  |  | ***TJP2*** | **tight junction protein 2** |
| ***qkr58E-3*** | **quaking related 58E-3** | ***KHDRBS1*** | **KH RNA binding domain containing, signal transduction associated 1** |
|  |  | ***KHDRBS2*** | **KH RNA binding domain containing, signal transduction associated 2** |
|  |  | ***KHDRBS3*** | **KH RNA binding domain containing, signal transduction associated 3** |
| *Rab14* | Rab14 | *RAB14* | RAB14, member RAS oncogene family |
| *Rab30* | Rab30 | *RAB30* | RAB30, member RAS oncogene family |
| *Rab3-GEF* | Rab3 GDP-GTP exchange factor | *MADD* | MAP kinase activating death domain |
| *Rad60* | Rad60 | *NFATC2IP* | nuclear factor of activated T cells 2 interacting protein |
| ***raw*** | **raw** | ***RNASEL*** | **ribonuclease L** |
| *rhea* | rhea | *TLN2* | talin 2 |
| *Rok* | Rho kinase | *ROCK2* | Rho associated coiled-coil containing protein kinase 2 |
| *RpLP0* | Ribosomal protein LP0 | *RPLP0* | ribosomal protein lateral stalk subunit P0 |
| ***RpLP1*** | **Ribosomal protein LP1** | ***RPLP1*** | **ribosomal protein lateral stalk subunit P1** |
| *Rpn9* | Regulatory particle non-ATPase 9 | *PSMD13* | proteasome 26S subunit, non-ATPase 13 |
| *RpS10a* | Ribosomal protein S10a | *RPS10* | ribosomal protein S10 |
| ***RpS21*** | **Ribosomal protein S21** | ***RPS21*** | **ribosomal protein S21** |
| *ry* | rosy | *XDH* | xanthine dehydrogenase |
| *RyR* | Ryanodine receptor | *RYR1* | ryanodine receptor 1 |
|  |  | *RYR2* | ryanodine receptor 2 |
| *Scamp* | Scamp | *SCAMP3* | secretory carrier membrane protein 3 |
| ***scb*** | **scab** | ***ITGA4*** | **integrin subunit alpha 4** |
| ***scrib*** | **scribble** | ***SCRIB*** | **scribble planar cell polarity protein** |
| *SdhB* | Succinate dehydrogenase, subunit B (iron-sulfur) | *SDHB* | succinate dehydrogenase complex iron sulfur subunit B |
| *sdt* | stardust | *MPP5* | membrane palmitoylated protein 5 |
| *SelD* | Selenide,water dikinase | *SEPHS1* | selenophosphate synthetase 1 |
| *Sf3b5* | Splicing factor 3b subunit 5 | *SF3B5* | splicing factor 3b subunit 5 |
| *sgg* | shaggy | *GSK3A* | glycogen synthase kinase 3 alpha |
|  |  | *GSK3B* | glycogen synthase kinase 3 beta |
| ***shn*** | **schnurri** | ***HIVEP2*** | **human immunodeficiency virus type I enhancer binding protein 2** |
| *Shrm* | Shroom | *SHROOM2* | shroom family member 2 |
| ***skd*** | **skuld** | ***MED13*** | **mediator complex subunit 13** |
| *S-Lap2* | Sperm-Leucylaminopeptidase 2 | *LAP3* | leucine aminopeptidase 3 |
| *sli* | slit | *SLIT3* | slit guidance ligand 3 |
| *sm* | smooth | *HNRNPL* | heterogeneous nuclear ribonucleoprotein L |
| *smash* | smallish | *LIMCH1* | LIM and calponin homology domains 1 |
|  |  | *LMO7* | LIM domain 7 |
| *SmB* | Small ribonucleoprotein particle protein SmB | *SNRPB* | small nuclear ribonucleoprotein polypeptides B and B1 |
| ***Smg5*** | **Smg5** | ***SMG5*** | **SMG5 nonsense mediated mRNA decay factor** |
| *smid* | smallminded | *NVL* | nuclear VCP like |
| ***SNF4Agamma*** | **SNF4/AMP-activated protein kinase gamma subunit** | ***PRKAG1*** | **protein kinase AMP-activated non-catalytic subunit gamma 1** |
|  |  | ***PRKAG2*** | **protein kinase AMP-activated non-catalytic subunit gamma 2** |
| *Snp* | Snipper | *ERI2* | ERI1 exoribonuclease family member 2 |
| *snRNP-U1-C* | small ribonucleoprotein particle U1 subunit C | *SNRPC* | small nuclear ribonucleoprotein polypeptide C |
| ***Socs36E*** | **Suppressor of cytokine signaling at 36E** | ***SOCS5*** | **suppressor of cytokine signaling 5** |
| *Sodh-1* | Sorbitol dehydrogenase 1 | *SORD* | sorbitol dehydrogenase |
| *Spp* | Signal peptide peptidase | *HM13* | histocompatibility minor 13 |
| *SppL* | Signal peptide peptidase-like | *SPPL3* | signal peptide peptidase like 3 |
| *Src42A* | Src oncogene at 42A | *FRK* | fyn related Src family tyrosine kinase |
| *Src64B* | Src oncogene at 64B | *FYN* | FYN proto-oncogene, Src family tyrosine kinase |
|  |  | *SRC* | SRC proto-oncogene, non-receptor tyrosine kinase |
| *stg* | string | *CDC25A* | cell division cycle 25A |
| *Stip1* | Stress induced phosphoprotein 1 | *STIP1* | stress induced phosphoprotein 1 |
| *Su(z)2* | Suppressor of zeste 2 | *PCGF2* | polycomb group ring finger 2 |
| *Syn1* | Syntrophin-like 1 | *SNTB1* | syntrophin beta 1 |
| *T3dh* | Type III alcohol dehydrogenase | *ADHFE1* | alcohol dehydrogenase iron containing 1 |
| *Tango5* | Transport and Golgi organization 5 | *VMP1* | vacuole membrane protein 1 |
| *Tao* | Tao | *TAOK1* | TAO kinase 1 |
|  |  | *TAOK3* | TAO kinase 3 |
| ***tau*** | **tau** | ***MAPT*** | **microtubule associated protein tau** |
| *Ten-m* | Tenascin major | *TENM1* | teneurin transmembrane protein 1 |
|  |  | *TENM2* | teneurin transmembrane protein 2 |
| *Tet* | Ten-Eleven Translocation (TET) family protein | *TET1* | tet methylcytosine dioxygenase 1 |
| *Thor* | Thor | *EIF4EBP1* | eukaryotic translation initiation factor 4E binding protein 1 |
|  |  | *EIF4EBP2* | eukaryotic translation initiation factor 4E binding protein 2 |
| ***Tis11*** | **Tis11 zinc finger protein** | ***ZFP36L1*** | **ZFP36 ring finger protein like 1** |
| *Tl* | Toll | *TLR1* | toll like receptor 1 |
|  |  | *TLR10* | toll like receptor 10 |
|  |  | *TLR2* | toll like receptor 2 |
|  |  | *TLR3* | toll like receptor 3 |
|  |  | *TLR4* | toll like receptor 4 |
|  |  | *TLR6* | toll like receptor 6 |
|  |  | *TLR7* | toll like receptor 7 |
|  |  | *TLR9* | toll like receptor 9 |
| *tou* | toutatis | *BAZ2B* | bromodomain adjacent to zinc finger domain 2B |
| *Tsp96F* | Tetraspanin 96F | *CD81* | CD81 molecule |
| *ttv* | tout-velu | *EXT1* | exostosin glycosyltransferase 1 |
| *twe* | twine | *CDC25A* | cell division cycle 25A |
|  |  | *CDC25B* | cell division cycle 25B |
|  |  | *CDC25C* | cell division cycle 25C |
| *Uba1* | Ubiquitin activating enzyme 1 | *UBA1* | ubiquitin like modifier activating enzyme 1 |
| *Uck* | Uridine-cytidine kinase | *UCK2* | uridine-cytidine kinase 2 |
| *unc-45* | uncoordinated 45 | *UNC45B* | unc-45 myosin chaperone B |
| *Unc-89* | Unc-89 | *SPEG* | striated muscle enriched protein kinase |
| ***Usp10*** | **Ubiquitin specific protease 10** | ***USP10*** | **ubiquitin specific peptidase 10** |
| *Usp47* | Ubiquitin specific protease 47 | *USP47* | ubiquitin specific peptidase 47 |
| *Vap33* | VAMP-associated protein 33kDa | *VAPB* | VAMP associated protein B and C |
| *Vha14-1* | Vacuolar H[+] ATPase 14kD subunit 1 | *ATP6V1F* | ATPase H+ transporting V1 subunit F |
| *Vha16-1* | Vacuolar H[+] ATPase 16kD subunit 1 | *ATP6V0C* | ATPase H+ transporting V0 subunit c |
| *Vha36-1* | Vacuolar H[+] ATPase 36kD subunit 1 | *ATP6V1D* | ATPase H+ transporting V1 subunit D |
| *Vha44* | Vacuolar H[+] ATPase 44kD subunit | *ATP6V1C1* | ATPase H+ transporting V1 subunit C1 |
| *vnc* | variable nurse cells | *NAA10* | N(alpha)-acetyltransferase 10, NatA catalytic subunit |
| ***w*** | **white** | ***ABCG2*** | **ATP binding cassette subfamily G member 2** |
| *wdb* | widerborst | *PPP2R5E* | protein phosphatase 2 regulatory subunit B'epsilon |
| *wde* | windei | *ATF7IP* | activating transcription factor 7 interacting protein |
| *Wee1* | Wee1 kinase | *WEE1* | WEE1 G2 checkpoint kinase |
| *wun* | wunen | *PLPP1* | phospholipid phosphatase 1 |

In bold: genes participating in Tau-mediated toxicity

**Table S6. Clusters functional annotations enrichment**

| **GOTERM-BP-DIRECT** | **Benjamini p-value** | **Gene Symbol^a^** |
| --- | --- | --- |
| **Cluster 1** | |  |
| GO:0006468~protein phosphorylation | 1,05E-10 | *Src64B, Src42A, CaMKI, dlg1, scrib, par-1, pyd, arm, Cam, Pax, sgg, hop, ksr, pan, par-6, Amph, kay, rhea, hep, CaMKII, cindr, Dab, Diap1, ena, Fmr1, krz, Pka-C1, Rok, Cdk5, cher, Fak, kibra, CycE, Myd88, Nrg, p130CAS, Socs36E, tau, bnl, CASK, jar, Lerp, numb, sdt, aop, Diap2, Mbs, Nrx-IV, RyR, Shroom, Chd64, Fit1, Fit2, hipk, Past1, pbl, raw, Tao, Tl, Atpalpha, cdi, Cdk5alpha, CG7231, cpx, Dmtn, fry, Gbs-70E, Piezo, Ten-m, Usp47* |
| GO:0007254~JNK cascade | 4,43E-05 |  |
| GO:0007016~cytoskeletal anchoring at plasma membrane | 6,46E-05 |  |
| GO:0046425~regulation of JAK-STAT cascade | 3,50E-03 |  |
| GO:0016055~Wnt signaling pathway | 2,30E-02 |  |
| GO:0007015~actin filament organization | 3,52E-02 |  |
| GO:0035332~positive regulation of hippo signaling | 4,34E-02 |  |
| **Cluster 2** | |  |
| GO:0015991~ATP hydrolysis coupled proton transport | 3,16E-02 | *kuz, CG14184, poe, Vha16-1, Glut1, Meltrin, scb, Vha36-1, Csp, Rab14, Scamp, CG42788, SNF4Agamma, Vap-33A, Vha14-1, Vha44, AMPKalpha, aret, armi, Atg6, Eph, Fs(2)Ket, sli, CG32442, comm, mam, Rab30, Tango5, Tsp96F* |
| **Cluster 3** | |  |
| GO:0000398~mRNA splicing, via spliceosome | 6,40E-04 | *SmB, Prp8, CG6418, snRNP-U1-C, CG10077, CG11985, heph, sm, bl, CG3511, Cwc25, fne, mub, Snp, Atx2, CG42724, CG1806, CG5986, CG6701, Lar, skd, Usp10, CG8108, l(3)L1231, MED14, qkr58E-3, Syn1* |
| **Cluster 4** | |  |
| GO:0007018~microtubule-based movement | 3,48E-10 | *Gl, Dmn, Dlic, Arp10, CG12042, CLIP-190, beta-Spec, dyn-p25, garz, Klp61F, cana, chb, Dhc93AB, tubA84B, TubA67C, bic, Not1, asp, CG6873, dop, milt, Nuf2, Tis11* |
| GO:0007067~mitotic nuclear division | 4,79E-04 |  |
| GO:0051028~mRNA transport | 5,76E-04 |  |
| GO:0008090~retrograde axonal transport | 2,80E-03 |  |
| GO:0007052~mitotic spindle organization | 3,26E-03 |  |
| **Cluster 5** | |  |
| GO:0002181~cytoplasmic translation | 1,25E-02 | *RpS21, RpS10a, Elf, eRF1, RpLP0, RpLP1, Smg5, F1, Hsc70Cb, Apf, CG12050, CG3735, DnaJ-1, Hop, Tcp-1eta, Hr39, unc-45* |
| **Cluster 6** | |  |
| GO:0000087~mitotic M phase | 3,48E-02 | *stg, csul, twe, Wee1, Dp, His2A, Su(z)2, CycJ, wdb* |
| GO:0000278~mitotic cell cycle | 4,81E-02 |  |
| **Cluster 7** | |  |
| GO:0007269~neurotransmitter secretion | 2,62E-02 | *Gdi, Prosalpha7, Prosbeta2, Rpn9, bru, Nelf-E, Nsf2, Rab3-GEF* |
| GO:0016192~vesicle-mediated transport | 3,14E-02 |  |
| **Cluster 8** | |  |
| GO:0015012~heparan sulfate proteoglycan biosynthetic process | 1,20E-03 | *ttv, dally, CG14621, frc, Hs6st, oxt, smid* |

^a^: The genes are sorted by descending degree.

1. **Supplementary References**

1. Jackson, G. R., Wiedau-Pazos, M., Sang, T.-K., et al. (2002) Human wild-type tau interacts with wingless pathway components and produces neurofibrillary pathology in Drosophila. *Neuron*, **34**, 509–519.

2. Shulman, J. M. and Feany, M. B. (2003) Genetic modifiers of tauopathy in Drosophila. *Genetics*, **165**, 1233–1242.

3. Karsten, S. L., Sang, T.-K., Gehman, L. T., et al. (2006) A genomic screen for modifiers of tauopathy identifies puromycin-sensitive aminopeptidase as an inhibitor of tau-induced neurodegeneration. *Neuron*, **51**, 549–560.

4. Blard, O., Feuillette, S., Bou, J., et al. (2007) Cytoskeleton proteins are modulators of mutant tau-induced neurodegeneration in Drosophila. *Hum. Mol. Genet.*, **16**, 555–566.

5. Shulman, J. M., Chipendo, P., Chibnik, L. B., et al. (2011) Functional screening of Alzheimer pathology genome-wide association signals in Drosophila. *Am. J. Hum. Genet.*, **88**, 232–238.

6. Ambegaokar, S. S. and Jackson, G. R. (2011) Functional genomic screen and network analysis reveal novel modifiers of tauopathy dissociated from tau phosphorylation. *Hum. Mol. Genet.*, **20**, 4947–4977.

7. Chapuis, J., Hansmannel, F., Gistelinck, M., et al. (2013) Increased expression of BIN1 mediates Alzheimer genetic risk by modulating tau pathology. *Mol. Psychiatry*, **18**, 1225–1234.

8. Shulman, J. M., Imboywa, S., Giagtzoglou, N., et al. (2014) Functional screening in Drosophila identifies Alzheimer’s disease susceptibility genes and implicates Tau-mediated mechanisms. *Hum. Mol. Genet.*, **23**, 870–877.

9. Butzlaff, M., Hannan, S. B., Karsten, P., et al. (2015) Impaired retrograde transport by the Dynein/Dynactin complex contributes to Tau-induced toxicity. *Hum. Mol. Genet.*, **24**, 3623–3637.

10. Lasagna-Reeves, C. A., de Haro, M., Hao, S., et al. (2016) Reduction of Nuak1 Decreases Tau and Reverses Phenotypes in a Tauopathy Mouse Model. *Neuron*, **92**, 407–418.

11. Dourlen, P., Fernandez-Gomez, F. J., Dupont, C., et al. (2017) Functional screening of Alzheimer risk loci identifies PTK2B as an in vivo modulator and early marker of Tau pathology. *Mol. Psychiatry*, **22**, 874–883.

12. Dourlen, P. (2017) Identification of Tau Toxicity Modifiers in the Drosophila Eye. *Methods Mol. Biol.*, **1523**, 375–389.

13. Gusareva, E. S., Twizere, J.-C., Sleegers, K., et al. (2018) Male-specific epistasis between WWC1 and TLN2 genes is associated with Alzheimer’s disease. *Neurobiol. Aging*, **72**, 188.e3-188.e12.
