# Supplementary material for: A Connected Network of Interacting Proteins Is Involved in Human-Tau Toxicity in Drosophila
Source: Front Neurosci. 2020 Feb 11;14:68. doi: 10.3389/fnins.2020.00068 (PMC7026268; doi:10.3389/fnins.2020.00068)
Supplement: Supplementary file 2 [file Data_Sheet_1.pdf]

Figure S1.

A )

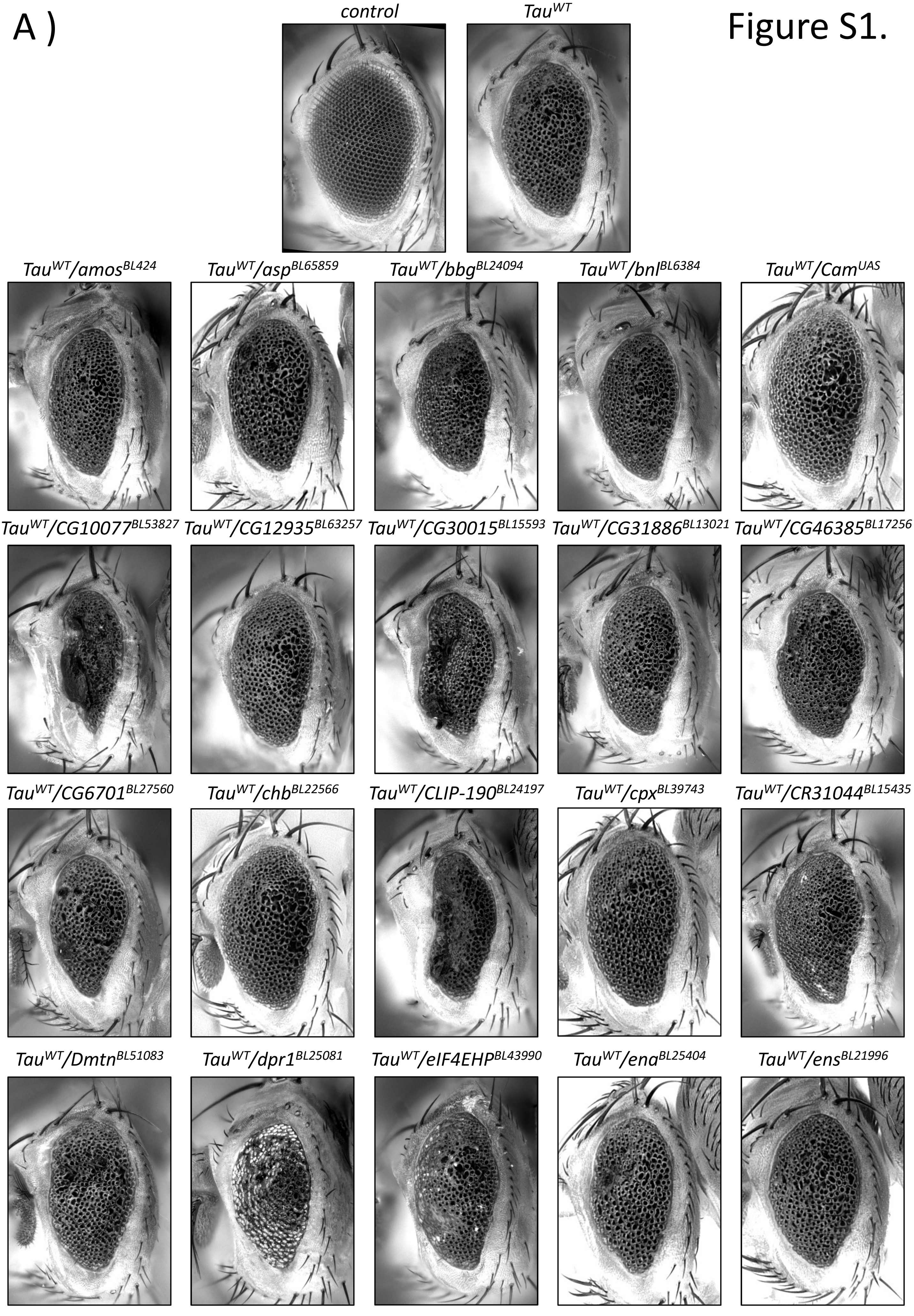
control
$Tau^{WT}$
$Tau^{WT}/amos^{BL424}$
$Tau^{WT}/asp^{BL65859}$
$Tau^{WT}/bbg^{BL24094}$
$Tau^{WT}/bnl^{BL6384}$
$Tau^{WT}/Cam^{UAS}$
$Tau^{WT}/CG10077^{BL53827}$
$Tau^{WT}/CG12935^{BL63257}$
$Tau^{WT}/CG30015^{BL15593}$
$Tau^{WT}/CG31886^{BL13021}$
$Tau^{WT}/CG46385^{BL17256}$
$Tau^{WT}/CG6701^{BL27560}$
$Tau^{WT}/chb^{BL22566}$
$Tau^{WT}/CLIP\text{-}190^{BL24197}$
$Tau^{WT}/cpx^{BL39743}$
$Tau^{WT}/CR31044^{BL15435}$
$Tau^{WT}/Dmtn^{BL51083}$
$Tau^{WT}/dpr1^{BL25081}$
$Tau^{WT}/eIF4EHP^{BL43990}$
$Tau^{WT}/ena^{BL25404}$
$Tau^{WT}/ens^{BL21996}$

B )

Figure S1.

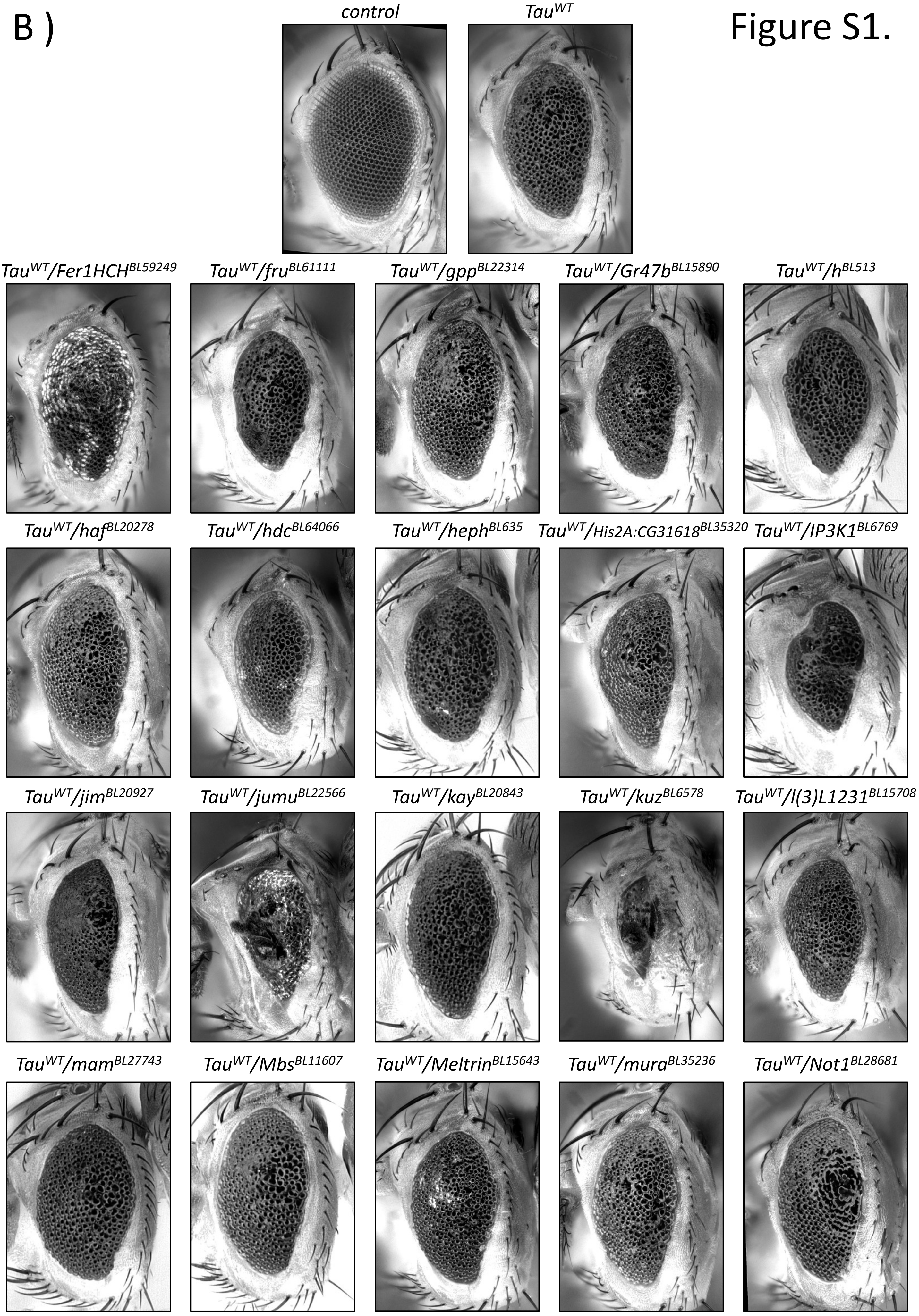
control
$Tau^{WT}$
$Tau^{WT}/Fer1HCH^{BL59249}$
$Tau^{WT}/fru^{BL61111}$
$Tau^{WT}/gpp^{BL22314}$
$Tau^{WT}/Gr47b^{BL15890}$
$Tau^{WT}/h^{BL513}$
$Tau^{WT}/haf^{BL20278}$
$Tau^{WT}/hdc^{BL64066}$
$Tau^{WT}/heph^{BL635}$
$Tau^{WT}/His2A:CG31618^{BL35320}$
$Tau^{WT}/IP3K1^{BL6769}$
$Tau^{WT}/jim^{BL20927}$
$Tau^{WT}/jumu^{BL22566}$
$Tau^{WT}/kay^{BL20843}$
$Tau^{WT}/kuz^{BL6578}$
$Tau^{WT}/l(3)L1231^{BL15708}$
$Tau^{WT}/mam^{BL27743}$
$Tau^{WT}/Mbs^{BL11607}$
$Tau^{WT}/Meltrin^{BL15643}$
$Tau^{WT}/mura^{BL35236}$
$Tau^{WT}/Not1^{BL28681}$

C )

Figure S1.

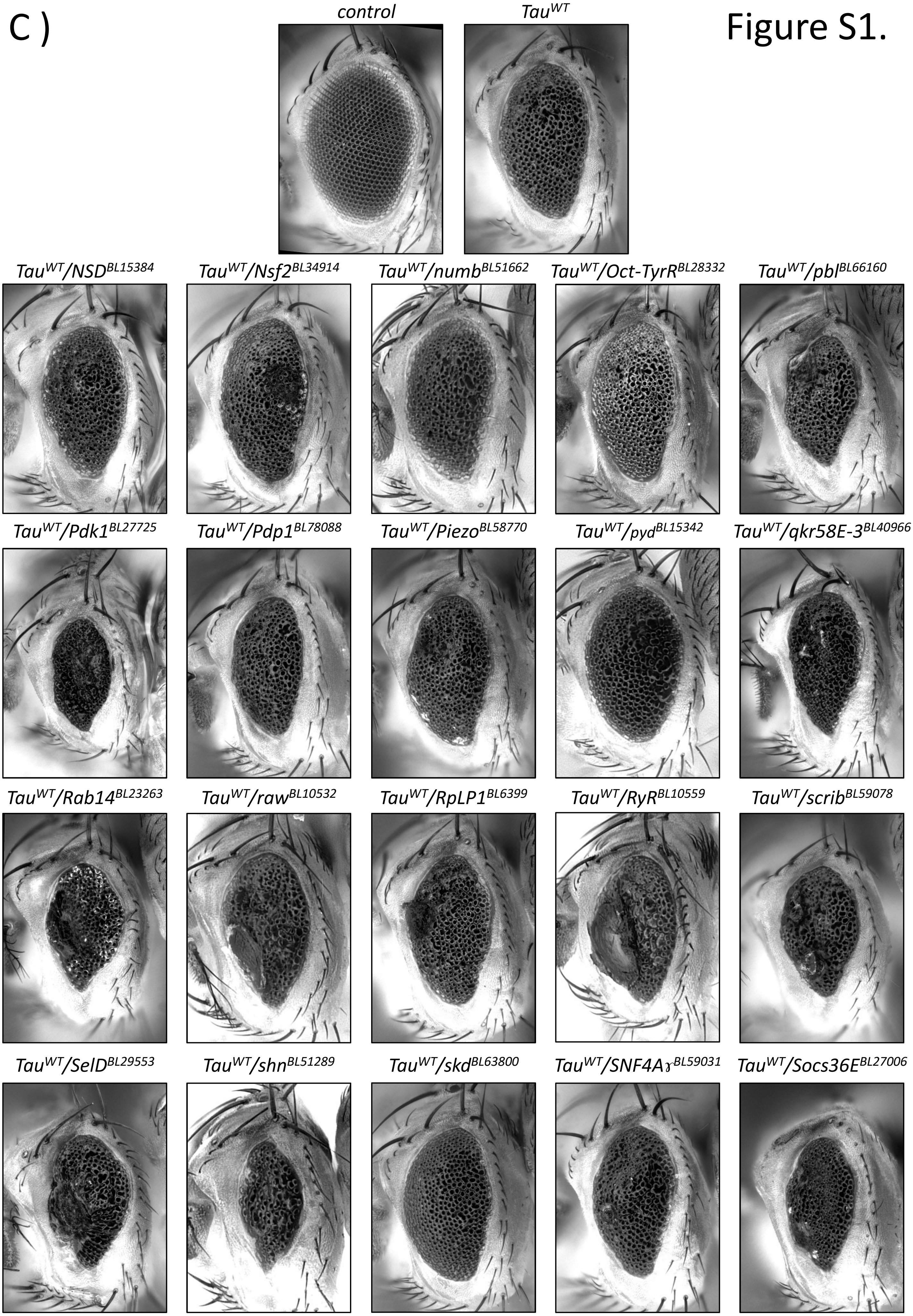
*control*
*$Tau^{WT}$*
*$Tau^{WT}$/NSD$^{BL15384}$*
*$Tau^{WT}$/Nsf2$^{BL34914}$*
*$Tau^{WT}$/numb$^{BL51662}$*
*$Tau^{WT}$/Oct-TyrR$^{BL28332}$*
*$Tau^{WT}$/pbl$^{BL66160}$*
*$Tau^{WT}$/Pdk1$^{BL27725}$*
*$Tau^{WT}$/Pdp1$^{BL78088}$*
*$Tau^{WT}$/Piezo$^{BL58770}$*
*$Tau^{WT}$/pyd$^{BL15342}$*
*$Tau^{WT}$/qkr58E-3$^{BL40966}$*
*$Tau^{WT}$/Rab14$^{BL23263}$*
*$Tau^{WT}$/raw$^{BL10532}$*
*$Tau^{WT}$/RpLP1$^{BL6399}$*
*$Tau^{WT}$/RyR$^{BL10559}$*
*$Tau^{WT}$/scrib$^{BL59078}$*
*$Tau^{WT}$/SelD$^{BL29553}$*
*$Tau^{WT}$/shn$^{BL51289}$*
*$Tau^{WT}$/skd$^{BL63800}$*
*$Tau^{WT}$/SNF4Aγ$^{BL59031}$*
*$Tau^{WT}$/Socs36E$^{BL27006}$*

Figure S1.

D )

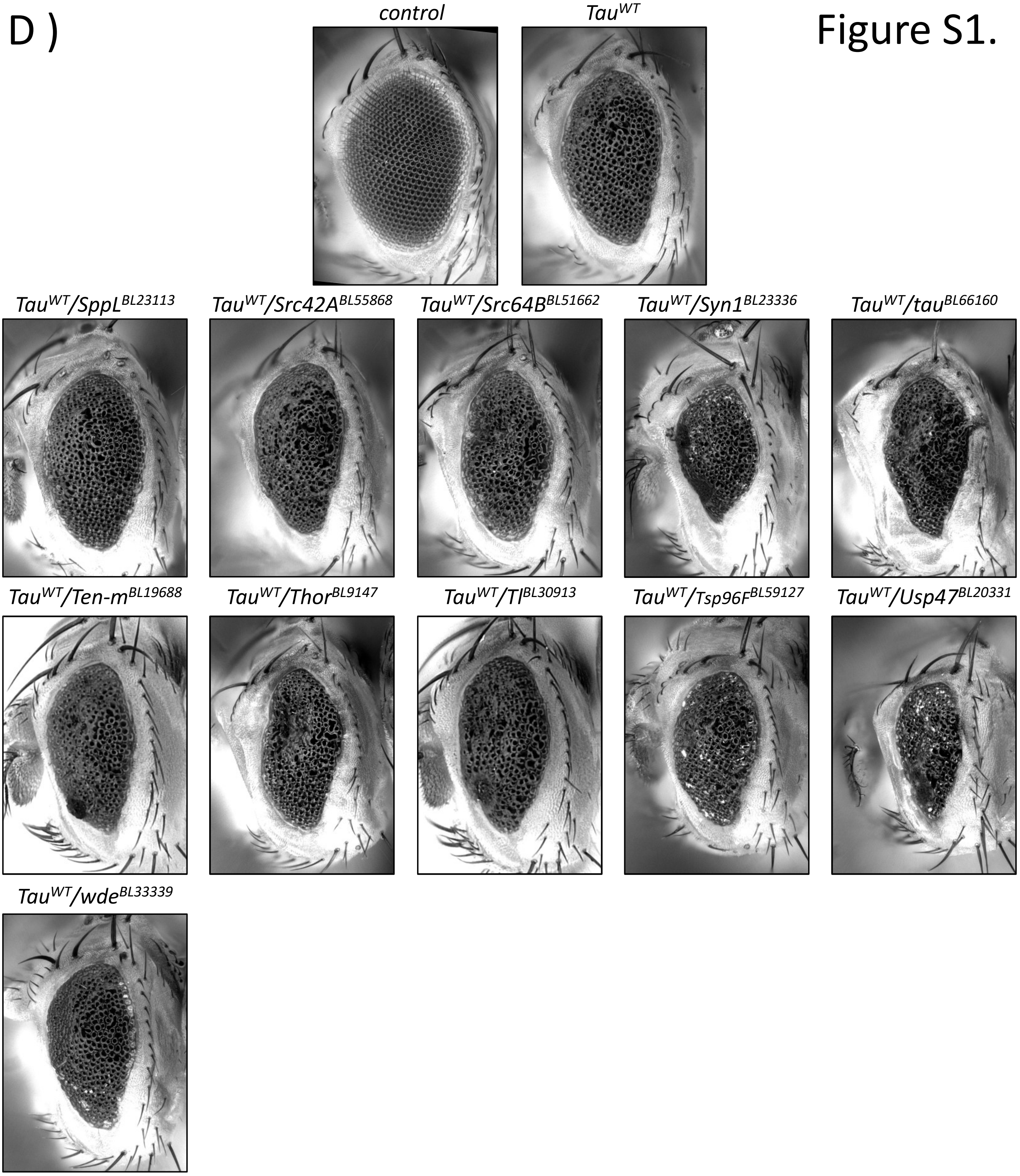

control
Tau$^{WT}$
Tau$^{WT}$/SppL$^{BL23113}$
Tau$^{WT}$/Src42A$^{BL55868}$
Tau$^{WT}$/Src64B$^{BL51662}$
Tau$^{WT}$/Syn1$^{BL23336}$
Tau$^{WT}$/tau$^{BL66160}$
Tau$^{WT}$/Ten-m$^{BL19688}$
Tau$^{WT}$/Thor$^{BL9147}$
Tau$^{WT}$/Tl$^{BL30913}$
Tau$^{WT}$/Tsp96F$^{BL59127}$
Tau$^{WT}$/Usp47$^{BL20331}$
Tau$^{WT}$/wde$^{BL33339}$

# Figure S1.

E )

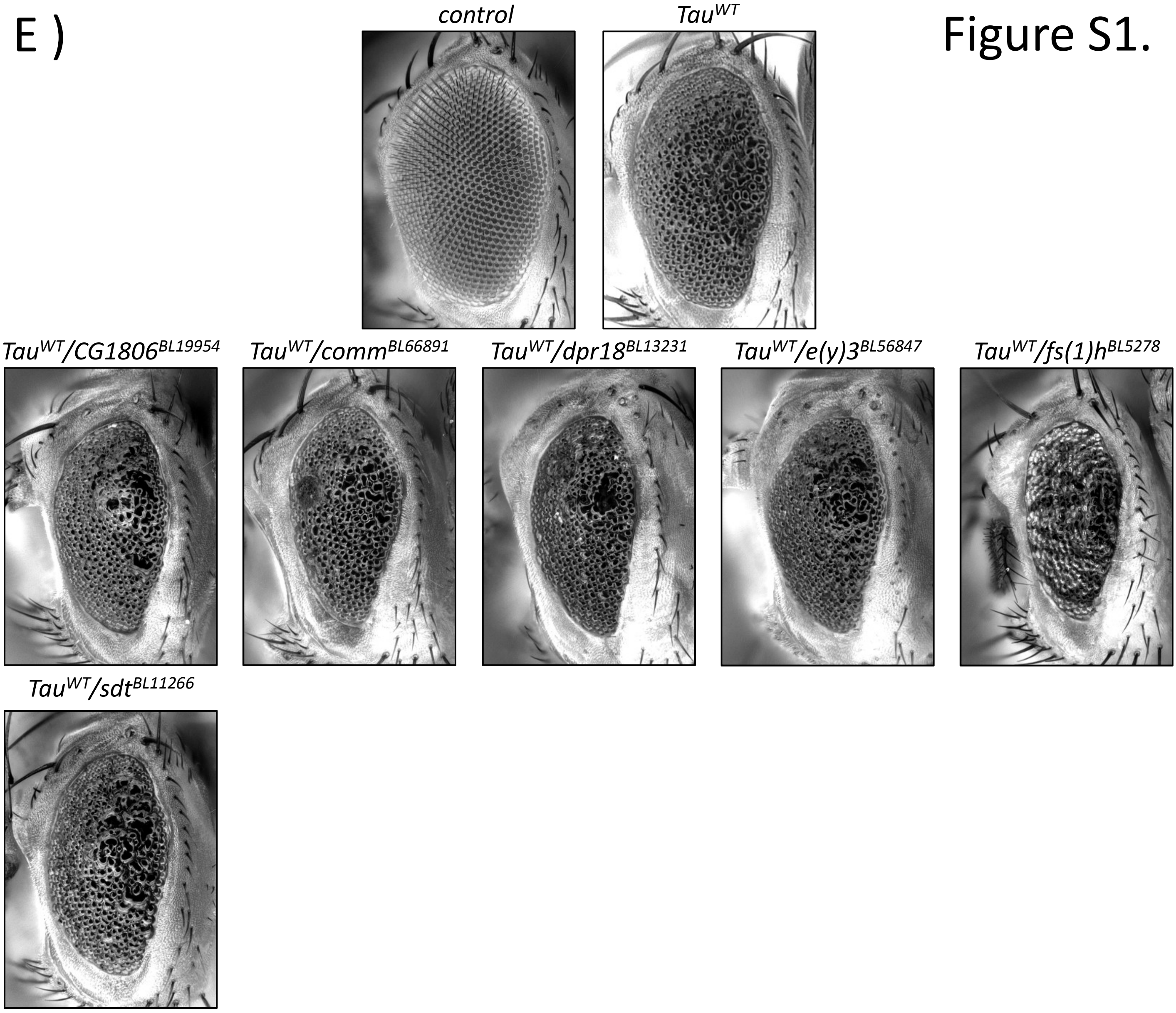

*control*
*Tau*$^{WT}$
*Tau*$^{WT}$*/CG1806*$^{BL19954}$
*Tau*$^{WT}$*/comm*$^{BL66891}$
*Tau*$^{WT}$*/dpr18*$^{BL13231}$
*Tau*$^{WT}$*/e(y)3*$^{BL56847}$
*Tau*$^{WT}$*/fs(1)h*$^{BL5278}$
*Tau*$^{WT}$*/sdt*$^{BL11266}$

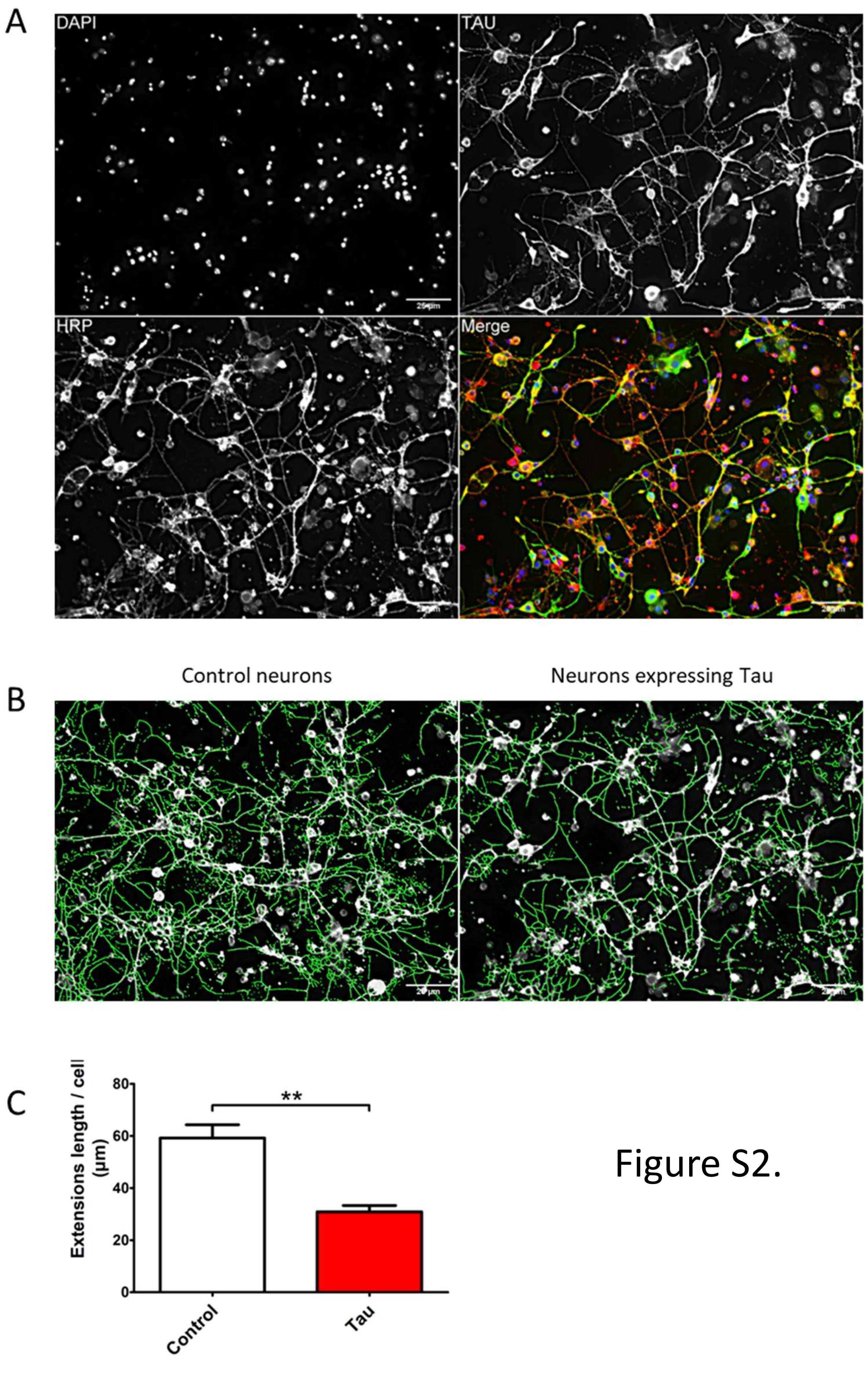

A
DAPI
TAU
HRP
Merge
B
Control neurons
Neurons expressing Tau
C
Extensions length / cell (µm)
80
60
40
20
0
**
Control
Tau


Figure S2.
